# Genome-Scale Consequences of Cofactor Balancing in Engineered Pentose Utilization Pathways in *Saccharomyces cerevisiae*

**DOI:** 10.1371/journal.pone.0027316

**Published:** 2011-11-04

**Authors:** Amit Ghosh, Huimin Zhao, Nathan D. Price

**Affiliations:** 1 Department of Chemical and Biomolecular Engineering, University of Illinois at Urbana-Champaign, Urbana, Illinois, United States of America; 2 Department of Chemistry, University of Illinois at Urbana-Champaign, Urbana, Illinois, United States of America; 3 Center for Biophysics and Computational Biology, University of Illinois at Urbana-Champaign, Urbana, Illinois, United States of America; 4 Institute for Genomic Biology, University of Illinois at Urbana-Champaign, Urbana, Illinois, United States of America; Texas A&M, United States of America

## Abstract

Biofuels derived from lignocellulosic biomass offer promising alternative renewable energy sources for transportation fuels. Significant effort has been made to engineer *Saccharomyces cerevisiae* to efficiently ferment pentose sugars such as D-xylose and L-arabinose into biofuels such as ethanol through heterologous expression of the fungal D-xylose and L-arabinose pathways. However, one of the major bottlenecks in these fungal pathways is that the cofactors are not balanced, which contributes to inefficient utilization of pentose sugars. We utilized a genome-scale model of *S. cerevisiae* to predict the maximal achievable growth rate for cofactor balanced and imbalanced D-xylose and L-arabinose utilization pathways. Dynamic flux balance analysis (DFBA) was used to simulate batch fermentation of glucose, D-xylose, and L-arabinose. The dynamic models and experimental results are in good agreement for the wild type and for the engineered D-xylose utilization pathway. Cofactor balancing the engineered D-xylose and L-arabinose utilization pathways simulated an increase in ethanol batch production of 24.7% while simultaneously reducing the predicted substrate utilization time by 70%. Furthermore, the effects of cofactor balancing the engineered pentose utilization pathways were evaluated throughout the genome-scale metabolic network. This work not only provides new insights to the global network effects of cofactor balancing but also provides useful guidelines for engineering a recombinant yeast strain with cofactor balanced engineered pathways that efficiently co-utilizes pentose and hexose sugars for biofuels production. Experimental switching of cofactor usage in enzymes has been demonstrated, but is a time-consuming effort. Therefore, systems biology models that can predict the likely outcome of such strain engineering efforts are highly useful for motivating which efforts are likely to be worth the significant time investment.

## Introduction

Reconstruction and simulation of genome-scale metabolic networks provides a powerful approach to guide metabolic engineering efforts [1], [2], [3]. Such model guided metabolic engineering approaches have helped to generate rationally modified strains with improved biological functions [4], [5], [6], [7]. In this regard, a genome-scale model (GEM) is highly useful to guide strain design for improved production of chemicals and pharmaceuticals by microorganisms as cell factories. Metabolic GEMs have been used in industrial biotechnology for the production of chemicals, biopolymers, and biofuels, as well as for bioremediation [8], [9]. For example, the metabolic GEM for *Lactococcus lactis* was used to enhance production of diacetyl, a flavoring compound found in dairy products [10]. Moreover, the GEM of *Pseudomonas putida* was used to improve the production of poly-3-hydroxyalkanoates (PHA), which are biodegradable polyesters synthesized to replace petrochemical based plastics [11]. Metabolic GEMs can also be used to guide strain design for improved biofuel production by microorganisms [9], [12]. The scope of applying GEMs to strain design continues to increase, aided by improvements in automated reconstruction of metabolic [13] and integrated regulatory-metabolic networks [14].

Increases in fuel prices coupled with concerns about global warming and energy security, have rekindled interest in producing ethanol and other biofuels from lignocellulosic raw materials such as agriculture and forestry waste [15], [16]. Pretreatment of lignocellulose by chemical or enzymatic methods yields a mixture of hexoses (primarily glucose and mannose) and pentose (primarily D-xylose and L-arabinose, though L-arabinose is typically less abundant than D-xylose) [17]. The fermentation of almost all the available hexose and pentose sugars to biofuels is vital to the overall economics of these processes because this will maximize the yield and minimize the costs associated with waste disposal [18].

The yeast *S. cerevisiae* is often chosen for ethanol production because of its high inhibitor tolerance and its ability to grow at low pH to avoid bacterial contamination [19], [20]. Although well suited for fermentation, *S. cerevisiae* has the substantial drawback that it cannot fully utilize the substrates available from breakdown of plant biomass. That is, wild type *S. cerevisiae* can metabolize the hexose sugars, but not the pentose sugars. To overcome this problem, D-xylose and L-arabinose utilization pathways have been incorporated into the host microorganism. For example, *S. cerevisiae* was engineered to utilize D-xylose using both fungal and bacterial D-xylose utilization pathways [15], [16], [17], [19]. In the bacterial D-xylose utilization pathway, xylose isomerase is used to convert D-xylose to D-xylulose, whereas xylulose kinase is responsible for phosphorylation of D-xylulose to xylulose-5-phosphate [21]. In the fungal D-xylose utilization pathway, D-xylose is converted into D-xylulose by sequential action of two enzymes as found in the D-xylose fermenting fungus *Pichia stipitis* [21]. Xylose reductase (XR) catalyzes the conversion of D-xylose to xylitol and then xylitol is oxidized by xylitol dehydrogenase (XDH) to D-xylulose. The fungal D-xylose utilization pathway was considered to be advantageous for bioethanol production due to its high ethanol productivity compared to the bacterial D-xylose utilization pathway [21].

Similarly, *S. cerevisiae* was engineered to utilize L-arabinose using the fungal reduction/oxidation-based and bacterial isomerization-based pathways. In the bacterial pathway, enzymes L-arabinose isomerase (AI), L-ribulokinase (RK), and L-ribulose-5-P 4-epimerase (R5PE) are responsible for conversion from L-arabinose to L-ribulose, L-ribulose-5-P, and finally D-xylulose-5-P, respectively [22]. In the fungal pathway, aldose reductase catalyzes the conversion of L-arabinose to L-arabinitol, which is converted into L-xylulose by L-arabinitol dehydrogenase (LAD). Finally, L-xylulose is reduced to xylitol by L-xylulose reductase (LXR) [23]. Thus both the D-xylose and L-arabinose pathways converge at xylitol. Low activity of the bacterial L-arabinose pathway and inefficient production of ethanol are the main reasons for using the fungal L-arabinose utilization pathway in *S. cerevisiae* [24].

Previous studies have shown that accumulation of xylitol is ultimately responsible for inefficient production of ethanol in engineered *S. cerevisiae* strains containing the fungal pentose utilization pathways [25]. One possible explanation for the inefficient ethanol production is cofactor imbalance in the introduced pathways [26], as the cofactor specificity of the corresponding enzymes is different, with XR preferring NADPH and XDH preferring NAD^+^. Similarly, the cofactor specificity for L-arabinitol and L-xylulose reductase are NADH and NADPH, respectively. Thus, the operation of these pathways results in a cofactor imbalance that places a demand on the rest of the network to rebalance these cofactor pools. In order to eliminate this cofactor imbalance within the engineered pathways, protein engineering techniques are used to change the cofactor specificity of XDH and LAD to NADP^+^. This change will have the effect of making the pathways redox neutral, meaning that they will not cause an imbalance in cofactor utilization that would then need to be compensated for elsewhere in the network. Aerobically NADP^+^ dependent malic enzymes were used to increase the cystolic or mitochondrial level of NADPH in *S. cerevisiae* [27].

We used a genome-scale constraint-based metabolic model to study for the first time the effects on the global network that result from cofactor balancing associated with the engineered fungal D-xylose and L-arabinose utilization pathways. It was found that cofactor balancing increased predicted pentose sugar utilization efficiency while retaining the flexibility and flux correlation structure of the wild type strain. In addition, the genome-scale model predicts quantitatively an increase in ethanol production of 24.7% that would result from cofactor balancing the introduced pentose utilization pathways – providing strong motivation for the initiation of a laborious and time-consuming enzyme engineering project to change cofactor specificity. Thus, this study provides an example of prospective design of a labor-intensive synthetic biology project using the tools of systems biology.

## Results

### (a) Modified genome-scale models of *S. cerevisiae*

**Figure 1:**
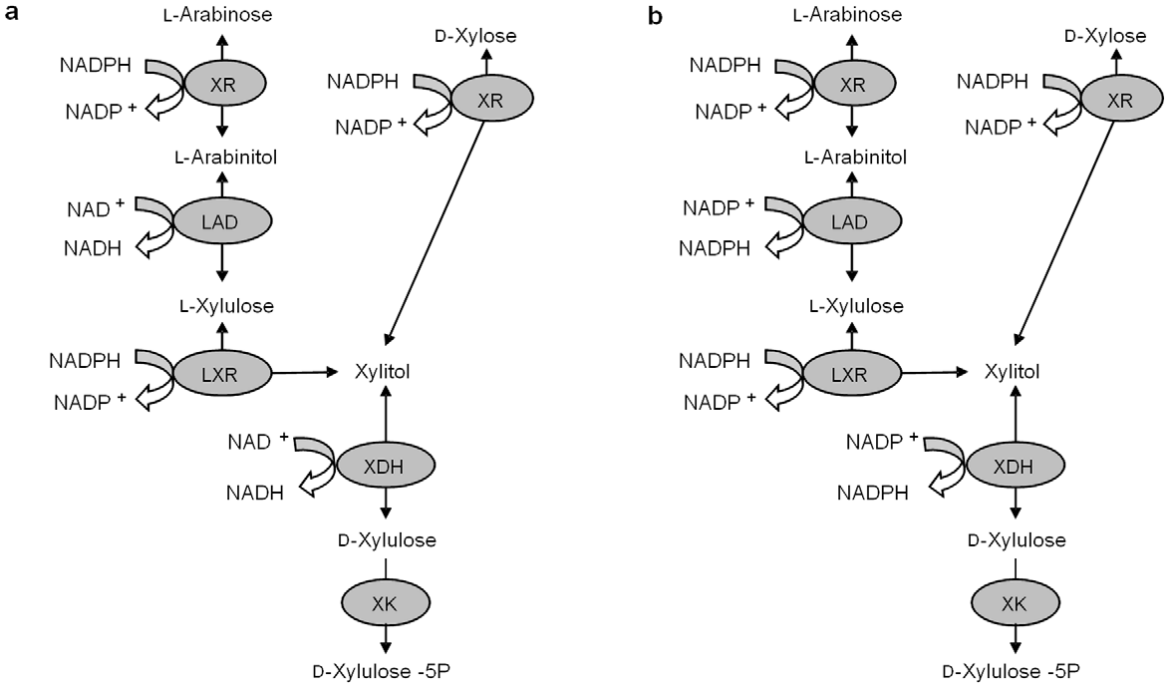
(a) Engineered cofactor imbalanced L-arabinose and D-xylose pathways, (b) Engineered cofactor balanced L-arabinose and D-xylose pathways. XR: Xylose reductase; LAD: L-arabitol dehydrogenase; LXR: L-xylulose reductase; XDH: xylitol dehydrogenase; XK: xylulokinase.

For our simulations, we modified the most recently published genome-scale metabolic model: *S. cerevisiae i*MM904 [28]. To enable D-xylose and L-arabinose utilization, we introduced into the model the same engineered pentose sugar utilizing pathways that we are introducing experimentally into *S. cerevisiae* (Figure 1). The resulting model consisted of 1579 reactions and 1229 metabolites. For better utilization of the substrates L-arabinose and D-xylose, the cofactor specificity of LAD and XDH were changed to NADP^+^ as shown in Figure 1. The carbon sources used for ethanol production were glucose, D-xylose and L-arabinose. Here we have analyzed three models: 1) *wild type* (genome-scale model containing no pentose utilizing pathways); 2) *engineered cofactor imbalanced* (engineered cofactor imbalanced D-xylose and L-arabinose utilization model); and 3) *engineered cofactor balanced* (engineered cofactor balanced D-xylose and L-arabinose utilization model). Dynamic flux balance analysis (DFBA) calculations were done on the engineered cofactor imbalanced model and engineered cofactor balanced model. Flux variability analysis and sampling of the flux space were done on all the three models.

### L-Arabinose and D-xylose utilization

**Figure 2:**
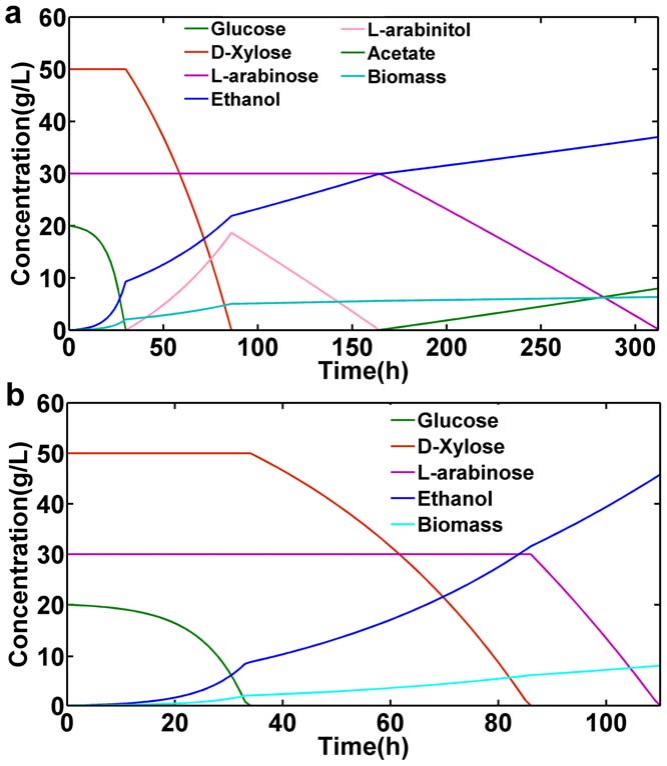
Dynamic flux balance analysis. Glucose, D-xylose, L-arabinose, L-arabinitol, acetate, ethanol and biomass are shown as a function of time for microaerobic *S. cerevisiae* batch growth on glucose, D-xylose, and L-arabinose. (a) engineered cofactor imbalanced (ECI) model. (b) engineered cofactor balanced (ECB) model.

DFBA simulation of batch fermentations for the engineered cofactor imbalanced model was carried out using 20 g/L of glucose in the presence of minimum oxygen for the maximization of biomass production. Apart from glucose, D-xylose and L-arabinose were mixed in 50 g/L and 30 g/L, respectively. The initial concentration and uptake rates for glucose and D-xylose were taken from reported data in literature [29]. The variation of glucose, D-xylose, L-arabinose, ethanol, and cell biomass concentrations as a function of time are shown in Figure 2a. The glucose, oxygen, D-xylose, and L-arabinose consumption rates were 6.06, 0.3, 1.2, and 1.73 mmol/gDCW/h, respectively. Oxygen-limited fermentation of yeast on a mixture of glucose and D-xylose caused a redox imbalance during different cofactor utilization of XR and XDH in xylose metabolism [30]. The oxygen uptake rate was kept very low to simulate the microaerobic environments that maximize ethanol yield. Computationally setting the oxygen uptake rate below 0.3 mmol/gDCW/h, we observed only D-xylose consumption, while L-arabinose was never utilized by the system. This observation argues for a microaerobic condition being optimal for mixed-sugar fermentation. We have constrained our system in such a way that utilization of D-xylose and L-arabinose will start after complete utilization of glucose concentration, to represent the experimentally observed catabolite repression [24], [29]. Glucose is fully utilized within 27 hours of batch simulation, after which D-xylose starts to be utilized. Utilization of D-xylose resulted in ethanol production but at the same time we see significant production of L-arabinitol in the model. Since the reactions in the engineered D-xylose and L-arabinose pathways are reversible, the production of xylitol may lead to L-arabinitol production forcing the reaction to go in the reverse direction of the engineered L-arabinose utilization pathway. Experimentally, pentose-fermenting *S. cerevisiae* strains have shown significant amount of L-arabinitol accumulation [23]. The L-arabinitol production is maximal when D-xylose is consumed completely. After complete consumption of D-xylose, the system utilizes L-arabinitol for growth and not L-arabinose. Since the NADPH load of converting L-arabinose to L-arabinitol leads to a lower predicted growth rate, the L-arabinitol formed during D-xylose consumption is utilized as the preferred carbon source for cell growth. The utilization of L-arabinitol is slow and the model simulated almost 86 hours for complete consumption. The delay in utilization of L-arabinose after D-xylose depletion was mainly due to cofactor imbalance created due to different coenzyme specificity of XR and XDH. This cofactor imbalance led to the built-up L-arabinitol being fully utilized before the L-arabinose began to be utilized. The L-arabinose was then utilized at a slower rate compared to D-xylose but at a similar rate to L-arabinitol. Furthermore, L-arabinose utilization was computed to be accompanied with small amount of acetate production. A reduction in the consumption rate of pentose sugars from D-xylose to L-arabinose may be related to cofactor utilization as mentioned previously [31], [32].

### L-Arabinose and D-xylose utilization with a cofactor balanced pathway

DFBA simulates extracellular L-arabinitol accumulation due to the difference in cofactor specificity between XR with NADPH and XDH with NAD^+^. The difference in cofactor specificity leads to additional demands on the metabolic network to maintain intracellular redox balance. Extracellular L-Arabinitol accumulation in the simulations resulted in inefficient utilization of D-xylose/L-arabinose and decreased ethanol yield. This cofactor imbalance issue can be addressed by two different protein engineering strategies. One of them is to change the cofactor specificity of LAD and XDH from NAD^+^ dependent to NADP^+^ dependent, whereas the other strategy involves the change of cofactor specificity of XR and LXR from NADPH dependent to NADH dependent. We simulated both strategies and found that the results were equivalent. Thus, only the strategy of switching the cofactor specificity of LAD and XDH from NAD^+^ dependent to NADP^+^ to make the pathways redox neutral will be shown here (Figure 1). Cofactor specificity of *Pichia stipitis* XDH (psXDH) has been successfully changed experimentally through protein engineering for efficient fermentation of D-xylose to ethanol using recombinant *S. cerevisiae* [33].

Simulation of ethanol fermentation from mixed sugars was performed with 20 g/L glucose, 50 g/L D-xylose, and 30 g/L L-arabinose as carbon sources. The variation of glucose, D-xylose, L-arabinose, ethanol, and cell biomass concentrations as a function of time are shown in Figure 2b. We kept the oxygen consumption rate to a low value of 0.01 mmol/gDCW/h. In all versions of the model, we found lowering the oxygen uptake rate resulted in increased ethanol production, as would be expected from experimental behavior of *S. cerevisiae*. We simulated the batch processes with both the cofactor imbalanced and cofactor balanced pentose utilization pathways. Here we constrained our model to utilize L-arabinose immediately after D-xylose. The genome-scale model predicted that the strain with the engineered cofactor balanced pathway would have significantly higher ethanol production (46 g/L ethanol) than the strain with the cofactor imbalanced pathway (36.9 g/L of ethanol). The engineered cofactor balanced strain showed significant increase in biomass of 8.03 g/L compared to the cofactor imbalanced strain of 6.27 g/L of biomass. This represents a 24.7% increase in ethanol production from the same amount of substrate, which provides strong motivation for experimental efforts to modify the cofactor specificity – typically a long and laborious process. Interestingly, extracellular L-arabinitol accumulation is not predicted when the engineered cofactor balanced pathway is introduced, leading to the immediate utilization of L-arabinose after complete consumption of D-xylose. Furthermore, the L-arabinose consumption rate is faster than is found with the cofactor imbalanced pathways. Also of critical importance to overall performance, the total time required to consume the mixed sugars glucose, D-xylose, and L-arabinose is predicted to be just 94 hours for the strain with the cofactor balanced pathways compared to 312 hours for the strain with the cofactor imbalanced pathways. Thus by cofactor balancing the metabolic network has improved ethanol production from 36.9 g/L to 46 g/L and simultaneously reduces the utilization time from 312 hours to 94 hours. The cofactor balanced pathway thus has the potential to improve the engineered *S. cerevisiae* strain for ethanol production.

### (b) Comparison of *in silico* predictions and experimental measurements

**Figure 3:**
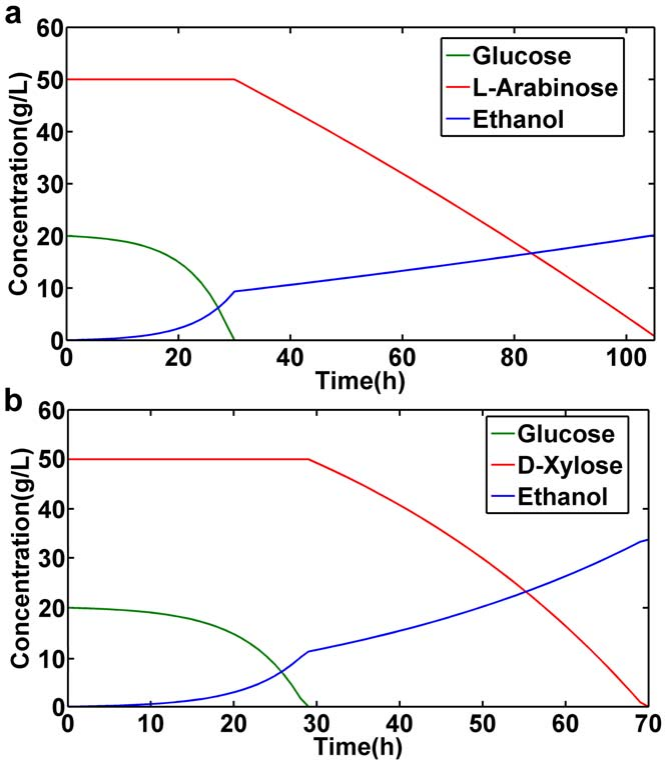
Dynamic flux balance analysis. The glucose, D-xylose and ethanol are shown as a function of time for anaerobic *S. cerevisiae* batch growth on glucose and D-xylose. (a) Without cofactor balance with engineered D-xylose pathway. (b) With cofactor balanced D-xylose pathway.

Of key importance in using the model to guide an experimental program is to ascertain whether the predictions made from the model are generally reliable. The genome-scale *S. cerevisiae* metabolic model has been extensively validated [34], [35], and we will focus on specific predictions closest to our aims in the present study. We also compared results with a *S. cerevisiae* strain [29] that has previously been engineered to metabolize D-xylose, using a modified genome-scale metabolic model with the engineered D-xylose pathway introduced. Simulation of batch fermentations on glucose and D-xylose were compared with experimental results [29]. The consumption of glucose and D-xylose, as well as the production of ethanol were compared over time in batch culture and simulation (Figure 3a). Glucose is utilized first and then D-xylose is consumed. The experimentally measured uptake rates for glucose and D-xylose were used in batch simulation. The concentration of glucose and D-xylose used for the culture are 20 g/L and 50 g/L, respectively. The ethanol yield was 0.29 g ethanol (g consumed sugars)^−1^. Experimental batch fermentation under the same condition as with the wild type strain of *S. cerevisiae* consumed 20 and 30.4 g/L of glucose and D-xylose, respectively and produced 16.8 g/L of ethanol [29] (Table 1). These measurements agree well with our theoretical prediction of 17.6 g/L of ethanol for the same D-xylose consumption as the experimental measurement (Table 1). Moreover, the time taken to consume D-xylose was more than 75 hours for both the prediction and experimental measurement. The xylose transport affinity in S. cerevisiae is very low compared to glucose. Previous studies have shown that transport may control xylose utilization in recombinant *S. cerevisiae* [36]. However the rate at which transport controls the xylose uptake rate is dependent on both the substrate concentration and the downstream pathways. The overall xylose transport in S. cerevisiae was kinetically modeled *in silico* as a function of extracellular glucose concentration [37]. The xylose utilization can be further improved at low glucose concentration compared to xylose concentration. One main reason for the slow rate of D-xylose consumption is presumably that the D-xylose and L-arabinose pathways are not redox balanced. The redox balanced pathways in the network perform much better, since cofactor balancing in the rest of the network remains similar to that of the wild type strain. The cofactor balanced network model behaved much closer to the wild type model and is further verified using flux variability analysis and by comparing the reaction correlation patterns.

**Table 1:** Comparison of model prediction with experimentally measured ethanol production and consumed substrates.

Substrates or Products	Cofactor Imbalanced		Cofactor Balanced	
	Expt (g/L)	Model (g/L)	Expt (g/L)	Model (g/L)
Glucose Consumed	20	20	20	20
Xylose Consumed	30.4	50	46.1	50
Ethanol Produced	16.7	17.6	25.3	33

NAD^+^ generated during the conversion of D-xylose to xylitol can be utilized by NAD^+^ specific XDH. Recently, the batch fermentations were done on a recombinant *S. cerevisiae* strain containing the XR-XDH pathway from *P. stipitis* with engineered NADH-specific XR (YPS270R) [29]. Simulations of batch fermentation were carried out with the cofactor balanced D-xylose pathway (Figure 3b) and compared with experimental results. The simulation used 70 g/L sugar and produced 33 g/L ethanol, whereas YPS270R was reported to consume 66.1 g/L sugar and produced 25.3 g/L ethanol (Table 1). Experimental results showed reduced xylitol accumulation which may be due to the small difference in cofactor specificity. The ethanol production is computed to be further improved to 33 g/L when a NADH-specific XR is used.

### (c) Range of flux variability within the genome-scale network of *S. cerevisiae*

**Figure 4:**
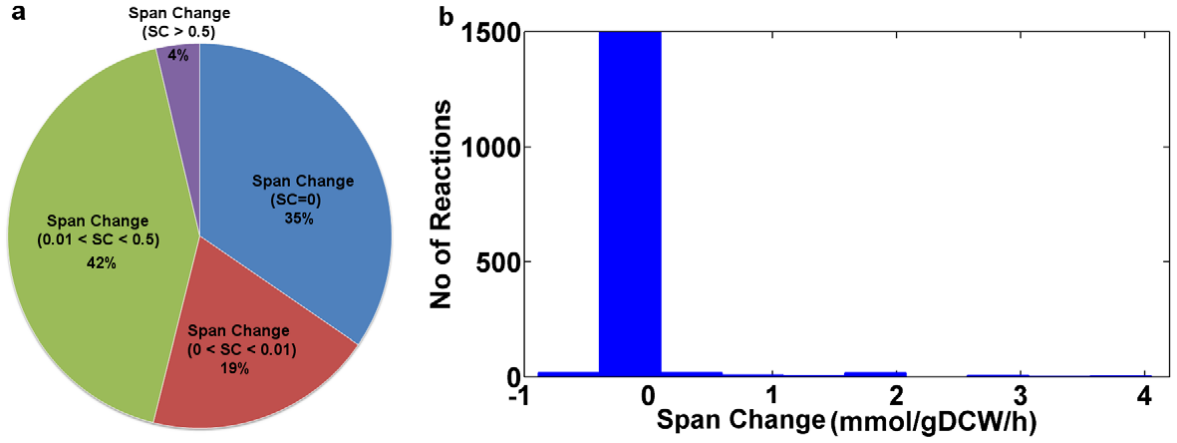
The flux span is obtained using maximum and minimum allowable flux values for all reactions. The difference of flux span between engineered cofactor imbalanced (ECI) and engineered cofactor balanced (ECB) models is given by SC = Span_ECI_ -Span_ECB_. The relative change in flux span between ECI and ECB models are (a) grouped into SC > 0.5, 0.01 < SC < 0.5, 0.0 < SC < 0.01 and SC = 0 with 4%, 42%, 19% and 35% of total reactions respectively, in the entire metabolic network, and (b) plotted as histogram with span change on x-axis and number of reactions on the y-axis.

Growth rate was chosen as the objective function for the optimization of the *S. cerevisiae* genome-scale metabolic network. At optimal growth, a range of variability exists in each flux in the network in terms of the range of flux values it can achieve at steady-state. First, the maximum value of the biomass objective function was calculated and then the maximum value of biomass was used as a constraint when calculating the feasible range of reaction fluxes by subsequent maximization and minimization of each reaction flux. We have applied this technique, known as Flux Variability Analysis (FVA) [38], to determine the maximum and minimum flux values for all the reactions in a given model while satisfying FBA mass balance conditions and all other constraints on the system. The glucose, oxygen, D-xylose, and L-arabinose consumption rates were 6.06, 0.3, 1.2, and 1.73 mmol/gDCW/h, respectively for all the three models. The flux range for each reaction at optimal growth was computed for each of the three above-mentioned models. The reactions affected due to cofactor balancing are selected based on range of flux values, calculated using maximum and minimum allowed fluxes for each reaction. The difference of flux span between engineered cofactor imbalanced (ECI) and engineered cofactor balanced (ECB) models is given by SC = Span_ECI_ -Span_ECB_. A difference flux span or span change (SC) was calculated between engineered cofactor imbalanced and engineered cofactor balanced models reactions, and are grouped into four categories, SC > 0.5, 0.01 < SC < 0.5, 0.0 < SC < 0.01 and SC = 0 with 4%, 42%, 19% and 35% of total reactions respectively, in the entire metabolic network (Figure 4a, Table S1). Almost 65% of the total reactions are affected due to cofactor balancing of the metabolic network. A majority of the reactions affected lie between 0.01-0.5 and only few of them changed drastically (Figure 4b). Reactions that showed a reaction flux span change of more than 2.8 mmol/gDCW/h were chosen for further analysis. The ten selected reactions (PGK, GAPD, PGM, ENO, GLYCDy, DHAK, G3PT, G3PD1ir, PYK, PYRDC) were those whose flux variability was most changed amongst the wild type model, engineered cofactor imbalanced model, and engineered cofactor balanced model (Table 2). The majority of the reactions most affected by cofactor balancing are involved in glycolysis and phospholipid biosynthesis. The flux variability for these selected reactions was less than 2.46 and 7.46 mmol/gDCW/h for the wild type model and the engineered cofactor imbalanced model, respectively. Interestingly, the span of selected reactions for the wild type model is less than the engineered cofactor imbalanced model. The increase in variability in reaction fluxes of the engineered cofactor imbalanced model is associated with reduction in cell growth, which eventually affects the overall ethanol production. The engineered cofactor balanced model has a flux span for the selected reactions with less than or equal to 3.38 mmol/gDCW/h and were much closer to those in the wild type model. Thus, cofactor balancing was responsible for the reduction in flux variability of the reactions.

**Table 2:** The minimum and maximum allowable flux values for each reaction in the wild type, engineered cofactor imbalanced, and engineered cofactor balanced models were obtained using Flux Variability Analysis (FVA).

Reaction	Wild type model			Engineered cofactor imbalanced model			Engineered cofactor balanced model		
	Min Flux*	Max Flux	Span	Min Flux	Max Flux	Span	Min Flux	Max Flux	Span
PGK	-11.40	-10.14	1.26	-16.21	-10.43	5.78	-16.00	-14.27	1.73
GAPD	10.14	11.40	1.26	10.43	16.21	5.78	14.27	16.00	1.73
PGM	-11.40	-10.07	1.33	-16.21	-10.34	5.86	-16.00	-14.17	1.83
ENO	10.07	11.40	1.33	10.35	16.21	5.86	14.17	16.00	1.83
GLYCDy	0.00	1.86	1.86	0.00	5.64	5.64	0.00	2.76	2.76
DHAK	0.00	1.86	1.86	0.00	5.64	5.64	0.00	2.76	2.76
G3PT	0.00	1.86	1.86	0.00	5.64	5.64	0.00	2.76	2.76
G3PD1ir	0.00	2.41	2.41	0.00	6.19	6.19	0.00	3.33	3.33
PYK	10.01	12.46	2.45	10.28	16.79	6.51	14.08	17.54	3.46
PYRDC	7.69	10.89	3.2	8.14	15.6	7.46	10.64	15.26	4.62

*mmol/gDCW/h.

### (d) Sampling candidate flux distributions

The characterization of genome-scale metabolic networks based on reaction flux distribution is an efficient way to differentiate similar metabolic networks with perturbations to the original system, such as the introduction of engineered metabolic pathways. The range of metabolic fluxes that do not violate imposed physico-chemical constraints have been characterized through uniform random sampling of the steady-state flux space [39], [40], [41]. Such analyses also enable studying the correlations between pairs of reactions in the metabolic network to study changes in the relationships between fluxes that are due to the introduced pathways.

**Figure 5:**
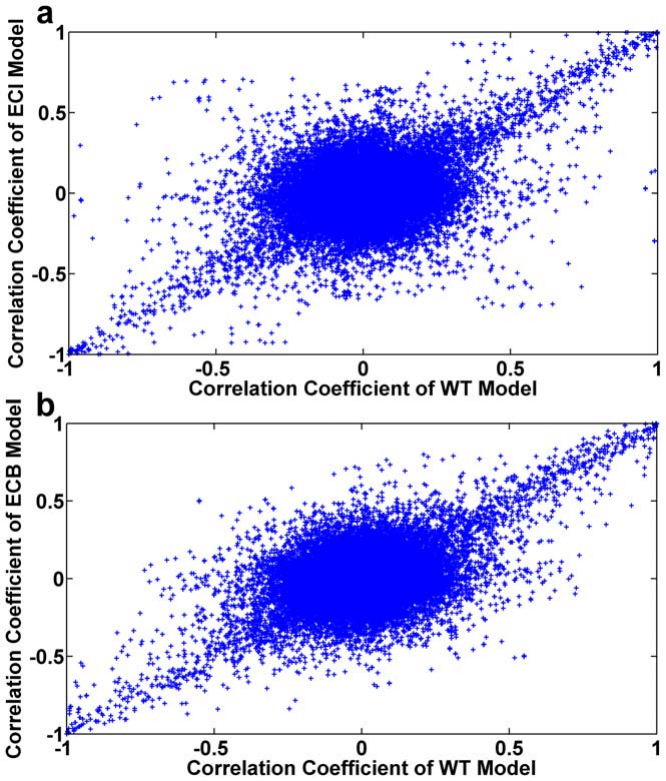
Scatter plots of pairwise correlation coefficients of all fluxes in the metabolic network. (a) Between the wild type (WT) model and the engineered cofactor imbalanced (ECI) model. (b) Between the wild type model and the engineered cofactor balanced (ECB) model.

**Figure 6:**
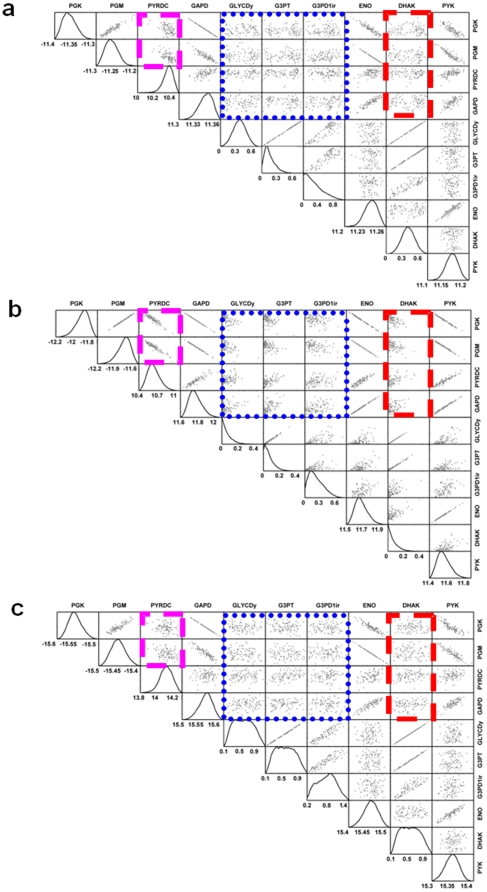
Flux distribution for *S. cerevisiae* obtained using artificial hit and run sampling. Individual flux distribution histograms are along the diagonal and pairwise scatter plots are off-diagonal. The off-diagonal scatter plots show the relationships between fluxes through two reactions. The red, blue and magenta rectangular boxes were made to compare the correlation patterns in wild type model, engineered cofactor imbalanced model and the engineered cofactor balanced model.

The correlation coefficient (r_ij_) between any two fluxes (v_i_ and v_j_) in the network was evaluated using uniform random sampling of the steady-state flux space. The glucose, oxygen, D-xylose, and L-arabinose consumption rates were kept same for the wild type, engineered cofactor imbalanced and cofactor balanced models. The optimal growth rates for wild type, engineered cofactor imbalanced model and engineered cofactor balanced model are 0.14, 0.15, 0.19 hr^−1^ respectively. The correlation pattern obtained from sampling provided an efficient way to distinguish the effects of changes to the metabolic network. Here, the metabolic network has been changed by adding new pathways and changing the cofactor specificity for selected reactions. Pairwise correlation coefficients were calculated between all fluxes (Figure 5). Two scatter plots were made to compare the engineered cofactor imbalanced and the cofactor balanced models with the wild type model. Scatter plot shows the points close to the diagonal are also similar to the reaction correlation present in the wild type model. Interestingly, the scatter plot of the engineered cofactor imbalanced model (Figure 5a) is more scattered compared to the engineered cofactor balanced model (Figure 5b), indicating that cofactor balanced correlation coefficients are close to the wild type correlation coefficients. The correlation coefficients between selected pairs of reactions selected based on the flux variability analysis described above are varying with different models (Table 3). The introduction of xylose and arabinose pathways leads to higher correlation between the reactions in the entire metabolic network. However cofactor balancing the network has shown significant decrease in correlation between reactions. Therefore the reactions are much more flexible in wild type and cofactor balanced models. The overall correlation coefficient between wild type and engineered cofactor balanced models is as high as 0.97, whereas for wild type and engineered cofactor imbalanced models it is 0.72. Thus cofactor balancing the engineered pathways largely restored the correlation relationships of the wild type model based on *in silico* predictions. Furthermore, uniform random samples were used to calculate the probability distribution for fluxes in the metabolic networks of *S. cerevisiae*. The probability distribution of fluxes and pairwise correlation is calculated to show how interdependent these reactions are in the network (Figure 6). The diagonal and off-diagonal represent the flux distribution histograms and pairwise scatter plots, respectively (Figure 6). The off-diagonal scatter plots represent the relationships between two reaction fluxes. For example, the PDYRC and PGK are fully correlated in the engineered cofactor imbalanced model, whereas very low correlations were observed in the wild type and engineered cofactor balanced models. These interdependent reactions are involved in constraining the system to operate at the suboptimal region. In Figure 6a, the red, blue and magenta rectangular boxes were made to compare with the patterns in the wild type model. In the engineered cofactor imbalanced model, the probability distribution shows a decrease in flexibility in reaction fluxes. However, the engineered cofactor balanced model has considerably decreased the correlation among the reaction fluxes (Figure 6c). The highlighted rectangular boxes were marked with highly correlated reaction fluxes in the engineered cofactor imbalanced model but less correlated in the two other models. The shape of the probability distribution is helpful in determining the level of independence between any two fluxes and also identifying highly correlated reaction sets. Thus, by introducing new D-xylose and L-arabinose pathways we see high correlations among the reaction fluxes. The cofactor balancing of the added pathways has diminished the correlations, and the correlation patterns are now much more similar to those in the wild type model.

**Table 3:** Correlation coefficients for reaction pairs were obtained for simulated wild type, engineered cofactor imbalanced and engineered cofactor balanced models.

Reactions	Reactions	Wild type model	Engineered cofactor imbalanced model	Engineered cofactor balanced model
PGK	PYRDC	-0.50	-0.93	-0.32
PGK	GLYCDy	-0.07	-0.53	-0.13
PGK	G3PT	-0.06	-0.53	-0.12
PGK	G3PD1ir	-0.01	-0.39	-0.09
PGK	ENO	-0.92	-1.00	-0.92
PGK	DHAK	-0.07	-0.53	-0.13
PGK	PYK	-0.85	-0.99	-0.87
PGM	PYRDC	-0.45	-0.93	-0.34
PGM	GLYCDy	-0.09	-0.53	-0.06
PGM	G3PT	-0.08	-0.53	-0.05
PGM	G3PD1ir	-0.01	-0.39	-0.03
PGM	DHAK	-0.09	-0.53	-0.06
PYRDC	GAPD	0.50	0.93	0.32
PYRDC	GLYCDy	0.04	0.51	0.22
PYRDC	G3PT	0.04	0.51	0.22
PYRDC	G3PD1ir	-0.05	0.35	0.17
PYRDC	ENO	0.45	0.93	0.34
PYRDC	DHAK	0.04	0.51	0.22
PYRDC	PYK	0.43	0.93	0.31
GAPD	GLYCDy	0.07	0.53	0.13
GAPD	G3PT	0.06	0.53	0.12
GAPD	G3PD1ir	0.01	0.39	0.09
GAPD	DHAK	0.07	0.53	0.13
GLYCDy	G3PD1ir	0.89	0.47	0.89
GLYCDy	ENO	0.09	0.53	0.06
GLYCDy	PYK	0.13	0.53	0.06
G3PT	G3PD1ir	0.89	0.46	0.89
G3PT	ENO	0.08	0.53	0.05
G3PT	PYK	0.12	0.52	0.06
G3PD1ir	ENO	0.01	0.39	0.03
G3PD1ir	DHAK	0.89	0.47	0.89
G3PD1ir	PYK	0.08	0.39	0.03
ENO	DHAK	0.09	0.53	0.06
DHAK	PYK	0.13	0.53	0.06

Engineered cofactor imbalanced model has shown higher correlation coefficient between reaction pairs compared to wild type and cofactor balanced models.

### (g) Flux coupling analysis

The functionally related coupled reaction sets are identified using flux coupling analysis [42] on a genome-scale metabolic network of *S. cerevisiae*. This procedure relies on minimization and maximization of flux ratios to determine the degree of dependency between any two reactions within the network obeying mass balance and boundary constraints. The metabolic reaction pairs can be categorized into the following three groups: i) *fully coupled*: the activity of one reaction fixes the activity for other reaction and vice versa, ii) *directionally coupled*: the activity of one reaction implies the activity of the other but not necessarily the reverse. iii) *partially coupled*: the activity of one reaction implies the activity of the other and vice versa, iv) *uncoupled*: the activity of one reaction does not implies the activity for other reaction and vice versa. These reactions can have any flux ratio and can operate independently.

To measure and compare the extent of coupling of reactions within the metabolic network the upper and lower limit of the flux ratios, R_max_  =  max(ν_1_/ν_2_) & R_min_  =  min(ν_1_/ν_2_) were calculated (Table S2). In most of the reaction sets the partially, directionally and fully coupled reaction pairs have not changed their coupling state in wild type, engineered cofactor imbalanced and cofactor balanced (ECB) models. However the magnitude of the flux ratios R_max_ and R_min_ showed a significant change for the three models. Interestingly the flux ratios for fully coupled reaction pairs PGM-DHAK, PGM-ENO, PGM-G3PT, PGM-GLYCDy, PGM-PGK and PGM-PYK are exactly the same for the wild type and cofactor balanced models. Moreover the flux ratios for partially/directionally coupled reaction pairs PYK-DHAK, PYK-ENO, PYK-G3PT, PYK-GAPD, PYK-GLYCDy, PYK-PGK, PYK-PGM, PYK-PYRDC are similar in magnitudes for wild type and cofactor balanced models, whereas the same flux ratios for engineered cofactor imbalanced model are not matching with other two models. Therefore the flux ratios are shifted with the introduction of new D-xylose and L-arabinose pathways. The cofactor balancing of the added pathways have shown a shift in flux ratios and are much more similar to those in the wild type model.

## Discussion

Computer simulations of GEMs allow the prediction of system behaviors in response to various conditions and can help in strain design for improved biochemical production. We have used a GEM of *S. cerevisiae* to study for the first time the effects on the global network that result from cofactor balancing engineered pathways. In particular, here we focused on the introduction of engineered fungal D-xylose and L-arabinose utilization pathways. The difference in cofactor preference in engineered pathways leads to intracellular redox imbalance. Our studies indicate that cofactor balanced pentose utilization pathways will substantially increase ethanol production. We also observed efficient utilization of the pentose sugars by making the pathways redox balanced – thus reducing the burden placed on the rest of the metabolic network to compensate for the introduction of cofactor imbalanced pathways.

To build a GEM of *S. cerevisiae* that is capable of utilizing pentose sugars, the L-arabinose and D-xylose utilization pathways were introduced into a recently updated genome-scale metabolic model for *S. cerevisiae* (iMM904). The different cofactor specificity, NAD^+^ for LAD and XDH, NADPH for XR and LXR, in engineered D-xylose and L-arabinose utilization pathways has contributed to intercellular redox imbalance, which results in reduced pentose sugar consumption rates and ethanol productivity – which the model predicts as a consequence of the demands placed on the metabolic network to re-balance these co-factors. To address the cofactor imbalance issue, the cofactor specificity was changed computationally from NAD^+^ to NADP^+^ for two enzymes in the D-xylose and L-arabinose utilization pathway including LAD and XDH. The GEM predicts that engineering cofactor balanced pathways will greatly enhance utilization of pentose sugars and increase ethanol production. To validate the model predictions, the computational data were compared with reported experimental data. The experimental ethanol production is 25.3 g/L with 66.1 g/L sugar consumption, while our DFBA calculations have shown 33 g/L of ethanol with 70 g/L of sugar consumption, indicating a very good agreement between model predictions and experimental data.

Based on this revised GEM, flux variability analysis was carried out to further investigate the variation of reaction fluxes in the entire metabolic network. The increase in flexibility of the reaction fluxes resulted in increases in the flux range of the engineered cofactor imbalanced model. Cofactor balancing the engineered pathways restored network flexibility to a state that was very similar to that of the wild type strain. Furthermore, uniform random sampling was carried out to calculate the probability distribution for fluxes through all reactions. Variations in correlation patterns among the reactions were observed with the addition of the engineered cofactor imbalanced and engineered cofactor balanced pathways. The added D-xylose and L-arabinose pathways have increased the correlation among the reactions of the entire metabolic network. Furthermore, pairwise correlation coefficients were calculated for fluxes through all reactions of wild type, engineered cofactor imbalanced, and engineered cofactor balanced models. The wild type network correlation pattern was lost when D-xylose and L-arabinose pathways were added to the cell. Thus, by adding new pathways we have seen an increase in solution space as evident from flux variability analysis. Sampling of allowed flux space has shown an increase in correlations among the reactions. The reactions are interdependent with each other with increase of correlations as evident from the pairwise correlation map. Thus, increased correlation patterns in the engineered cofactor imbalanced model are responsible for the increase in rigidity of the reaction network compared to the wild type network. The addition of new pathways to the cell affected the solution space of the network and also the reaction correlation patterns. The range of possible metabolic network states of the model was much more similar to the wildtype when cofactor balancing was performed on the engineered pathways, suggesting that native regulation in the cell would be much more likely to respond well to the additional carbon flux from the pentoses. Taken together, cofactor balancing increased pentose sugar utilization efficiency while retaining the flexibility and flux correlation structure of the wild type strain. Moreover, model guided cofactor balancing has emerged to help the metabolic engineering of microorganisms to fuels production.

We used a modified genome-scale metabolic model of *S. cerevisiae* to investigate the global effect of cofactor imbalance in engineered pentose sugar utilization pathways. The insights gained from the computational studies will enable the strain design for improved ethanol production in a rational and systematic way. This approach of cellular design promises solutions to some of today's most difficult problems in metabolic engineering of microbes and opens up an alternative perspective to analyze biological systems. Our future goals include the experimental implementation of the strain design proposed herein and the continued development of strategies for model guided biofuel production.

## Materials and Methods

All studies described below were done using the Constraint-Based Reconstruction and Analysis (COBRA) Matlab Toolbox [43]. Functions that were extensively used in our analysis (e.g. dynamic flux balance analysis, flux variability analysis, and flux correlations using sampling) were run in the Matlab environment.

### (a) Flux balance analysis (FBA)

We used the publicly available genome-scale metabolic network *S. cerevisiae i*MM904 [28], with 1577 reactions and 1228 metabolites, as the base model for FBA analysis. FBA was carried out on this model using the biomass as the objective function. The LP problem that is solved is summarized below
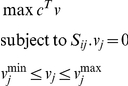
where c is the objective function vector, and v^max^ and v^min^ are the vectors containing the maximum and minimum capabilities of the fluxes.

### (b) Dynamic flux balance analysis (DFBA)

Dynamic flux balance analysis was used to simulate the batch growth of *S. cerevisiae* on glucose, D-xylose and L-arabinose using the COBRA toolbox [43]. DFBA is an approach that simulates batch cultures by incorporating quasi-steady state flux balance calculations at a series of discrete time steps [44]. The batch time was divided into 1000 intervals, solving the optimization problem in an iterative approach based on steady state approximation. At each step, linear optimization was done to predict growth, nutrient uptake, and by-product secretion rates. Furthermore, the concentration was used to calculate the maximum uptake rates of substrates for the next time step. The concentrations for the next time step are calculated from the differential equation given below [44].
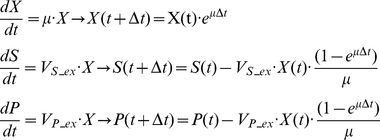



X(t), S(t), P(t) are the biomass, substrate and product concentrations, respectively, at a given time point. V_S_ex_ and V_P_ex_ are the exchange fluxes for the substrate and the product, respectively. During each small time step, Δt, we assume that uptake and secretion rates and μ are constant. The concentration of each external metabolite was computed at each time step, including for glucose, D-xylose, and L-arabinose. The glucose and D-xylose uptake rates were taken from batch experiments [29].

### (c) Flux variability analysis

Flux variability analysis was used to calculate the minimum and maximum allowable flux values for each reaction of the genome-scale metabolic network of *S. cerevisiae*. The range of fluxes is useful for understanding the redundancies in the system. First, the maximum value of the objective function was calculated and this value was used to calculate the feasible range of reaction fluxes by subsequent maximization and minimization of each reaction flux [38]. These were calculated through a series of LP problems. The mathematical formulations for maximization and minimization problems are shown in case 1 and case 2, respectively.



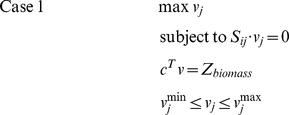


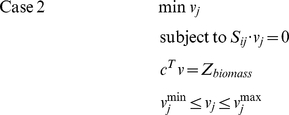
where Z_biomass_ is the value of the biomass objective function. If n is the number of fluxes then the 2n LP problems are to be solved for flux variability analysis. The above method was applied to determine the maximum and minimum flux values for all the reactions in the model while satisfying FBA mass balance conditions and all other constraints on the system [38].

### (d) Flux correlations using sampling

Flux distributions in the genome-scale model corresponding to maximal cell growth were computed using FBA. Uniform random sampling was done to characterize the space of all flux distributions [39]. The genome-scale metabolic networks with small perturbations were differentiated based on the characterization of the solution space using random samplers [45]. The sampling has been done using hit and run samplers with a total of 10 million steps [41]. The correlation coefficient (r_ij_) between any two fluxes (v_i_ and v_j_) in the network was evaluated using random sampling of the steady-state flux space [40]. The correlations between pairs of reactions in the metabolic network were compared with engineered and cofactor balanced models.

### (e) Flux Coupling Analysis

We have applied the previously developed Flux coupling finder for finding coupled reactions sets in constraint-based genome-scale metabolic models [42]. All coupled reactions are identified by calculating the minimum and maximum flux ratio given by R_max_ (max v_1_/v_2_) and R_min_ (min v_1_/v_2_) respectively between two fluxes v_1_ and v_2_. 
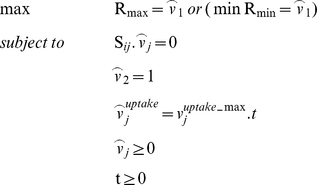



The flux ratios for every pair of fluxes are used to determine different types of coupling shared between the two fluxes v_1_ and v_2_.

(a) If R_min_ = 0 and R_max_ > 0, then reactions are directionally coupled

(b) If (R_max_ - R_min_) > 0, then reactions are partially coupled.

(c) If (R_max_ - R_min_)  =  0, then reactions are fully coupled.

The reaction pairs not coming into these three categories are termed as uncoupled reactions.

## Supporting Information

Table S1 The minimum and maximum allowable flux values obtained for reactions with different values of span change (SC), (a) Span_ECI_ -Span_ECB_ > 0.5, (b) 0.01 <Span_ECI_ -Span_ECB_ < 0.5 and (c) 0.0 <Span_ECI_ -Span_ECB_ < 0.01. The span change or difference of flux span between engineered cofactor imbalanced (ECI) and engineered cofactor balanced (ECB) models is given by, SC = Span_ECI_ -Span_ECB_.
(DOCX)

Table S2 Based on the flux ratio limits R_max_ (max v_1_/v_2_) and R_min_ (min v_1_/v_2_) various types of coupling were identified for wild type, engineered cofactor imbalanced (ECI) and engineered cofactor balanced (ECB) models.
(DOCX)
