# Supplementary material for: Genome-Scale Consequences of Cofactor Balancing in Engineered Pentose Utilization Pathways in Saccharomyces cerevisiae
Source: PLoS One. 2011 Nov 4;6(11):e27316. doi: 10.1371/journal.pone.0027316 (PMC3208632; doi:10.1371/journal.pone.0027316)
Supplement: Table S1 — The minimum and maximum allowable flux values obtained for reactions with different values of span change (SC), (a) SpanECI -SpanECB > 0.5, (b) 0.01 <SpanECI -SpanECB < 0.5 and (c) 0.0 <SpanECI -SpanECB < 0.01. The span change or difference of flux span between engineered cofactor imbalanced (ECI) and engineered cofactor balanced (ECB) models is given by, SC = SpanECI -SpanECB. (DOCX) [file pone.0027316.s001.docx]

**Table S1a.** The minimum and maximum allowable flux values for reaction whose difference of flux span between engineered cofactor imbalanced and engineered cofactor balanced models is given by Span_ECI_ -Span_ECB_ > 0.5.

| **Reaction** | **Wild type**  **model** | | | **Engineered cofactor**  **imbalanced model** | | | **Engineered cofactor**  **balanced model** | | |
| --- | --- | --- | --- | --- | --- | --- | --- | --- | --- |
|  | **Min**  **Flux*** | **Max**  **Flux** | **Span** | **Min**  **Flux** | **Max**  **Flux** | **Span** | **Min**  **Flux** | **Max**  **Flux** | **Span** |
| **EX_co2(e)** | 6.62 | 11.1 | 4.48 | 7.4 | 15.76 | 8.36 | 8.94 | 15.51 | 6.57 |
| **EX_etoh(e)** | 6.51 | 10.61 | 4.1 | 6.48 | 15.3 | 8.82 | 9.03 | 14.91 | 5.88 |
| **EX_h(e)** | 0.45 | 5.49 | 5.04 | 0.5 | 9.98 | 9.48 | 0.63 | 8.05 | 7.42 |
| **EX_h2o(e)** | 1.39 | 3.57 | 2.18 | 0.33 | 4.34 | 4.01 | 1.79 | 4.94 | 3.15 |
| **EX_succ(e)** | 0.00 | 1.96 | 1.96 | 0.00 | 4.65 | 4.65 | 0.00 | 2.94 | 2.94 |
| **EX_xyl-D(e)** | 0.00 | 0.00 | 0.00 | -1.73 | 0.00 | 1.73 | -1.73 | -0.7 | 1.03 |
| **EX_xylt(e)** | 0.00 | 0.00 | 0.00 | 0.00 | 1.45 | 1.45 | 0.00 | 0.94 | 0.94 |
| **ABTt** | 0.00 | 0.00 | 0.00 | -2.93 | 0.00 | 2.93 | -0.94 | 0.00 | 0.94 |
| **CO2t** | -11.1 | -6.62 | 4.48 | -15.76 | -7.4 | 8.36 | -15.51 | -8.94 | 6.57 |
| **DHAK** | 0.00 | 1.86 | 1.86 | 0.00 | 5.64 | 5.64 | 0.00 | 2.76 | 2.76 |
| **DHAPtm** | 0.00 | 2.41 | 2.41 | 0.00 | 5.22 | 5.22 | 0.00 | 3.33 | 3.33 |
| **ENO** | 10.07 | 11.4 | 1.33 | 10.35 | 16.21 | 5.86 | 14.17 | 16 | 1.83 |
| **ETOHt** | -10.61 | -6.51 | 4.1 | -15.3 | -6.48 | 8.82 | -14.91 | -9.03 | 5.88 |
| **G3PD1ir** | 0.00 | 2.41 | 2.41 | 0 | 6.19 | 6.19 | 0 | 3.33 | 3.33 |
| **G3PD1irm** | 0.00 | 2.41 | 2.41 | 0 | 5.22 | 5.22 | 0 | 3.33 | 3.33 |
| **G3PDm** | 0.00 | 2.41 | 2.41 | 0 | 5.22 | 5.22 | 0 | 3.33 | 3.33 |
| **G3PT** | 0.00 | 1.86 | 1.86 | 0 | 5.64 | 5.64 | 0 | 2.76 | 2.76 |
| **G6PDH2** | 0.00 | 0.89 | 0.89 | 0 | 2.41 | 2.41 | 0 | 1.23 | 1.23 |
| **GAPD** | 10.14 | 11.4 | 1.26 | 10.43 | 16.21 | 5.78 | 14.27 | 16 | 1.73 |
| **GLYC3Ptm** | 0 | 2.41 | 2.41 | 0 | 5.22 | 5.22 | 0 | 3.33 | 3.33 |
| **GLYCDy** | 0 | 1.86 | 1.86 | 0 | 5.64 | 5.64 | 0 | 2.76 | 2.76 |
| **GND** | 0 | 0.89 | 0.89 | 0 | 2.41 | 2.41 | 0 | 1.23 | 1.23 |
| **H2Ot** | -3.57 | -1.39 | 2.18 | -4.34 | -0.33 | 4.01 | -4.94 | -1.79 | 3.15 |
| **NADH2-u6cm** | 0 | 2.41 | 2.41 | 0 | 5.22 | 5.22 | 0 | 3.33 | 3.33 |
| **NADH2-u6m** | 0 | 2.41 | 2.41 | 0 | 5.22 | 5.22 | 0 | 3.33 | 3.33 |
| **PC** | 0.13 | 2.39 | 2.26 | 0 | 5.05 | 5.05 | 0.2 | 3.54 | 3.34 |
| **PGK** | -11.4 | -10.14 | 1.26 | -16.21 | -10.43 | 5.78 | -16 | -14.27 | 1.73 |
| **PGL** | 0 | 0.89 | 0.89 | 0 | 2.41 | 2.41 | 0 | 1.23 | 1.23 |
| **PGM** | -11.4 | -10.07 | 1.33 | -16.21 | -10.35 | 5.86 | -16 | -14.17 | 1.83 |
| **PYK** | 10.01 | 12.46 | 2.45 | 10.28 | 16.79 | 6.51 | 14.08 | 17.54 | 3.46 |
| **PYRDC** | 7.69 | 10.89 | 3.2 | 8.14 | 15.6 | 7.46 | 10.64 | 15.26 | 4.62 |
| **RPE** | -0.14 | 0.56 | 0.7 | -1.08 | 1.05 | 2.13 | -1.17 | -0.11 | 1.06 |
| **RPI** | -0.33 | -0.04 | 0.29 | -1.8 | -0.04 | 1.76 | -1.44 | -0.68 | 0.76 |
| **SUCCt2r** | -1.96 | 0 | 1.96 | -4.65 | 0 | 4.65 | -2.94 | 0 | 2.94 |
| **TKT1** | -0.05 | 0.29 | 0.34 | -0.04 | 1.76 | 1.8 | 0.63 | 1.38 | 0.75 |
| **TKT2** | -0.16 | 0.26 | 0.42 | -0.12 | 1.72 | 1.84 | 0.59 | 1.34 | 0.75 |
| **TPI** | 4.71 | 5.71 | 1 | 4.91 | 7.63 | 2.72 | 6.04 | 7.53 | 1.49 |
| **XYLK** | 0 | 0 | 0 | 0 | 2.93 | 2.93 | 1.9 | 2.93 | 1.03 |
| **XYLR** | 0 | 0 | 0 | 0 | 1.73 | 1.73 | 0.7 | 1.73 | 1.03 |
| **XYLTD_D** | 0 | 0 | 0 | 0 | 2.93 | 2.93 | 1.9 | 2.93 | 1.03 |
| **XYLTt** | 0 | 0 | 0 | -1.45 | 0 | 1.45 | -0.94 | 0 | 0.94 |
| **XYLt** | 0 | 0 | 0 | 0 | 1.73 | 1.73 | 0.7 | 1.73 | 1.03 |
| **ALATA_L** | -1.29 | 3.04 | 4.33 | -1.51 | 3.14 | 4.65 | -1.8 | 3.45 | 5.25 |
| **CO2tm** | -2.9 | -0.13 | 2.77 | -3.2 | -0.14 | 3.06 | -4.07 | -0.18 | 3.89 |
| **FBA3** | 0 | 6.92 | 6.92 | 0 | 8.37 | 8.37 | 0 | 9.26 | 9.26 |
| **GLNS** | 0.1 | 1.9 | 1.8 | 0.11 | 2.08 | 1.97 | 0.14 | 2.71 | 2.57 |
| **GLUSx** | 0 | 1.8 | 1.8 | 0 | 1.97 | 1.97 | 0 | 2.57 | 2.57 |
| **MDHm** | -1.88 | 500 | 501.88 | -2.07 | 500 | 502.07 | -2.63 | 500 | 502.63 |
| **OAAt2m** | -500 | 4.07 | 504.07 | -500 | 4.34 | 504.34 | -500 | 5.16 | 505.16 |
| **PFK** | 0 | 6.92 | 6.92 | 0 | 8.37 | 8.37 | 0 | 9.26 | 9.26 |
| **PFK_3** | 0 | 6.92 | 6.92 | 0 | 8.37 | 8.37 | 0 | 9.26 | 9.26 |
| **PPA** | 0 | 1.73 | 1.73 | 0 | 1.91 | 1.91 | 0 | 2.42 | 2.42 |
| **PPM** | -1.23 | 0.62 | 1.85 | -1.35 | 0.68 | 2.03 | -1.73 | 0.86 | 2.59 |
| **PUNP1** | -1.23 | 0.62 | 1.85 | -1.35 | 0.68 | 2.03 | -1.73 | 0.86 | 2.59 |
| **PUNP3** | -1.23 | 0.61 | 1.84 | -1.35 | 0.68 | 2.03 | -1.73 | 0.86 | 2.59 |
| **SLFAT** | -1.22 | 0.57 | 1.79 | -1.35 | 0.63 | 1.98 | -1.72 | 0.81 | 2.53 |
| **TALA** | -6.89 | 0.29 | 7.18 | -7.38 | 1.76 | 9.14 | -8.28 | 1.38 | 9.66 |

**Table S1b.** The minimum and maximum allowable flux values for reaction whose difference of flux span between engineered cofactor imbalanced and engineered cofactor balanced models is given by 0.01 <Span_ECI_ -Span_ECB_ < 0.5.

| **Reaction** | **Wild type**  **model** | | | **Engineered cofactor**  **imbalanced model** | | | **Engineered cofactor**  **balanced model** | | |
| --- | --- | --- | --- | --- | --- | --- | --- | --- | --- |
|  | **Min**  **Flux** | **Max**  **Flux** | **Span** | **Min**  **Flux*** | **Max**  **Flux** | **Span** | **Min**  **Flux** | **Max**  **Flux** | **Span** |
| **EX_2hb(e)** | 0 | 0.49 | 0.49 | 0 | 0.52 | 0.52 | 0 | 0.69 | 0.69 |
| **EX_2mbac(e)** | 0 | 0.21 | 0.21 | 0 | 0.23 | 0.23 | 0 | 0.29 | 0.29 |
| **EX_2mbald(e)** | 0 | 0.3 | 0.3 | 0 | 0.33 | 0.33 | 0 | 0.42 | 0.42 |
| **EX_2mbtoh(e)** | 0 | 0.38 | 0.38 | 0 | 0.41 | 0.41 | 0 | 0.53 | 0.53 |
| **EX_2mppal(e)** | 0 | 0.63 | 0.63 | 0 | 0.77 | 0.77 | 0 | 0.86 | 0.86 |
| **EX_2phetoh(e)** | 0 | 0.19 | 0.19 | 0 | 0.22 | 0.22 | 0 | 0.27 | 0.27 |
| **EX_3c3hmp(e)** | 0 | 0.3 | 0.3 | 0 | 0.39 | 0.39 | 0 | 0.41 | 0.41 |
| **EX_3mbald(e)** | 0 | 0.24 | 0.24 | 0 | 0.32 | 0.32 | 0 | 0.33 | 0.33 |
| **EX_3mop(e)** | 0 | 0.3 | 0.3 | 0 | 0.33 | 0.33 | 0 | 0.42 | 0.42 |
| **EX_4abut(e)** | 0 | 0.3 | 0.3 | 0 | 0.39 | 0.39 | 0 | 0.41 | 0.41 |
| **EX_4abz(e)** | 0 | 0.17 | 0.17 | 0 | 0.19 | 0.19 | 0 | 0.24 | 0.24 |
| **EX_Nbfortyr(e)** | 0 | 0.07 | 0.07 | 0 | 0.08 | 0.08 | 0 | 0.09 | 0.09 |
| **EX_aces(e)** | 0 | 0.42 | 0.42 | 0 | 0.52 | 0.52 | 0 | 0.58 | 0.58 |
| **EX_ala-L(e)** | 0 | 1.23 | 1.23 | 0 | 1.45 | 1.45 | 0 | 1.73 | 1.73 |
| **EX_arg-L(e)** | 0 | 0.12 | 0.12 | 0 | 0.13 | 0.13 | 0 | 0.16 | 0.16 |
| **EX_asn-L(e)** | 0 | 0.25 | 0.25 | 0 | 0.28 | 0.28 | 0 | 0.35 | 0.35 |
| **EX_asp-L(e)** | 0 | 0.61 | 0.61 | 0 | 0.71 | 0.71 | 0 | 0.86 | 0.86 |
| **EX_cit(e)** | 0 | 0.3 | 0.3 | 0 | 0.39 | 0.39 | 0 | 0.41 | 0.41 |
| **EX_cys-L(e)** | 0 | 0.17 | 0.17 | 0 | 0.19 | 0.19 | 0 | 0.23 | 0.23 |
| **EX_dttp(e)** | 0 | 0.08 | 0.08 | 0 | 0.1 | 0.1 | 0 | 0.12 | 0.12 |
| **EX_for(e)** | 0 | 0.14 | 0.14 | 0 | 0.17 | 0.17 | 0 | 0.19 | 0.19 |
| **EX_fum(e)** | 0 | 1.23 | 1.23 | 0 | 1.35 | 1.35 | 0 | 1.73 | 1.73 |
| **EX_g3pc(e)** | 0 | 0.05 | 0.05 | 0 | 0.05 | 0.05 | 0 | 0.07 | 0.07 |
| **EX_gam6p(e)** | 0 | 0.31 | 0.31 | 0 | 0.34 | 0.34 | 0 | 0.43 | 0.43 |
| **EX_glc(e)** | -6.06 | -5.45 | 0.61 | -6.06 | -5.38 | 0.68 | -6.06 | -5.2 | 0.86 |
| **EX_gln-L(e)** | 0 | 0.24 | 0.24 | 0 | 0.3 | 0.3 | 0 | 0.33 | 0.33 |
| **EX_glu-L(e)** | 0 | 0.3 | 0.3 | 0 | 0.39 | 0.39 | 0 | 0.41 | 0.41 |
| **EX_gly(e)** | 0 | 0.49 | 0.49 | 0 | 0.52 | 0.52 | 0 | 0.69 | 0.69 |
| **EX_glyc(e)** | 0 | 0.88 | 0.88 | 0 | 1.08 | 1.08 | 0 | 1.32 | 1.32 |
| **EX_gua(e)** | 0 | 0.07 | 0.07 | 0 | 0.08 | 0.08 | 0 | 0.1 | 0.1 |
| **EX_his-L(e)** | 0 | 0.09 | 0.09 | 0 | 0.11 | 0.11 | 0 | 0.13 | 0.13 |
| **EX_hxan(e)** | 0 | 0.09 | 0.09 | 0 | 0.11 | 0.11 | 0 | 0.12 | 0.12 |
| **EX_iamac(e)** | 0 | 0.17 | 0.17 | 0 | 0.22 | 0.22 | 0 | 0.24 | 0.24 |
| **EX_iamoh(e)** | 0 | 0.3 | 0.3 | 0 | 0.39 | 0.39 | 0 | 0.41 | 0.41 |
| **EX_ibutac(e)** | 0 | 0.32 | 0.32 | 0 | 0.38 | 0.38 | 0 | 0.43 | 0.43 |
| **EX_ibutoh(e)** | 0 | 1.23 | 1.23 | 0 | 1.35 | 1.35 | 0 | 1.73 | 1.73 |
| **EX_id3acald(e)** | 0 | 0.1 | 0.1 | 0 | 0.11 | 0.11 | 0 | 0.13 | 0.13 |
| **EX_ile-L(e)** | 0 | 0.29 | 0.29 | 0 | 0.32 | 0.32 | 0 | 0.41 | 0.41 |
| **EX_ind3eth(e)** | 0 | 0.1 | 0.1 | 0 | 0.12 | 0.12 | 0 | 0.14 | 0.14 |
| **EX_lac-D(e)** | 0 | 0.61 | 0.61 | 0 | 0.68 | 0.68 | 0 | 0.86 | 0.86 |
| **EX_lac-L(e)** | 0 | 0.41 | 0.41 | 0 | 0.46 | 0.46 | 0 | 0.58 | 0.58 |
| **EX_leu-L(e)** | 0 | 0.24 | 0.24 | 0 | 0.3 | 0.3 | 0 | 0.33 | 0.33 |
| **EX_lys-L(e)** | 0 | 0.14 | 0.14 | 0 | 0.17 | 0.17 | 0 | 0.19 | 0.19 |
| **EX_mal-L(e)** | 0 | 1.23 | 1.23 | 0 | 1.35 | 1.35 | 0 | 1.73 | 1.73 |
| **EX_met-L(e)** | 0 | 0.14 | 0.14 | 0 | 0.15 | 0.15 | 0 | 0.19 | 0.19 |
| **EX_nh4(e)** | -1.91 | -0.68 | 1.23 | -2.21 | -0.76 | 1.45 | -2.68 | -0.96 | 1.72 |
| **EX_oaa(e)** | 0 | 0.63 | 0.63 | 0 | 0.77 | 0.77 | 0 | 0.86 | 0.86 |
| **EX_orn(e)** | 0 | 0.27 | 0.27 | 0 | 0.31 | 0.31 | 0 | 0.36 | 0.36 |
| **EX_pacald(e)** | 0 | 0.17 | 0.17 | 0 | 0.19 | 0.19 | 0 | 0.24 | 0.24 |
| **EX_pap(e)** | 0 | 0.06 | 0.06 | 0 | 0.07 | 0.07 | 0 | 0.09 | 0.09 |
| **EX_phe-L(e)** | 0 | 0.17 | 0.17 | 0 | 0.19 | 0.19 | 0 | 0.24 | 0.24 |
| **EX_pheac(e)** | 0 | 0.14 | 0.14 | 0 | 0.15 | 0.15 | 0 | 0.19 | 0.19 |
| **EX_pi(e)** | -0.33 | -0.02 | 0.31 | -0.37 | -0.03 | 0.34 | -0.47 | -0.03 | 0.44 |
| **EX_pnto-R(e)** | 0 | 0.09 | 0.09 | 0 | 0.1 | 0.1 | 0 | 0.13 | 0.13 |
| **EX_pro-L(e)** | 0 | 0.3 | 0.3 | 0 | 0.35 | 0.35 | 0 | 0.41 | 0.41 |
| **EX_ptrc(e)** | 0 | 0.27 | 0.27 | 0 | 0.31 | 0.31 | 0 | 0.36 | 0.36 |
| **EX_sbt-D(e)** | 0 | 0.57 | 0.57 | 0 | 0.63 | 0.63 | 0 | 0.8 | 0.8 |
| **EX_ser-L(e)** | 0 | 0.42 | 0.42 | 0 | 0.51 | 0.51 | 0 | 0.58 | 0.58 |
| **EX_so3(e)** | 0 | 0.57 | 0.57 | 0 | 0.63 | 0.63 | 0 | 0.8 | 0.8 |
| **EX_so4(e)** | -0.58 | -0.01 | 0.57 | -0.64 | -0.01 | 0.63 | -0.81 | -0.01 | 0.8 |
| **EX_spmd(e)** | 0 | 0.12 | 0.12 | 0 | 0.13 | 0.13 | 0 | 0.17 | 0.17 |
| **EX_thr-L(e)** | 0 | 0.37 | 0.37 | 0 | 0.41 | 0.41 | 0 | 0.52 | 0.52 |
| **EX_thym(e)** | 0 | 0.11 | 0.11 | 0 | 0.13 | 0.13 | 0 | 0.16 | 0.16 |
| **EX_trp-L(e)** | 0 | 0.1 | 0.1 | 0 | 0.11 | 0.11 | 0 | 0.13 | 0.13 |
| **EX_ttdca(e)** | 0 | 0.05 | 0.05 | 0 | 0.06 | 0.06 | 0 | 0.07 | 0.07 |
| **EX_tyr-L(e)** | 0 | 0.17 | 0.17 | 0 | 0.19 | 0.19 | 0 | 0.24 | 0.24 |
| **EX_urea(e)** | 0 | 0.2 | 0.2 | 0 | 0.23 | 0.23 | 0 | 0.29 | 0.29 |
| **EX_val-L(e)** | 0 | 0.61 | 0.61 | 0 | 0.71 | 0.71 | 0 | 0.86 | 0.86 |
| **EX_xan(e)** | 0 | 0.08 | 0.08 | 0 | 0.1 | 0.1 | 0 | 0.12 | 0.12 |
| **13BGH** | 0 | 0.61 | 0.61 | 0 | 0.68 | 0.68 | 0 | 0.86 | 0.86 |
| **13GS** | 0.14 | 0.75 | 0.61 | 0.15 | 0.83 | 0.68 | 0.19 | 1.06 | 0.87 |
| **2DDA7Ptm** | -0.23 | 0 | 0.23 | -0.25 | 0 | 0.25 | -0.32 | 0 | 0.32 |
| **2DHPtm** | 0 | 0.09 | 0.09 | 0 | 0.1 | 0.1 | 0 | 0.13 | 0.13 |
| **2HBO** | -0.49 | 0 | 0.49 | -0.52 | 0 | 0.52 | -0.69 | 0 | 0.69 |
| **2HBt2** | -0.49 | 0 | 0.49 | -0.52 | 0 | 0.52 | -0.69 | 0 | 0.69 |
| **2MBACt** | 0 | 0.21 | 0.21 | 0 | 0.23 | 0.23 | 0 | 0.29 | 0.29 |
| **2MBALDt** | 0 | 0.3 | 0.3 | 0 | 0.33 | 0.33 | 0 | 0.42 | 0.42 |
| **2MBALDtm** | 0 | 0.38 | 0.38 | 0 | 0.41 | 0.41 | 0 | 0.53 | 0.53 |
| **2MBTOHt** | 0 | 0.38 | 0.38 | 0 | 0.41 | 0.41 | 0 | 0.53 | 0.53 |
| **2MBTOHtm** | -0.38 | 0 | 0.38 | -0.41 | 0 | 0.41 | -0.53 | 0 | 0.53 |
| **2MPPALt** | 0 | 0.63 | 0.63 | 0 | 0.77 | 0.77 | 0 | 0.86 | 0.86 |
| **2MPPALtm** | 0 | 1.23 | 1.23 | 0 | 1.35 | 1.35 | 0 | 1.73 | 1.73 |
| **2OBUTtm** | -0.44 | 0.4 | 0.84 | -0.48 | 0.43 | 0.91 | -0.62 | 0.56 | 1.18 |
| **2OXOADPtim** | 0.04 | 0.17 | 0.13 | 0.04 | 0.21 | 0.17 | 0.05 | 0.24 | 0.19 |
| **2PHETOHt** | -0.19 | 0 | 0.19 | -0.22 | 0 | 0.22 | -0.27 | 0 | 0.27 |
| **2PHETOHtm** | 0 | 0.19 | 0.19 | 0 | 0.22 | 0.22 | 0 | 0.27 | 0.27 |
| **34HPPt2m** | 0 | 0.18 | 0.18 | 0 | 0.21 | 0.21 | 0 | 0.25 | 0.25 |
| **3C3HMPt** | 0 | 0.3 | 0.3 | 0 | 0.39 | 0.39 | 0 | 0.41 | 0.41 |
| **3C3HMPtm** | -0.33 | 0 | 0.33 | -0.43 | 0 | 0.43 | -0.46 | 0 | 0.46 |
| **3MBALDt** | 0 | 0.24 | 0.24 | 0 | 0.32 | 0.32 | 0 | 0.33 | 0.33 |
| **3MBALDtm** | 0 | 0.3 | 0.3 | 0 | 0.39 | 0.39 | 0 | 0.41 | 0.41 |
| **3MOBDC** | 0 | 1.23 | 1.23 | 0 | 1.35 | 1.35 | 0 | 1.73 | 1.73 |
| **3MOBtm** | -3.16 | -0.03 | 3.13 | -3.28 | -0.04 | 3.24 | -3.62 | -0.05 | 3.57 |
| **3MOPDC** | 0 | 0.38 | 0.38 | 0 | 0.41 | 0.41 | 0 | 0.53 | 0.53 |
| **3MOPt** | -0.3 | 0 | 0.3 | -0.33 | 0 | 0.33 | -0.42 | 0 | 0.42 |
| **3MOPtm** | -0.4 | 499.98 | 500.38 | -0.43 | 499.97 | 500.4 | -0.56 | 499.97 | 500.53 |
| **4ABZt** | 0 | 0.17 | 0.17 | 0 | 0.19 | 0.19 | 0 | 0.24 | 0.24 |
| **4MOPDC** | 0 | 0.3 | 0.3 | 0 | 0.39 | 0.39 | 0 | 0.41 | 0.41 |
| **AABTN** | 0 | 0.05 | 0.05 | 0 | 0.06 | 0.06 | 0 | 0.07 | 0.07 |
| **AASAD1** | 0 | 0.15 | 0.15 | 0 | 0.19 | 0.19 | 0 | 0.21 | 0.21 |
| **AASAD2** | 0 | 0.17 | 0.17 | 0 | 0.21 | 0.21 | 0 | 0.24 | 0.24 |
| **AATA** | 0.04 | 0.17 | 0.13 | 0.04 | 0.21 | 0.17 | 0.05 | 0.24 | 0.19 |
| **ABUTt2r** | -0.3 | 0 | 0.3 | -0.39 | 0 | 0.39 | -0.41 | 0 | 0.41 |
| **ACACT1** | 0 | 0.1 | 0.1 | 0 | 0.12 | 0.12 | 0 | 0.14 | 0.14 |
| **ACACT1m** | 0 | 0.13 | 0.13 | 0 | 0.16 | 0.16 | 0 | 0.17 | 0.17 |
| **ACCOACr** | 0.05 | 0.35 | 0.3 | 0.05 | 0.39 | 0.34 | 0.07 | 0.48 | 0.41 |
| **ACESt** | 0 | 0.42 | 0.42 | 0 | 0.52 | 0.52 | 0 | 0.58 | 0.58 |
| **ACGKm** | 0.02 | 0.65 | 0.63 | 0.02 | 0.71 | 0.69 | 0.03 | 0.82 | 0.79 |
| **ACHBSm** | 0.02 | 0.4 | 0.38 | 0.03 | 0.43 | 0.4 | 0.03 | 0.56 | 0.53 |
| **ACHLE1** | 0 | 0.61 | 0.61 | 0 | 0.68 | 0.68 | 0 | 0.86 | 0.86 |
| **ACHLE2** | 0 | 0.61 | 0.61 | 0 | 0.68 | 0.68 | 0 | 0.86 | 0.86 |
| **ACHLE3** | 0 | 0.61 | 0.61 | 0 | 0.68 | 0.68 | 0 | 0.86 | 0.86 |
| **ACLSm** | 0.07 | 1.3 | 1.23 | 0.08 | 1.43 | 1.35 | 0.1 | 1.82 | 1.72 |
| **ACOAHim** | 0 | 0.71 | 0.71 | 0 | 0.84 | 0.84 | 0 | 0.91 | 0.91 |
| **ACONT3m** | 0 | 0.26 | 0.26 | 0 | 0.3 | 0.3 | 0 | 0.36 | 0.36 |
| **ACOTAim** | 0.02 | 0.65 | 0.63 | 0.02 | 0.71 | 0.69 | 0.03 | 0.82 | 0.79 |
| **ACPSm** | 0 | 0.35 | 0.35 | 0 | 0.39 | 0.39 | 0 | 0.49 | 0.49 |
| **ACRNtm** | 0 | 0.66 | 0.66 | 0 | 0.72 | 0.72 | 0 | 0.92 | 0.92 |
| **ACS** | 0.06 | 0.79 | 0.73 | 0.07 | 0.87 | 0.8 | 0.09 | 1.11 | 1.02 |
| **ACSm** | 0 | 0.72 | 0.72 | 0 | 0.76 | 0.76 | 0 | 0.91 | 0.91 |
| **ACtm** | -0.63 | 0.52 | 1.15 | -0.83 | 0.63 | 1.46 | -0.86 | 0.66 | 1.52 |
| **ADA** | 0 | 0.41 | 0.41 | 0 | 0.47 | 0.47 | 0 | 0.58 | 0.58 |
| **ADCL** | 0 | 0.17 | 0.17 | 0 | 0.19 | 0.19 | 0 | 0.24 | 0.24 |
| **ADCS** | 0 | 0.17 | 0.17 | 0 | 0.19 | 0.19 | 0 | 0.24 | 0.24 |
| **ADD** | 0 | 0.41 | 0.41 | 0 | 0.47 | 0.47 | 0 | 0.58 | 0.58 |
| **ADHAPR_SC** | 0 | 0.12 | 0.12 | 0 | 0.13 | 0.13 | 0 | 0.17 | 0.17 |
| **ADK1m** | -1.58 | 0.58 | 2.16 | -1.63 | 0.67 | 2.3 | -1.81 | 0.68 | 2.49 |
| **ADK3m** | 0 | 1.78 | 1.78 | 0 | 1.85 | 1.85 | 0 | 2.09 | 2.09 |
| **ADMDC** | 0 | 0.12 | 0.12 | 0 | 0.13 | 0.13 | 0 | 0.17 | 0.17 |
| **ADNCYC** | 0 | 0.61 | 0.61 | 0 | 0.68 | 0.68 | 0 | 0.86 | 0.86 |
| **ADNK1** | 0 | 1.23 | 1.23 | 0 | 1.36 | 1.36 | 0 | 1.73 | 1.73 |
| **ADNUC** | 0 | 1.23 | 1.23 | 0 | 1.35 | 1.35 | 0 | 1.73 | 1.73 |
| **ADPT** | 0 | 0.62 | 0.62 | 0 | 0.68 | 0.68 | 0 | 0.86 | 0.86 |
| **ADSK** | 0.01 | 0.57 | 0.56 | 0.01 | 0.63 | 0.62 | 0.01 | 0.81 | 0.8 |
| **ADSL1r** | 0.01 | 0.63 | 0.62 | 0.02 | 0.72 | 0.7 | 0.02 | 0.88 | 0.86 |
| **ADSL2r** | 0.01 | 0.1 | 0.09 | 0.01 | 0.12 | 0.11 | 0.02 | 0.14 | 0.12 |
| **ADSS** | 0.01 | 0.63 | 0.62 | 0.02 | 0.72 | 0.7 | 0.02 | 0.88 | 0.86 |
| **AGAT_SC** | 0 | 0.13 | 0.13 | 0 | 0.14 | 0.14 | 0 | 0.18 | 0.18 |
| **AGPRim** | 0.02 | 0.65 | 0.63 | 0.02 | 0.71 | 0.69 | 0.03 | 0.82 | 0.79 |
| **AGTi** | 0 | 0.33 | 0.33 | 0 | 0.43 | 0.43 | 0 | 0.46 | 0.46 |
| **AHCi** | 0 | 0.41 | 0.41 | 0 | 0.45 | 0.45 | 0 | 0.58 | 0.58 |
| **AHSERL2** | 0 | 0.15 | 0.15 | 0 | 0.17 | 0.17 | 0 | 0.21 | 0.21 |
| **AICART** | 0.02 | 0.11 | 0.09 | 0.02 | 0.13 | 0.11 | 0.03 | 0.16 | 0.13 |
| **AIRCr** | 0.01 | 0.1 | 0.09 | 0.01 | 0.12 | 0.11 | 0.02 | 0.14 | 0.12 |
| **ALATA_Lm** | -3.1 | 0 | 3.1 | -3.2 | 0 | 3.2 | -3.53 | 0 | 3.53 |
| **ALAt2r** | -1.23 | 0 | 1.23 | -1.45 | 0 | 1.45 | -1.73 | 0 | 1.73 |
| **ALAtmi** | 0 | 3.1 | 3.1 | 0 | 3.2 | 3.2 | 0 | 3.53 | 3.53 |
| **ALCD22xi** | 0 | 0.38 | 0.38 | 0 | 0.41 | 0.41 | 0 | 0.53 | 0.53 |
| **ALCD22xim** | 0 | 0.38 | 0.38 | 0 | 0.41 | 0.41 | 0 | 0.53 | 0.53 |
| **ALCD22yi** | 0 | 0.29 | 0.29 | 0 | 0.32 | 0.32 | 0 | 0.41 | 0.41 |
| **ALCD23xi** | 0 | 1.23 | 1.23 | 0 | 1.35 | 1.35 | 0 | 1.73 | 1.73 |
| **ALCD23xim** | 0 | 1.23 | 1.23 | 0 | 1.35 | 1.35 | 0 | 1.73 | 1.73 |
| **ALCD23yi** | 0 | 0.61 | 0.61 | 0 | 0.71 | 0.71 | 0 | 0.86 | 0.86 |
| **ALCD24xi** | 0 | 0.3 | 0.3 | 0 | 0.39 | 0.39 | 0 | 0.41 | 0.41 |
| **ALCD24xim** | 0 | 0.3 | 0.3 | 0 | 0.39 | 0.39 | 0 | 0.41 | 0.41 |
| **ALCD24yi** | 0 | 0.24 | 0.24 | 0 | 0.3 | 0.3 | 0 | 0.33 | 0.33 |
| **ALCD25xi** | 0 | 0.19 | 0.19 | 0 | 0.22 | 0.22 | 0 | 0.27 | 0.27 |
| **ALCD25xim** | 0 | 0.19 | 0.19 | 0 | 0.22 | 0.22 | 0 | 0.27 | 0.27 |
| **ALCD25yi** | 0 | 0.17 | 0.17 | 0 | 0.19 | 0.19 | 0 | 0.24 | 0.24 |
| **ALCD26xi** | 0 | 0.1 | 0.1 | 0 | 0.12 | 0.12 | 0 | 0.14 | 0.14 |
| **ALCD26xim** | 0 | 0.1 | 0.1 | 0 | 0.12 | 0.12 | 0 | 0.14 | 0.14 |
| **ALDD22x** | 0 | 0.09 | 0.09 | 0 | 0.1 | 0.1 | 0 | 0.13 | 0.13 |
| **ALDD2xm** | 0 | 0.65 | 0.65 | 0 | 0.83 | 0.83 | 0 | 0.9 | 0.9 |
| **ALDD2ym** | 0 | 0.64 | 0.64 | 0 | 0.78 | 0.78 | 0 | 0.84 | 0.84 |
| **ALPHNH** | 0 | 0.18 | 0.18 | 0 | 0.2 | 0.2 | 0 | 0.25 | 0.25 |
| **AMPDA** | 0 | 0.61 | 0.61 | 0 | 0.71 | 0.71 | 0 | 0.86 | 0.86 |
| **AMPN** | 0 | 1.23 | 1.23 | 0 | 1.35 | 1.35 | 0 | 1.73 | 1.73 |
| **ANPRT** | 0 | 0.14 | 0.14 | 0 | 0.17 | 0.17 | 0 | 0.2 | 0.2 |
| **ANS** | 0 | 0.11 | 0.11 | 0 | 0.12 | 0.12 | 0 | 0.15 | 0.15 |
| **AP4AHr** | 0 | 1.23 | 1.23 | 0 | 1.35 | 1.35 | 0 | 1.73 | 1.73 |
| **APRTO2** | 0 | 0.05 | 0.05 | 0 | 0.06 | 0.06 | 0 | 0.07 | 0.07 |
| **ARGN** | 0 | 0.2 | 0.2 | 0 | 0.23 | 0.23 | 0 | 0.29 | 0.29 |
| **ARGSL** | 0.02 | 0.22 | 0.2 | 0.02 | 0.25 | 0.23 | 0.03 | 0.32 | 0.29 |
| **ARGSSr** | 0.02 | 0.22 | 0.2 | 0.02 | 0.25 | 0.23 | 0.03 | 0.32 | 0.29 |
| **ARGt2r** | -0.12 | 0 | 0.12 | -0.13 | 0 | 0.13 | -0.16 | 0 | 0.16 |
| **ASADi** | 0.05 | 0.59 | 0.54 | 0.06 | 0.65 | 0.59 | 0.07 | 0.85 | 0.78 |
| **ASNN** | 0 | 0.41 | 0.41 | 0 | 0.45 | 0.45 | 0 | 0.58 | 0.58 |
| **ASNNe** | 0 | 0.41 | 0.41 | 0 | 0.45 | 0.45 | 0 | 0.58 | 0.58 |
| **ASNS1** | 0.01 | 0.42 | 0.41 | 0.01 | 0.47 | 0.46 | 0.02 | 0.59 | 0.57 |
| **ASNt2r** | -0.41 | 0 | 0.41 | -0.45 | 0 | 0.45 | -0.58 | 0 | 0.58 |
| **ASNt6** | 0 | 0.2 | 0.2 | 0 | 0.23 | 0.23 | 0 | 0.29 | 0.29 |
| **ASNt7** | 0 | 0.2 | 0.2 | 0 | 0.23 | 0.23 | 0 | 0.29 | 0.29 |
| **ASPCT** | 0.01 | 0.13 | 0.12 | 0.02 | 0.14 | 0.12 | 0.02 | 0.18 | 0.16 |
| **ASPGLU2m** | 0 | 0.35 | 0.35 | 0 | 0.47 | 0.47 | 0 | 0.48 | 0.48 |
| **ASPKi** | 0.05 | 0.59 | 0.54 | 0.06 | 0.65 | 0.59 | 0.07 | 0.85 | 0.78 |
| **ASPTA** | -500 | 2.94 | 502.94 | -500 | 3.03 | 503.03 | -500 | 3.31 | 503.31 |
| **ASPTAm** | -3.11 | 499.84 | 502.95 | -3.22 | 499.82 | 503.04 | -3.55 | 499.77 | 503.32 |
| **ASPt2m** | -3.11 | 500 | 503.11 | -3.22 | 500 | 503.22 | -3.55 | 500 | 503.55 |
| **ASPt2r** | -0.61 | 0.41 | 1.02 | -0.71 | 0.45 | 1.16 | -0.86 | 0.58 | 1.44 |
| **ATPATF1** | 0 | 1.23 | 1.23 | 0 | 1.35 | 1.35 | 0 | 1.73 | 1.73 |
| **ATPH1** | 0 | 0.61 | 0.61 | 0 | 0.68 | 0.68 | 0 | 0.86 | 0.86 |
| **ATPPRT** | 0.01 | 0.1 | 0.09 | 0.01 | 0.12 | 0.11 | 0.01 | 0.14 | 0.13 |
| **ATPS** | 0 | 1.23 | 1.23 | 0 | 1.35 | 1.35 | 0 | 1.73 | 1.73 |
| **ATPS3m** | 0 | 1.19 | 1.19 | 0 | 1.23 | 1.23 | 0 | 1.39 | 1.39 |
| **ATPtm-H** | 0 | 1.55 | 1.55 | 0 | 1.6 | 1.6 | 0 | 1.76 | 1.76 |
| **BPNT** | 0 | 0.57 | 0.57 | 0 | 0.63 | 0.63 | 0 | 0.81 | 0.81 |
| **CAT** | 0 | 0.06 | 0.06 | 0 | 0.07 | 0.07 | 0 | 0.08 | 0.08 |
| **CBPS** | 0.03 | 0.24 | 0.21 | 0.04 | 0.27 | 0.23 | 0.05 | 0.33 | 0.28 |
| **CHLPCTD** | 0 | 0.41 | 0.41 | 0 | 0.45 | 0.45 | 0 | 0.58 | 0.58 |
| **CHOLK** | 0 | 0.41 | 0.41 | 0 | 0.45 | 0.45 | 0 | 0.58 | 0.58 |
| **CHORM** | 0.03 | 0.22 | 0.19 | 0.03 | 0.25 | 0.22 | 0.04 | 0.31 | 0.27 |
| **CHORS** | 0.03 | 0.23 | 0.2 | 0.04 | 0.25 | 0.21 | 0.05 | 0.32 | 0.27 |
| **CITt2r** | -0.3 | 0 | 0.3 | -0.39 | 0 | 0.39 | -0.41 | 0 | 0.41 |
| **CITtam** | -0.46 | 0.17 | 0.63 | -0.59 | 0.19 | 0.78 | -0.63 | 0.24 | 0.87 |
| **CITtbm** | -0.23 | 0 | 0.23 | -0.25 | 0 | 0.25 | -0.32 | 0 | 0.32 |
| **CMPN** | 0 | 0.61 | 0.61 | 0 | 0.68 | 0.68 | 0 | 0.86 | 0.86 |
| **CO2tv** | -0.04 | 0 | 0.04 | -0.05 | 0 | 0.05 | -0.06 | 0 | 0.06 |
| **COAtim** | 0 | 0.35 | 0.35 | 0 | 0.39 | 0.39 | 0 | 0.5 | 0.5 |
| **CRNCARtm** | 0 | 0.66 | 0.66 | 0 | 0.72 | 0.72 | 0 | 0.92 | 0.92 |
| **CRNtim** | 0 | 0.66 | 0.66 | 0 | 0.72 | 0.72 | 0 | 0.92 | 0.92 |
| **CSNATm** | 0 | 0.66 | 0.66 | 0 | 0.72 | 0.72 | 0 | 0.92 | 0.92 |
| **CSNATr** | 0 | 0.66 | 0.66 | 0 | 0.72 | 0.72 | 0 | 0.92 | 0.92 |
| **CSND** | 0 | 0.61 | 0.61 | 0 | 0.68 | 0.68 | 0 | 0.86 | 0.86 |
| **CTPS1** | 0 | 0.62 | 0.62 | 0 | 0.68 | 0.68 | 0 | 0.87 | 0.87 |
| **CTPS2** | 0 | 0.41 | 0.41 | 0 | 0.46 | 0.46 | 0 | 0.58 | 0.58 |
| **CTPtm** | 0 | 1.15 | 1.15 | 0 | 1.26 | 1.26 | 0 | 1.42 | 1.42 |
| **CYSS** | 0 | 0.17 | 0.17 | 0 | 0.19 | 0.19 | 0 | 0.24 | 0.24 |
| **CYSTL** | 0 | 0.61 | 0.61 | 0 | 0.71 | 0.71 | 0 | 0.86 | 0.86 |
| **CYSTS** | 0 | 0.62 | 0.62 | 0 | 0.71 | 0.71 | 0 | 0.86 | 0.86 |
| **CYSt2r** | -0.17 | 0 | 0.17 | -0.19 | 0 | 0.19 | -0.23 | 0 | 0.23 |
| **CYTD** | 0 | 0.61 | 0.61 | 0 | 0.68 | 0.68 | 0 | 0.86 | 0.86 |
| **CYTDK2** | 0 | 1.23 | 1.23 | 0 | 1.35 | 1.35 | 0 | 1.73 | 1.73 |
| **CYTK1** | -0.62 | 0.49 | 1.11 | -0.68 | 0.54 | 1.22 | -0.87 | 0.69 | 1.56 |
| **CYTK2** | -0.08 | 500 | 500.08 | -0.09 | 500 | 500.09 | -0.11 | 500 | 500.11 |
| **D-LACDcm** | 0 | 0.52 | 0.52 | 0 | 0.57 | 0.57 | 0 | 0.58 | 0.58 |
| **D-LACDm** | 0 | 0.52 | 0.52 | 0 | 0.57 | 0.57 | 0 | 0.58 | 0.58 |
| **D-LACt2** | -0.61 | 0 | 0.61 | -0.68 | 0 | 0.68 | -0.86 | 0 | 0.86 |
| **DADA** | 0 | 0.41 | 0.41 | 0 | 0.47 | 0.47 | 0 | 0.58 | 0.58 |
| **DADK** | -0.08 | 0 | 0.08 | -0.08 | 0 | 0.08 | -0.11 | 0 | 0.11 |
| **DAGCPT_SC** | 0 | 0.41 | 0.41 | 0 | 0.45 | 0.45 | 0 | 0.58 | 0.58 |
| **DAGPYP_SC** | 0 | 0.41 | 0.41 | 0 | 0.45 | 0.45 | 0 | 0.58 | 0.58 |
| **DASYN_SC** | -1.15 | 0.25 | 1.4 | -1.26 | 0.27 | 1.53 | -1.42 | 0.35 | 1.77 |
| **DASYNm_SC** | 0 | 1.15 | 1.15 | 0 | 1.26 | 1.26 | 0 | 1.42 | 1.42 |
| **DCMPDA** | -500 | 0.08 | 500.08 | -500 | 0.09 | 500.09 | -500 | 0.11 | 500.11 |
| **DCYTD** | 0 | 1.23 | 1.23 | 0 | 1.35 | 1.35 | 0 | 1.73 | 1.73 |
| **DDPA** | 0 | 0.23 | 0.23 | 0 | 0.25 | 0.25 | 0 | 0.32 | 0.32 |
| **DDPAm** | 0 | 0.23 | 0.23 | 0 | 0.25 | 0.25 | 0 | 0.32 | 0.32 |
| **DGK1** | -0.08 | 1.23 | 1.31 | -0.08 | 1.35 | 1.43 | -0.11 | 1.72 | 1.83 |
| **DHAD1m** | 0.07 | 1.3 | 1.23 | 0.08 | 1.43 | 1.35 | 0.1 | 1.82 | 1.72 |
| **DHAD2m** | 0.02 | 0.4 | 0.38 | 0.03 | 0.43 | 0.4 | 0.03 | 0.56 | 0.53 |
| **DHFRi** | 0 | 0.11 | 0.11 | 0 | 0.13 | 0.13 | 0 | 0.16 | 0.16 |
| **DHORD4i** | 0 | 0.13 | 0.13 | 0 | 0.14 | 0.14 | 0 | 0.18 | 0.18 |
| **DHORDfum** | 0 | 0.13 | 0.13 | 0 | 0.14 | 0.14 | 0 | 0.18 | 0.18 |
| **DHORDi** | 0 | 0.12 | 0.12 | 0 | 0.13 | 0.13 | 0 | 0.16 | 0.16 |
| **DHORTS** | -0.13 | -0.01 | 0.12 | -0.14 | -0.02 | 0.12 | -0.18 | -0.02 | 0.16 |
| **DHQS** | 0.03 | 0.23 | 0.2 | 0.04 | 0.25 | 0.21 | 0.05 | 0.32 | 0.27 |
| **DHQTi** | 0.03 | 0.23 | 0.2 | 0.04 | 0.25 | 0.21 | 0.05 | 0.32 | 0.27 |
| **DKMPPD2** | 0 | 0.12 | 0.12 | 0 | 0.13 | 0.13 | 0 | 0.17 | 0.17 |
| **DPCOAK** | 0 | 0.35 | 0.35 | 0 | 0.39 | 0.39 | 0 | 0.49 | 0.49 |
| **DPMVD** | 0 | 0.13 | 0.13 | 0 | 0.16 | 0.16 | 0 | 0.17 | 0.17 |
| **DPR** | 0 | 0.09 | 0.09 | 0 | 0.1 | 0.1 | 0 | 0.13 | 0.13 |
| **DPRm** | 0 | 0.09 | 0.09 | 0 | 0.1 | 0.1 | 0 | 0.13 | 0.13 |
| **DTMPK** | 0 | 0.08 | 0.08 | 0 | 0.1 | 0.1 | 0 | 0.12 | 0.12 |
| **DTTPt** | -0.08 | 0 | 0.08 | -0.1 | 0 | 0.1 | -0.12 | 0 | 0.12 |
| **DURIK1** | 0 | 1.23 | 1.23 | 0 | 1.35 | 1.35 | 0 | 1.73 | 1.73 |
| **DURIPP** | -0.12 | 0 | 0.12 | -0.13 | 0 | 0.13 | -0.16 | 0 | 0.16 |
| **DUTPDP** | 0 | 0.61 | 0.61 | 0 | 0.68 | 0.68 | 0 | 0.86 | 0.86 |
| **E4Ptm** | 0 | 0.23 | 0.23 | 0 | 0.25 | 0.25 | 0 | 0.32 | 0.32 |
| **FACOAL140** | -0.06 | 0.04 | 0.1 | -0.06 | 0.05 | 0.11 | -0.08 | 0.06 | 0.14 |
| **FACOAL160** | 0 | 0.1 | 0.1 | 0 | 0.11 | 0.11 | 0 | 0.14 | 0.14 |
| **FACOAL161** | 0 | 0.06 | 0.06 | 0 | 0.07 | 0.07 | 0 | 0.08 | 0.08 |
| **FACOAL181** | 0 | 0.09 | 0.09 | 0 | 0.1 | 0.1 | 0 | 0.12 | 0.12 |
| **FAS100** | 0 | 0.05 | 0.05 | 0 | 0.06 | 0.06 | 0 | 0.07 | 0.07 |
| **FAS100COA** | 0 | 0.06 | 0.06 | 0 | 0.06 | 0.06 | 0 | 0.08 | 0.08 |
| **FAS120** | 0 | 0.05 | 0.05 | 0 | 0.06 | 0.06 | 0 | 0.07 | 0.07 |
| **FAS120COA** | 0 | 0.06 | 0.06 | 0 | 0.06 | 0.06 | 0 | 0.08 | 0.08 |
| **FAS140** | 0 | 0.05 | 0.05 | 0 | 0.06 | 0.06 | 0 | 0.07 | 0.07 |
| **FAS140COA** | 0 | 0.06 | 0.06 | 0 | 0.06 | 0.06 | 0 | 0.08 | 0.08 |
| **FAS80COA_L** | 0 | 0.06 | 0.06 | 0 | 0.06 | 0.06 | 0 | 0.08 | 0.08 |
| **FAS80_L** | 0 | 0.05 | 0.05 | 0 | 0.06 | 0.06 | 0 | 0.07 | 0.07 |
| **FBA** | -1.23 | 5.72 | 6.95 | -1.35 | 7.63 | 8.98 | -1.73 | 7.53 | 9.26 |
| **FBP** | 0 | 1.23 | 1.23 | 0 | 1.35 | 1.35 | 0 | 1.73 | 1.73 |
| **FBP26** | 0 | 1.23 | 1.23 | 0 | 1.35 | 1.35 | 0 | 1.73 | 1.73 |
| **FDH** | 0 | 0.13 | 0.13 | 0 | 0.15 | 0.15 | 0 | 0.18 | 0.18 |
| **FDNG** | 0 | 0.13 | 0.13 | 0 | 0.15 | 0.15 | 0 | 0.18 | 0.18 |
| **FKYNH** | 0 | 0.14 | 0.14 | 0 | 0.17 | 0.17 | 0 | 0.19 | 0.19 |
| **FORt** | -0.14 | 0 | 0.14 | -0.17 | 0 | 0.17 | -0.19 | 0 | 0.19 |
| **FTHFCLm** | 0 | 1.23 | 1.23 | 0 | 1.31 | 1.31 | 0 | 1.54 | 1.54 |
| **FTHFLi** | 0 | 0.13 | 0.13 | 0 | 0.15 | 0.15 | 0 | 0.19 | 0.19 |
| **FUM** | -500 | 0.66 | 500.66 | -500 | 0.76 | 500.76 | -500 | 0.93 | 500.93 |
| **FUMt2r** | -1.23 | 0 | 1.23 | -1.35 | 0 | 1.35 | -1.73 | 0 | 1.73 |
| **G3PCt** | -0.05 | 0 | 0.05 | -0.05 | 0 | 0.05 | -0.07 | 0 | 0.07 |
| **G5SADr** | 0.02 | 0.65 | 0.63 | 0.02 | 0.72 | 0.7 | 0.03 | 0.83 | 0.8 |
| **G5SADrm** | -0.63 | 0 | 0.63 | -0.69 | 0 | 0.69 | -0.8 | 0 | 0.8 |
| **G5SD** | 0 | 0.28 | 0.28 | 0 | 0.33 | 0.33 | 0 | 0.39 | 0.39 |
| **G5SD2** | 0 | 0.35 | 0.35 | 0 | 0.41 | 0.41 | 0 | 0.48 | 0.48 |
| **G6PDA** | 0 | 1.23 | 1.23 | 0 | 1.35 | 1.35 | 0 | 1.73 | 1.73 |
| **GAM6Pt** | -0.31 | 0 | 0.31 | -0.34 | 0 | 0.34 | -0.43 | 0 | 0.43 |
| **GARFTi** | 0.01 | 0.1 | 0.09 | 0.01 | 0.12 | 0.11 | 0.02 | 0.14 | 0.12 |
| **GAT1_SC** | 0 | 0.13 | 0.13 | 0 | 0.14 | 0.14 | 0 | 0.18 | 0.18 |
| **GAT2_SC** | 0 | 0.12 | 0.12 | 0 | 0.13 | 0.13 | 0 | 0.17 | 0.17 |
| **GBEZ** | 0 | 1.29 | 1.29 | 0 | 1.42 | 1.42 | 0 | 1.81 | 1.81 |
| **GCC2am** | 0 | 0.27 | 0.27 | 0 | 0.29 | 0.29 | 0 | 0.38 | 0.38 |
| **GCC2bim** | 0 | 0.27 | 0.27 | 0 | 0.29 | 0.29 | 0 | 0.38 | 0.38 |
| **GCC2cm** | -500 | 0.34 | 500.34 | -500 | 0.45 | 500.45 | -500 | 0.47 | 500.47 |
| **GCCam** | 0 | 0.27 | 0.27 | 0 | 0.29 | 0.29 | 0 | 0.38 | 0.38 |
| **GCCbim** | 0 | 0.27 | 0.27 | 0 | 0.29 | 0.29 | 0 | 0.38 | 0.38 |
| **GCCcm** | 0 | 0.27 | 0.27 | 0 | 0.29 | 0.29 | 0 | 0.38 | 0.38 |
| **GF6PTA** | 0 | 1.23 | 1.23 | 0 | 1.35 | 1.35 | 0 | 1.73 | 1.73 |
| **GHMT2r** | -0.09 | 0.22 | 0.31 | -0.1 | 0.26 | 0.36 | -0.13 | 0.31 | 0.44 |
| **GHMT2rm** | -0.27 | 0 | 0.27 | -0.29 | 0 | 0.29 | -0.38 | 0 | 0.38 |
| **GLCGSD** | 0 | 0.61 | 0.61 | 0 | 0.68 | 0.68 | 0 | 0.86 | 0.86 |
| **GLCGSDv** | 0 | 0.61 | 0.61 | 0 | 0.68 | 0.68 | 0 | 0.86 | 0.86 |
| **GLCNtv** | 0 | 0.61 | 0.61 | 0 | 0.68 | 0.68 | 0 | 0.86 | 0.86 |
| **GLCP** | 0 | 1.23 | 1.23 | 0 | 1.35 | 1.35 | 0 | 1.73 | 1.73 |
| **GLCS2** | 0 | 1.29 | 1.29 | 0 | 1.42 | 1.42 | 0 | 1.81 | 1.81 |
| **GLCt1** | 5.45 | 6.06 | 0.61 | 5.38 | 6.06 | 0.68 | 5.2 | 6.06 | 0.86 |
| **GLCtv** | -0.82 | 0 | 0.82 | -0.9 | 0 | 0.9 | -1.15 | 0 | 1.15 |
| **GLNt2r** | -0.24 | 0 | 0.24 | -0.3 | 0 | 0.3 | -0.33 | 0 | 0.33 |
| **GLNt6** | 0 | 0.2 | 0.2 | 0 | 0.23 | 0.23 | 0 | 0.29 | 0.29 |
| **GLNt7** | 0 | 0.2 | 0.2 | 0 | 0.23 | 0.23 | 0 | 0.29 | 0.29 |
| **GLU5K** | 0 | 0.35 | 0.35 | 0 | 0.41 | 0.41 | 0 | 0.48 | 0.48 |
| **GLUDxi** | 0 | 1.23 | 1.23 | 0 | 1.45 | 1.45 | 0 | 1.73 | 1.73 |
| **GLUDyi** | 0 | 1.83 | 1.83 | 0 | 2.12 | 2.12 | 0 | 2.57 | 2.57 |
| **GLUK** | 0 | 6.88 | 6.88 | 0 | 6.96 | 6.96 | 0 | 7.21 | 7.21 |
| **GLUN** | 0 | 1.23 | 1.23 | 0 | 1.35 | 1.35 | 0 | 1.73 | 1.73 |
| **GLUPRT** | 0.01 | 0.1 | 0.09 | 0.01 | 0.12 | 0.11 | 0.02 | 0.14 | 0.12 |
| **GLUt2r** | -0.3 | 0 | 0.3 | -0.39 | 0 | 0.39 | -0.41 | 0 | 0.41 |
| **GLUt5m** | 0 | 0.35 | 0.35 | 0 | 0.47 | 0.47 | 0 | 0.48 | 0.48 |
| **GLUt7m** | 0 | 0.37 | 0.37 | 0 | 0.49 | 0.49 | 0 | 0.51 | 0.51 |
| **GLYCLm** | 0 | 0.27 | 0.27 | 0 | 0.29 | 0.29 | 0 | 0.38 | 0.38 |
| **GLYGS** | 0 | 1.29 | 1.29 | 0 | 1.42 | 1.42 | 0 | 1.81 | 1.81 |
| **GLYK** | 0 | 1.23 | 1.23 | 0 | 1.35 | 1.35 | 0 | 1.73 | 1.73 |
| **GLYOX** | 0 | 0.61 | 0.61 | 0 | 0.68 | 0.68 | 0 | 0.86 | 0.86 |
| **GLYt2m** | 0 | 0.54 | 0.54 | 0 | 0.58 | 0.58 | 0 | 0.77 | 0.77 |
| **GLYt2r** | -0.49 | 0 | 0.49 | -0.52 | 0 | 0.52 | -0.69 | 0 | 0.69 |
| **GMPS2** | 0.01 | 0.25 | 0.24 | 0.01 | 0.28 | 0.27 | 0.01 | 0.35 | 0.34 |
| **GNNUC** | 0 | 1.23 | 1.23 | 0 | 1.35 | 1.35 | 0 | 1.73 | 1.73 |
| **GPDDA1** | 0 | 0.13 | 0.13 | 0 | 0.14 | 0.14 | 0 | 0.18 | 0.18 |
| **GSNK** | 0 | 1.23 | 1.23 | 0 | 1.35 | 1.35 | 0 | 1.73 | 1.73 |
| **GTHO** | 0 | 0.1 | 0.1 | 0 | 0.12 | 0.12 | 0 | 0.14 | 0.14 |
| **GTHP** | 0 | 0.1 | 0.1 | 0 | 0.12 | 0.12 | 0 | 0.14 | 0.14 |
| **GTPH1** | 0 | 0.61 | 0.61 | 0 | 0.68 | 0.68 | 0 | 0.86 | 0.86 |
| **GTPt2m** | 0 | 1.78 | 1.78 | 0 | 1.85 | 1.85 | 0 | 2.09 | 2.09 |
| **GUAD** | 0 | 0.25 | 0.25 | 0 | 0.27 | 0.27 | 0 | 0.35 | 0.35 |
| **GUAPRT** | 0 | 0.61 | 0.61 | 0 | 0.68 | 0.68 | 0 | 0.86 | 0.86 |
| **GUAt2r** | -0.07 | 0 | 0.07 | -0.08 | 0 | 0.08 | -0.1 | 0 | 0.1 |
| **H2Otm** | -2.36 | 500 | 502.36 | -2.47 | 500 | 502.47 | -2.8 | 500 | 502.8 |
| **H2Otv** | 0 | 0.61 | 0.61 | 0 | 0.68 | 0.68 | 0 | 0.86 | 0.86 |
| **HACNHm** | 0.04 | 0.17 | 0.13 | 0.04 | 0.21 | 0.17 | 0.05 | 0.24 | 0.19 |
| **HCITSm** | 0.04 | 0.17 | 0.13 | 0.04 | 0.21 | 0.17 | 0.05 | 0.24 | 0.19 |
| **HCYSMT** | 0 | 0.41 | 0.41 | 0 | 0.45 | 0.45 | 0 | 0.58 | 0.58 |
| **HETZK** | 0 | 0.41 | 0.41 | 0 | 0.45 | 0.45 | 0 | 0.58 | 0.58 |
| **HEX1** | 0 | 6.88 | 6.88 | 0 | 6.96 | 6.96 | 0 | 7.21 | 7.21 |
| **HEX7** | 0 | 1.23 | 1.23 | 0 | 1.45 | 1.45 | 0 | 1.73 | 1.73 |
| **HICITDm** | 0.04 | 0.17 | 0.13 | 0.04 | 0.21 | 0.17 | 0.05 | 0.24 | 0.19 |
| **HISTD** | 0.01 | 0.1 | 0.09 | 0.01 | 0.12 | 0.11 | 0.01 | 0.14 | 0.13 |
| **HISTP** | 0.01 | 0.1 | 0.09 | 0.01 | 0.12 | 0.11 | 0.01 | 0.14 | 0.13 |
| **HISt2r** | -0.09 | 0 | 0.09 | -0.11 | 0 | 0.11 | -0.13 | 0 | 0.13 |
| **HMGCOAR** | -0.13 | 0 | 0.13 | -0.16 | 0 | 0.16 | -0.17 | 0 | 0.17 |
| **HMGCOAS** | -0.1 | 0 | 0.1 | -0.12 | 0 | 0.12 | -0.14 | 0 | 0.14 |
| **HMGCOASm** | -0.13 | 0 | 0.13 | -0.16 | 0 | 0.16 | -0.17 | 0 | 0.17 |
| **HMGCOAtm** | -0.13 | 0 | 0.13 | -0.16 | 0 | 0.16 | -0.17 | 0 | 0.17 |
| **HMPK1** | 0 | 0.41 | 0.41 | 0 | 0.45 | 0.45 | 0 | 0.58 | 0.58 |
| **HSDxi** | 0 | 0.59 | 0.59 | 0 | 0.65 | 0.65 | 0 | 0.85 | 0.85 |
| **HSDyi** | 0 | 0.43 | 0.43 | 0 | 0.48 | 0.48 | 0 | 0.61 | 0.61 |
| **HSERTA** | 0 | 0.37 | 0.37 | 0 | 0.4 | 0.4 | 0 | 0.53 | 0.53 |
| **HSK** | 0.02 | 0.59 | 0.57 | 0.03 | 0.64 | 0.61 | 0.03 | 0.85 | 0.82 |
| **HSTPT** | 0.01 | 0.1 | 0.09 | 0.01 | 0.12 | 0.11 | 0.01 | 0.14 | 0.13 |
| **HXANt2r** | -0.09 | 0 | 0.09 | -0.11 | 0 | 0.11 | -0.12 | 0 | 0.12 |
| **HXPRT** | 0 | 0.61 | 0.61 | 0 | 0.68 | 0.68 | 0 | 0.86 | 0.86 |
| **HYPOE** | 0 | 1.23 | 1.23 | 0 | 1.35 | 1.35 | 0 | 1.73 | 1.73 |
| **IAMACt** | 0 | 0.17 | 0.17 | 0 | 0.22 | 0.22 | 0 | 0.24 | 0.24 |
| **IAMOHt** | 0 | 0.3 | 0.3 | 0 | 0.39 | 0.39 | 0 | 0.41 | 0.41 |
| **IAMOHtm** | -0.3 | 0 | 0.3 | -0.39 | 0 | 0.39 | -0.41 | 0 | 0.41 |
| **IBUTACt** | 0 | 0.32 | 0.32 | 0 | 0.38 | 0.38 | 0 | 0.43 | 0.43 |
| **IBUTOHt** | 0 | 1.23 | 1.23 | 0 | 1.35 | 1.35 | 0 | 1.73 | 1.73 |
| **IBUTOHtm** | -1.23 | 0 | 1.23 | -1.35 | 0 | 1.35 | -1.73 | 0 | 1.73 |
| **ICDHxm** | 0 | 0.3 | 0.3 | 0 | 0.4 | 0.4 | 0 | 0.41 | 0.41 |
| **ICDHy** | 0.05 | 0.42 | 0.37 | 0.06 | 0.55 | 0.49 | 0.07 | 0.58 | 0.51 |
| **ICDHym** | 0 | 0.33 | 0.33 | 0 | 0.41 | 0.41 | 0 | 0.45 | 0.45 |
| **ICL** | 0 | 0.33 | 0.33 | 0 | 0.44 | 0.44 | 0 | 0.46 | 0.46 |
| **ID3ACALDt** | 0 | 0.1 | 0.1 | 0 | 0.11 | 0.11 | 0 | 0.13 | 0.13 |
| **ID3ACALDtm** | 0 | 0.1 | 0.1 | 0 | 0.12 | 0.12 | 0 | 0.14 | 0.14 |
| **IG3PS** | 0.01 | 0.1 | 0.09 | 0.01 | 0.12 | 0.11 | 0.01 | 0.14 | 0.13 |
| **IGPDH** | 0.01 | 0.1 | 0.09 | 0.01 | 0.12 | 0.11 | 0.01 | 0.14 | 0.13 |
| **IGPS** | 0 | 0.14 | 0.14 | 0 | 0.17 | 0.17 | 0 | 0.2 | 0.2 |
| **ILETA** | -0.31 | 499.98 | 500.29 | -0.34 | 499.97 | 500.31 | -0.44 | 499.97 | 500.41 |
| **ILEt2r** | -0.29 | 0 | 0.29 | -0.32 | 0 | 0.32 | -0.41 | 0 | 0.41 |
| **ILEt6** | 0 | 0.2 | 0.2 | 0 | 0.23 | 0.23 | 0 | 0.29 | 0.29 |
| **ILEt7** | 0 | 0.2 | 0.2 | 0 | 0.23 | 0.23 | 0 | 0.29 | 0.29 |
| **IMPC** | -0.11 | -0.02 | 0.09 | -0.13 | -0.02 | 0.11 | -0.16 | -0.03 | 0.13 |
| **IMPD** | 0.01 | 0.09 | 0.08 | 0.01 | 0.11 | 0.1 | 0.01 | 0.12 | 0.11 |
| **IND3ETHt** | 0 | 0.1 | 0.1 | 0 | 0.12 | 0.12 | 0 | 0.14 | 0.14 |
| **IND3ETHtm** | -0.1 | 0 | 0.1 | -0.12 | 0 | 0.12 | -0.14 | 0 | 0.14 |
| **INDPYRD** | 0 | 0.1 | 0.1 | 0 | 0.12 | 0.12 | 0 | 0.14 | 0.14 |
| **INSK** | 0 | 1.23 | 1.23 | 0 | 1.35 | 1.35 | 0 | 1.73 | 1.73 |
| **IPC124PLC_SC** | 0 | 0.25 | 0.25 | 0 | 0.27 | 0.27 | 0 | 0.35 | 0.35 |
| **IPC126PLC_SC** | 0 | 0.25 | 0.25 | 0 | 0.27 | 0.27 | 0 | 0.35 | 0.35 |
| **IPC224PLC_SC** | 0 | 0.25 | 0.25 | 0 | 0.27 | 0.27 | 0 | 0.35 | 0.35 |
| **IPC226PLC_SC** | 0 | 0.25 | 0.25 | 0 | 0.27 | 0.27 | 0 | 0.35 | 0.35 |
| **IPC324PLC_SC** | 0 | 0.25 | 0.25 | 0 | 0.27 | 0.27 | 0 | 0.35 | 0.35 |
| **IPC326PLC_SC** | 0 | 0.25 | 0.25 | 0 | 0.27 | 0.27 | 0 | 0.35 | 0.35 |
| **IPCS124_SC** | 0 | 0.25 | 0.25 | 0 | 0.27 | 0.27 | 0 | 0.35 | 0.35 |
| **IPCS126_SC** | 0 | 0.25 | 0.25 | 0 | 0.27 | 0.27 | 0 | 0.35 | 0.35 |
| **IPCS224_SC** | 0 | 0.25 | 0.25 | 0 | 0.27 | 0.27 | 0 | 0.35 | 0.35 |
| **IPCS226_SC** | 0 | 0.25 | 0.25 | 0 | 0.27 | 0.27 | 0 | 0.35 | 0.35 |
| **IPCS324_SC** | 0 | 0.25 | 0.25 | 0 | 0.27 | 0.27 | 0 | 0.35 | 0.35 |
| **IPCS326_SC** | 0 | 0.25 | 0.25 | 0 | 0.27 | 0.27 | 0 | 0.35 | 0.35 |
| **IPMD** | 0.04 | 0.33 | 0.29 | 0.04 | 0.43 | 0.39 | 0.05 | 0.46 | 0.41 |
| **IPPMIa** | -0.33 | -0.04 | 0.29 | -0.43 | -0.04 | 0.39 | -0.46 | -0.05 | 0.41 |
| **IPPMIb** | -0.33 | -0.04 | 0.29 | -0.43 | -0.04 | 0.39 | -0.46 | -0.05 | 0.41 |
| **IPPS** | 0 | 0.28 | 0.28 | 0 | 0.35 | 0.35 | 0 | 0.39 | 0.39 |
| **IPPSm** | 0 | 0.33 | 0.33 | 0 | 0.43 | 0.43 | 0 | 0.46 | 0.46 |
| **KARA1im** | 0.07 | 1.3 | 1.23 | 0.08 | 1.43 | 1.35 | 0.1 | 1.82 | 1.72 |
| **KARA2im** | 0.02 | 0.4 | 0.38 | 0.03 | 0.43 | 0.4 | 0.03 | 0.56 | 0.53 |
| **KYN** | 0 | 0.14 | 0.14 | 0 | 0.17 | 0.17 | 0 | 0.19 | 0.19 |
| **L-LACD2cm** | 0 | 0.36 | 0.36 | 0 | 0.41 | 0.41 | 0 | 0.51 | 0.51 |
| **L-LACt2r** | -0.41 | 0 | 0.41 | -0.46 | 0 | 0.46 | -0.58 | 0 | 0.58 |
| **LALDO3** | 0 | 0.41 | 0.41 | 0 | 0.46 | 0.46 | 0 | 0.58 | 0.58 |
| **LCADi** | 0 | 0.41 | 0.41 | 0 | 0.46 | 0.46 | 0 | 0.58 | 0.58 |
| **LEUTA** | -0.28 | -0.04 | 0.24 | -0.35 | -0.04 | 0.31 | -0.38 | -0.05 | 0.33 |
| **LEUt2r** | -0.24 | 0 | 0.24 | -0.3 | 0 | 0.3 | -0.33 | 0 | 0.33 |
| **LEUt6** | 0 | 0.2 | 0.2 | 0 | 0.23 | 0.23 | 0 | 0.29 | 0.29 |
| **LEUt7** | 0 | 0.2 | 0.2 | 0 | 0.23 | 0.23 | 0 | 0.29 | 0.29 |
| **LGTHL** | 0 | 0.61 | 0.61 | 0 | 0.68 | 0.68 | 0 | 0.86 | 0.86 |
| **LPCAT_SC** | 0 | 0.35 | 0.35 | 0 | 0.39 | 0.39 | 0 | 0.49 | 0.49 |
| **LPP_SC** | 0 | 1.23 | 1.23 | 0 | 1.35 | 1.35 | 0 | 1.73 | 1.73 |
| **LYSt2r** | -0.14 | 0 | 0.14 | -0.17 | 0 | 0.17 | -0.19 | 0 | 0.19 |
| **MCITDm** | 0.04 | 0.17 | 0.13 | 0.04 | 0.21 | 0.17 | 0.05 | 0.24 | 0.19 |
| **MCITL2m** | 0 | 0.26 | 0.26 | 0 | 0.3 | 0.3 | 0 | 0.36 | 0.36 |
| **MCITSm** | 0 | 0.26 | 0.26 | 0 | 0.3 | 0.3 | 0 | 0.36 | 0.36 |
| **MDRPD** | 0 | 0.12 | 0.12 | 0 | 0.13 | 0.13 | 0 | 0.17 | 0.17 |
| **ME1m** | 0 | 1.64 | 1.64 | 0 | 1.81 | 1.81 | 0 | 2.3 | 2.3 |
| **ME2m** | 0 | 1.35 | 1.35 | 0 | 1.49 | 1.49 | 0 | 1.9 | 1.9 |
| **METAT** | 0 | 0.41 | 0.41 | 0 | 0.45 | 0.45 | 0 | 0.58 | 0.58 |
| **METB1** | 0 | 0.37 | 0.37 | 0 | 0.4 | 0.4 | 0 | 0.52 | 0.52 |
| **METS** | 0.01 | 0.16 | 0.15 | 0.01 | 0.17 | 0.16 | 0.01 | 0.22 | 0.21 |
| **METt2r** | -0.14 | 0 | 0.14 | -0.15 | 0 | 0.15 | -0.19 | 0 | 0.19 |
| **MEVK1** | 0 | 0.13 | 0.13 | 0 | 0.16 | 0.16 | 0 | 0.17 | 0.17 |
| **MEVK2** | 0 | 0.13 | 0.13 | 0 | 0.16 | 0.16 | 0 | 0.17 | 0.17 |
| **MEVK3** | 0 | 0.13 | 0.13 | 0 | 0.16 | 0.16 | 0 | 0.17 | 0.17 |
| **MEVK4** | 0 | 0.13 | 0.13 | 0 | 0.16 | 0.16 | 0 | 0.17 | 0.17 |
| **MFAPS_SC** | 0 | 0.05 | 0.05 | 0 | 0.06 | 0.06 | 0 | 0.07 | 0.07 |
| **MGSA** | 0 | 0.61 | 0.61 | 0 | 0.68 | 0.68 | 0 | 0.86 | 0.86 |
| **MI1PP** | 0 | 0.25 | 0.25 | 0 | 0.27 | 0.27 | 0 | 0.35 | 0.35 |
| **MOHMT** | 0 | 0.09 | 0.09 | 0 | 0.1 | 0.1 | 0 | 0.13 | 0.13 |
| **MTAP** | 0 | 0.12 | 0.12 | 0 | 0.13 | 0.13 | 0 | 0.17 | 0.17 |
| **MTHFC** | -0.1 | 0.21 | 0.31 | -0.11 | 0.25 | 0.36 | -0.14 | 0.29 | 0.43 |
| **MTHFD** | -1.2 | 0.21 | 1.41 | -1.41 | 0.25 | 1.66 | -1.68 | 0.29 | 1.97 |
| **MTHFD2i** | 0 | 1.23 | 1.23 | 0 | 1.45 | 1.45 | 0 | 1.73 | 1.73 |
| **MTHFR3** | 0.01 | 0.16 | 0.15 | 0.01 | 0.17 | 0.16 | 0.01 | 0.22 | 0.21 |
| **MTRI** | 0 | 0.12 | 0.12 | 0 | 0.13 | 0.13 | 0 | 0.17 | 0.17 |
| **NABTNO** | 0 | 0.05 | 0.05 | 0 | 0.06 | 0.06 | 0 | 0.07 | 0.07 |
| **NADK** | 0 | 1.23 | 1.23 | 0 | 1.35 | 1.35 | 0 | 1.73 | 1.73 |
| **NADKm** | 0 | 1.23 | 1.23 | 0 | 1.31 | 1.31 | 0 | 1.54 | 1.54 |
| **NADPPPS** | 0 | 1.23 | 1.23 | 0 | 1.35 | 1.35 | 0 | 1.73 | 1.73 |
| **NADPPPSm** | 0 | 1.23 | 1.23 | 0 | 1.31 | 1.31 | 0 | 1.54 | 1.54 |
| **NDP1** | 0 | 1.23 | 1.23 | 0 | 1.35 | 1.35 | 0 | 1.73 | 1.73 |
| **NDP3** | 0 | 1.23 | 1.23 | 0 | 1.35 | 1.35 | 0 | 1.73 | 1.73 |
| **NDP4** | 0 | 1.23 | 1.23 | 0 | 1.35 | 1.35 | 0 | 1.73 | 1.73 |
| **NDP7** | 0 | 1.23 | 1.23 | 0 | 1.35 | 1.35 | 0 | 1.73 | 1.73 |
| **NDPK2** | 0.21 | 1.44 | 1.23 | 0.23 | 1.59 | 1.36 | 0.29 | 2.02 | 1.73 |
| **NDPK3** | -0.62 | 0.49 | 1.11 | -0.68 | 0.54 | 1.22 | -0.87 | 0.69 | 1.56 |
| **NDPK4** | 0 | 0.08 | 0.08 | 0 | 0.1 | 0.1 | 0 | 0.12 | 0.12 |
| **NDPK5** | -0.08 | 1.23 | 1.31 | -0.08 | 1.35 | 1.43 | -0.11 | 1.73 | 1.84 |
| **NDPK6** | -500 | 0.61 | 500.61 | -500 | 0.68 | 500.68 | -500 | 0.86 | 500.86 |
| **NDPK7** | -0.08 | 500 | 500.08 | -0.09 | 500 | 500.09 | -0.11 | 500 | 500.11 |
| **NFORTYRt** | 0 | 0.07 | 0.07 | 0 | 0.08 | 0.08 | 0 | 0.09 | 0.09 |
| **NFTYROX** | 0 | 0.07 | 0.07 | 0 | 0.08 | 0.08 | 0 | 0.09 | 0.09 |
| **NH4tm** | -0.47 | 0 | 0.47 | -0.5 | 0 | 0.5 | -0.66 | 0 | 0.66 |
| **NTD1** | 0 | 1.23 | 1.23 | 0 | 1.35 | 1.35 | 0 | 1.73 | 1.73 |
| **NTD10** | 0 | 0.61 | 0.61 | 0 | 0.68 | 0.68 | 0 | 0.86 | 0.86 |
| **NTD11** | 0 | 1.23 | 1.23 | 0 | 1.35 | 1.35 | 0 | 1.73 | 1.73 |
| **NTD2** | 0 | 1.23 | 1.23 | 0 | 1.35 | 1.35 | 0 | 1.73 | 1.73 |
| **NTD3** | 0 | 1.23 | 1.23 | 0 | 1.35 | 1.35 | 0 | 1.73 | 1.73 |
| **NTD4** | 0 | 1.23 | 1.23 | 0 | 1.35 | 1.35 | 0 | 1.73 | 1.73 |
| **NTD5** | 0 | 1.23 | 1.23 | 0 | 1.35 | 1.35 | 0 | 1.73 | 1.73 |
| **NTD6** | 0 | 0.08 | 0.08 | 0 | 0.08 | 0.08 | 0 | 0.11 | 0.11 |
| **NTD7** | 0 | 1.23 | 1.23 | 0 | 1.35 | 1.35 | 0 | 1.73 | 1.73 |
| **NTD8** | 0 | 0.08 | 0.08 | 0 | 0.08 | 0.08 | 0 | 0.11 | 0.11 |
| **NTD9** | 0 | 1.23 | 1.23 | 0 | 1.35 | 1.35 | 0 | 1.73 | 1.73 |
| **NTP3** | 0 | 1.23 | 1.23 | 0 | 1.35 | 1.35 | 0 | 1.73 | 1.73 |
| **NTP4** | 0 | 1.23 | 1.23 | 0 | 1.35 | 1.35 | 0 | 1.73 | 1.73 |
| **OAAt** | 0 | 0.63 | 0.63 | 0 | 0.77 | 0.77 | 0 | 0.86 | 0.86 |
| **OBDHm** | 0 | 0.26 | 0.26 | 0 | 0.3 | 0.3 | 0 | 0.36 | 0.36 |
| **OCBTi** | 0.02 | 0.22 | 0.2 | 0.02 | 0.25 | 0.23 | 0.03 | 0.32 | 0.29 |
| **OHACT1** | 0 | 0.61 | 0.61 | 0 | 0.68 | 0.68 | 0 | 0.86 | 0.86 |
| **OHACT2** | 0 | 0.61 | 0.61 | 0 | 0.68 | 0.68 | 0 | 0.86 | 0.86 |
| **OHACT3** | 0 | 0.21 | 0.21 | 0 | 0.23 | 0.23 | 0 | 0.29 | 0.29 |
| **OHACT4** | 0 | 0.61 | 0.61 | 0 | 0.68 | 0.68 | 0 | 0.86 | 0.86 |
| **OHACT5** | 0 | 0.14 | 0.14 | 0 | 0.15 | 0.15 | 0 | 0.19 | 0.19 |
| **OMCDC** | 0.04 | 0.33 | 0.29 | 0.04 | 0.43 | 0.39 | 0.05 | 0.46 | 0.41 |
| **OMPDC** | 0.01 | 0.13 | 0.12 | 0.02 | 0.14 | 0.12 | 0.02 | 0.18 | 0.16 |
| **ORNDC** | 0 | 0.27 | 0.27 | 0 | 0.31 | 0.31 | 0 | 0.36 | 0.36 |
| **ORNTA** | 0 | 0.63 | 0.63 | 0 | 0.69 | 0.69 | 0 | 0.79 | 0.79 |
| **ORNTACim** | 0.02 | 0.65 | 0.63 | 0.02 | 0.71 | 0.69 | 0.03 | 0.82 | 0.79 |
| **ORNt2r** | -0.27 | 0 | 0.27 | -0.31 | 0 | 0.31 | -0.36 | 0 | 0.36 |
| **ORNt3m** | 0.02 | 0.65 | 0.63 | 0.02 | 0.71 | 0.69 | 0.03 | 0.82 | 0.79 |
| **ORPT** | -0.13 | -0.01 | 0.12 | -0.14 | -0.02 | 0.12 | -0.18 | -0.02 | 0.16 |
| **OXAGm** | 0.04 | 0.17 | 0.13 | 0.04 | 0.21 | 0.17 | 0.05 | 0.24 | 0.19 |
| **P5CDm** | 0 | 0.46 | 0.46 | 0 | 0.52 | 0.52 | 0 | 0.64 | 0.64 |
| **P5CR** | 0.02 | 0.65 | 0.63 | 0.02 | 0.72 | 0.7 | 0.03 | 0.83 | 0.8 |
| **PACALDt** | 0 | 0.17 | 0.17 | 0 | 0.19 | 0.19 | 0 | 0.24 | 0.24 |
| **PACALDtm** | 0 | 0.19 | 0.19 | 0 | 0.22 | 0.22 | 0 | 0.27 | 0.27 |
| **PAK_SC** | 0 | 1.23 | 1.23 | 0 | 1.35 | 1.35 | 0 | 1.73 | 1.73 |
| **PAN4Ptm** | -0.35 | 0 | 0.35 | -0.39 | 0 | 0.39 | -0.49 | 0 | 0.49 |
| **PANTS** | 0 | 0.09 | 0.09 | 0 | 0.1 | 0.1 | 0 | 0.13 | 0.13 |
| **PANTtm** | -0.09 | 0 | 0.09 | -0.1 | 0 | 0.1 | -0.13 | 0 | 0.13 |
| **PAPSR** | 0.01 | 0.57 | 0.56 | 0.01 | 0.63 | 0.62 | 0.01 | 0.81 | 0.8 |
| **PAPt** | -0.06 | 0 | 0.06 | -0.07 | 0 | 0.07 | -0.09 | 0 | 0.09 |
| **PAPtm** | -0.35 | 0 | 0.35 | -0.39 | 0 | 0.39 | -0.49 | 0 | 0.49 |
| **PDE1** | 0 | 0.61 | 0.61 | 0 | 0.68 | 0.68 | 0 | 0.86 | 0.86 |
| **PDHm** | 0 | 0.79 | 0.79 | 0 | 1 | 1 | 0 | 1.08 | 1.08 |
| **PETOHM_SC** | 0 | 0.05 | 0.05 | 0 | 0.06 | 0.06 | 0 | 0.07 | 0.07 |
| **PFK26** | 0 | 1.23 | 1.23 | 0 | 1.35 | 1.35 | 0 | 1.73 | 1.73 |
| **PGCD** | 0 | 0.68 | 0.68 | 0 | 0.78 | 0.78 | 0 | 0.96 | 0.96 |
| **PGMT** | -0.82 | -0.21 | 0.61 | -0.91 | -0.23 | 0.68 | -1.15 | -0.29 | 0.86 |
| **PHEACt** | 0 | 0.14 | 0.14 | 0 | 0.15 | 0.15 | 0 | 0.19 | 0.19 |
| **PHETA1** | -0.18 | -0.02 | 0.16 | -0.21 | -0.02 | 0.19 | -0.26 | -0.02 | 0.24 |
| **PHEt2r** | -0.17 | 0 | 0.17 | -0.19 | 0 | 0.19 | -0.24 | 0 | 0.24 |
| **PI35BP5P_SC** | 0 | 1.23 | 1.23 | 0 | 1.35 | 1.35 | 0 | 1.73 | 1.73 |
| **PI3P5K_SC** | 0 | 1.23 | 1.23 | 0 | 1.35 | 1.35 | 0 | 1.73 | 1.73 |
| **PI45BP5P_SC** | 0 | 1.23 | 1.23 | 0 | 1.35 | 1.35 | 0 | 1.73 | 1.73 |
| **PI4P5K_SC** | 0 | 1.23 | 1.23 | 0 | 1.35 | 1.35 | 0 | 1.73 | 1.73 |
| **PINOS_SC** | 0 | 0.25 | 0.25 | 0 | 0.27 | 0.27 | 0 | 0.35 | 0.35 |
| **PIt2m** | -2.55 | 500 | 502.55 | -2.85 | 500 | 502.85 | -3.29 | 500 | 503.29 |
| **PIt2r** | 0.02 | 0.33 | 0.31 | 0.03 | 0.37 | 0.34 | 0.03 | 0.47 | 0.44 |
| **PIt5m** | -0.35 | 0 | 0.35 | -0.47 | 0 | 0.47 | -0.48 | 0 | 0.48 |
| **PLBPC_SC** | 0 | 0.25 | 0.25 | 0 | 0.28 | 0.28 | 0 | 0.35 | 0.35 |
| **PLD_SC** | 0 | 0.41 | 0.41 | 0 | 0.45 | 0.45 | 0 | 0.58 | 0.58 |
| **PMETM_SC** | 0 | 0.05 | 0.05 | 0 | 0.06 | 0.06 | 0 | 0.07 | 0.07 |
| **PMEVK** | 0 | 0.13 | 0.13 | 0 | 0.16 | 0.16 | 0 | 0.17 | 0.17 |
| **PMI12346PH** | 0 | 1.23 | 1.23 | 0 | 1.35 | 1.35 | 0 | 1.73 | 1.73 |
| **PMI12346PS** | 0 | 1.23 | 1.23 | 0 | 1.35 | 1.35 | 0 | 1.73 | 1.73 |
| **PMI1346PH** | 0 | 1.23 | 1.23 | 0 | 1.35 | 1.35 | 0 | 1.73 | 1.73 |
| **PMI1346PS** | 0 | 1.23 | 1.23 | 0 | 1.35 | 1.35 | 0 | 1.73 | 1.73 |
| **PMPK** | 0 | 0.41 | 0.41 | 0 | 0.45 | 0.45 | 0 | 0.58 | 0.58 |
| **PNTOt2** | -0.09 | 0 | 0.09 | -0.1 | 0 | 0.1 | -0.13 | 0 | 0.13 |
| **POLYAO** | 0 | 0.05 | 0.05 | 0 | 0.06 | 0.06 | 0 | 0.07 | 0.07 |
| **POLYAO3** | 0 | 0.09 | 0.09 | 0 | 0.1 | 0.1 | 0 | 0.13 | 0.13 |
| **PPAm** | 0 | 1.22 | 1.22 | 0 | 1.3 | 1.3 | 0 | 1.5 | 1.5 |
| **PPCK** | 0 | 1.23 | 1.23 | 0 | 1.35 | 1.35 | 0 | 1.73 | 1.73 |
| **PPND** | 0 | 0.16 | 0.16 | 0 | 0.18 | 0.18 | 0 | 0.22 | 0.22 |
| **PPND2** | 0 | 0.18 | 0.18 | 0 | 0.21 | 0.21 | 0 | 0.25 | 0.25 |
| **PPNDH** | 0.02 | 0.21 | 0.19 | 0.02 | 0.23 | 0.21 | 0.02 | 0.3 | 0.28 |
| **PPYRDC** | 0 | 0.19 | 0.19 | 0 | 0.22 | 0.22 | 0 | 0.27 | 0.27 |
| **PRAGSr** | 0.01 | 0.1 | 0.09 | 0.01 | 0.12 | 0.11 | 0.02 | 0.14 | 0.12 |
| **PRAIS** | 0.01 | 0.1 | 0.09 | 0.01 | 0.12 | 0.11 | 0.02 | 0.14 | 0.12 |
| **PRAIi** | 0 | 0.14 | 0.14 | 0 | 0.17 | 0.17 | 0 | 0.2 | 0.2 |
| **PRAMPC** | 0.01 | 0.1 | 0.09 | 0.01 | 0.12 | 0.11 | 0.01 | 0.14 | 0.13 |
| **PRASCS** | 0.01 | 0.1 | 0.09 | 0.01 | 0.12 | 0.11 | 0.02 | 0.14 | 0.12 |
| **PRATPP** | 0.01 | 0.1 | 0.09 | 0.01 | 0.12 | 0.11 | 0.01 | 0.14 | 0.13 |
| **PRFGS** | 0.01 | 0.1 | 0.09 | 0.01 | 0.12 | 0.11 | 0.02 | 0.14 | 0.12 |
| **PRMICIi** | 0.01 | 0.1 | 0.09 | 0.01 | 0.12 | 0.11 | 0.01 | 0.14 | 0.13 |
| **PRO1xm** | 0 | 0.63 | 0.63 | 0 | 0.69 | 0.69 | 0 | 0.8 | 0.8 |
| **PROt2r** | -0.3 | 0 | 0.3 | -0.35 | 0 | 0.35 | -0.41 | 0 | 0.41 |
| **PROtm** | 0 | 0.63 | 0.63 | 0 | 0.69 | 0.69 | 0 | 0.8 | 0.8 |
| **PRPPS** | 0.04 | 0.65 | 0.61 | 0.04 | 0.72 | 0.68 | 0.05 | 0.92 | 0.87 |
| **PSCVTi** | 0.03 | 0.23 | 0.2 | 0.04 | 0.25 | 0.21 | 0.05 | 0.32 | 0.27 |
| **PSERDm_SC** | 0 | 0.05 | 0.05 | 0 | 0.06 | 0.06 | 0 | 0.07 | 0.07 |
| **PSERDv_SC** | 0 | 0.04 | 0.04 | 0 | 0.05 | 0.05 | 0 | 0.06 | 0.06 |
| **PSERS_SC** | -1.15 | 0.05 | 1.2 | -1.26 | 0.06 | 1.32 | -1.42 | 0.07 | 1.49 |
| **PSERSm_SC** | 0 | 1.15 | 1.15 | 0 | 1.26 | 1.26 | 0 | 1.42 | 1.42 |
| **PSERT** | 0 | 0.68 | 0.68 | 0 | 0.78 | 0.78 | 0 | 0.96 | 0.96 |
| **PSP_L** | 0 | 0.68 | 0.68 | 0 | 0.78 | 0.78 | 0 | 0.96 | 0.96 |
| **PTPATi** | 0 | 0.35 | 0.35 | 0 | 0.39 | 0.39 | 0 | 0.49 | 0.49 |
| **PUNP2** | -0.41 | 0.08 | 0.49 | -0.47 | 0.08 | 0.55 | -0.58 | 0.11 | 0.69 |
| **PUNP4** | 0 | 0.08 | 0.08 | 0 | 0.08 | 0.08 | 0 | 0.11 | 0.11 |
| **PUNP5** | -0.41 | 0.61 | 1.02 | -0.47 | 0.68 | 1.15 | -0.58 | 0.86 | 1.44 |
| **PUNP6** | 0 | 0.41 | 0.41 | 0 | 0.47 | 0.47 | 0 | 0.58 | 0.58 |
| **PUNP7** | 0 | 0.61 | 0.61 | 0 | 0.68 | 0.68 | 0 | 0.86 | 0.86 |
| **PYDAMK** | 0 | 1.23 | 1.23 | 0 | 1.35 | 1.35 | 0 | 1.73 | 1.73 |
| **PYNP2r** | -0.61 | 0.61 | 1.22 | -0.68 | 0.68 | 1.36 | -0.86 | 0.86 | 1.72 |
| **PYR5CDm** | 0 | 0.63 | 0.63 | 0 | 0.69 | 0.69 | 0 | 0.8 | 0.8 |
| **RBK** | 0 | 1.23 | 1.23 | 0 | 1.35 | 1.35 | 0 | 1.73 | 1.73 |
| **RNDR1** | 0 | 0.08 | 0.08 | 0 | 0.08 | 0.08 | 0 | 0.11 | 0.11 |
| **RNDR2** | 0 | 0.08 | 0.08 | 0 | 0.08 | 0.08 | 0 | 0.11 | 0.11 |
| **RNDR3** | 0 | 0.08 | 0.08 | 0 | 0.09 | 0.09 | 0 | 0.11 | 0.11 |
| **RNDR4** | 0 | 0.09 | 0.09 | 0 | 0.1 | 0.1 | 0 | 0.12 | 0.12 |
| **RNTR1** | 0 | 0.08 | 0.08 | 0 | 0.08 | 0.08 | 0 | 0.11 | 0.11 |
| **RNTR2** | 0 | 0.08 | 0.08 | 0 | 0.08 | 0.08 | 0 | 0.11 | 0.11 |
| **RNTR3** | 0 | 0.08 | 0.08 | 0 | 0.09 | 0.09 | 0 | 0.11 | 0.11 |
| **RNTR4** | 0 | 0.09 | 0.09 | 0 | 0.1 | 0.1 | 0 | 0.12 | 0.12 |
| **SACCD1** | 0.04 | 0.17 | 0.13 | 0.04 | 0.21 | 0.17 | 0.05 | 0.24 | 0.19 |
| **SACCD2** | 0.04 | 0.17 | 0.13 | 0.04 | 0.21 | 0.17 | 0.05 | 0.24 | 0.19 |
| **SADT** | 0 | 1.23 | 1.23 | 0 | 1.35 | 1.35 | 0 | 1.73 | 1.73 |
| **SBTD_D2** | 0 | 1.23 | 1.23 | 0 | 1.45 | 1.45 | 0 | 1.73 | 1.73 |
| **SBTR** | 0 | 1.23 | 1.23 | 0 | 1.45 | 1.45 | 0 | 1.73 | 1.73 |
| **SBT_Dt** | -0.57 | 0 | 0.57 | -0.63 | 0 | 0.63 | -0.8 | 0 | 0.8 |
| **SERATi** | 0 | 0.17 | 0.17 | 0 | 0.19 | 0.19 | 0 | 0.24 | 0.24 |
| **SERD_L** | 0 | 0.61 | 0.61 | 0 | 0.71 | 0.71 | 0 | 0.86 | 0.86 |
| **SERt2m** | -0.27 | 1.15 | 1.42 | -0.29 | 1.26 | 1.55 | -0.38 | 1.42 | 1.8 |
| **SERt2r** | -0.42 | 0 | 0.42 | -0.51 | 0 | 0.51 | -0.58 | 0 | 0.58 |
| **SHK3D** | 0.03 | 0.23 | 0.2 | 0.04 | 0.25 | 0.21 | 0.05 | 0.32 | 0.27 |
| **SHKK** | 0.03 | 0.23 | 0.2 | 0.04 | 0.25 | 0.21 | 0.05 | 0.32 | 0.27 |
| **SO3ti** | 0 | 0.57 | 0.57 | 0 | 0.63 | 0.63 | 0 | 0.8 | 0.8 |
| **SO4ti** | 0.01 | 0.58 | 0.57 | 0.01 | 0.64 | 0.63 | 0.01 | 0.81 | 0.8 |
| **SPMDAT1** | 0 | 0.05 | 0.05 | 0 | 0.06 | 0.06 | 0 | 0.07 | 0.07 |
| **SPMS** | 0 | 0.12 | 0.12 | 0 | 0.13 | 0.13 | 0 | 0.17 | 0.17 |
| **SPRMS** | 0 | 0.09 | 0.09 | 0 | 0.1 | 0.1 | 0 | 0.13 | 0.13 |
| **SULR** | -0.17 | -0.01 | 0.16 | -0.2 | -0.01 | 0.19 | -0.24 | -0.01 | 0.23 |
| **TAGL_SC** | 0 | 0.35 | 0.35 | 0 | 0.39 | 0.39 | 0 | 0.49 | 0.49 |
| **THFATm** | 0 | 1.23 | 1.23 | 0 | 1.31 | 1.31 | 0 | 1.54 | 1.54 |
| **THIORDXi** | 0 | 0.1 | 0.1 | 0 | 0.12 | 0.12 | 0 | 0.14 | 0.14 |
| **THMP** | 0 | 1.23 | 1.23 | 0 | 1.35 | 1.35 | 0 | 1.73 | 1.73 |
| **THRAi** | 0 | 0.54 | 0.54 | 0 | 0.59 | 0.59 | 0 | 0.78 | 0.78 |
| **THRD_L** | 0 | 0.51 | 0.51 | 0 | 0.55 | 0.55 | 0 | 0.72 | 0.72 |
| **THRD_Lm** | 0 | 0.47 | 0.47 | 0 | 0.5 | 0.5 | 0 | 0.66 | 0.66 |
| **THRS** | 0.02 | 0.59 | 0.57 | 0.03 | 0.64 | 0.61 | 0.03 | 0.85 | 0.82 |
| **THRt2m** | 0 | 0.47 | 0.47 | 0 | 0.5 | 0.5 | 0 | 0.66 | 0.66 |
| **THRt2r** | -0.37 | 0 | 0.37 | -0.41 | 0 | 0.41 | -0.52 | 0 | 0.52 |
| **THYMt3r** | 0 | 0.11 | 0.11 | 0 | 0.13 | 0.13 | 0 | 0.16 | 0.16 |
| **TMDK1** | 0 | 1.23 | 1.23 | 0 | 1.35 | 1.35 | 0 | 1.73 | 1.73 |
| **TMDPK** | 0 | 1.23 | 1.23 | 0 | 1.35 | 1.35 | 0 | 1.73 | 1.73 |
| **TMDPP** | 0 | 0.11 | 0.11 | 0 | 0.13 | 0.13 | 0 | 0.16 | 0.16 |
| **TMDS** | 0 | 0.11 | 0.11 | 0 | 0.13 | 0.13 | 0 | 0.16 | 0.16 |
| **TMN** | 0 | 0.41 | 0.41 | 0 | 0.45 | 0.45 | 0 | 0.58 | 0.58 |
| **TMPKr** | -1.23 | 0 | 1.23 | -1.35 | 0 | 1.35 | -1.73 | 0 | 1.73 |
| **TMPPP** | 0 | 0.41 | 0.41 | 0 | 0.45 | 0.45 | 0 | 0.58 | 0.58 |
| **TRDR** | 0.01 | 0.58 | 0.57 | 0.01 | 0.63 | 0.62 | 0.01 | 0.81 | 0.8 |
| **TRE6PP** | 0 | 0.41 | 0.41 | 0 | 0.45 | 0.45 | 0 | 0.58 | 0.58 |
| **TRE6PS** | 0 | 0.41 | 0.41 | 0 | 0.45 | 0.45 | 0 | 0.58 | 0.58 |
| **TREH** | 0 | 0.41 | 0.41 | 0 | 0.45 | 0.45 | 0 | 0.58 | 0.58 |
| **TREHv** | 0 | 0.41 | 0.41 | 0 | 0.45 | 0.45 | 0 | 0.58 | 0.58 |
| **TREt2v** | 0 | 0.41 | 0.41 | 0 | 0.45 | 0.45 | 0 | 0.58 | 0.58 |
| **TRIGS_SC** | 0 | 0.35 | 0.35 | 0 | 0.39 | 0.39 | 0 | 0.49 | 0.49 |
| **TRPO2** | 0 | 0.14 | 0.14 | 0 | 0.17 | 0.17 | 0 | 0.19 | 0.19 |
| **TRPS1** | 0 | 0.14 | 0.14 | 0 | 0.17 | 0.17 | 0 | 0.2 | 0.2 |
| **TRPTA** | 0 | 0.1 | 0.1 | 0 | 0.12 | 0.12 | 0 | 0.14 | 0.14 |
| **TRPt2r** | -0.1 | 0 | 0.1 | -0.11 | 0 | 0.11 | -0.13 | 0 | 0.13 |
| **TTDCAtr** | -0.05 | 0 | 0.05 | -0.06 | 0 | 0.06 | -0.07 | 0 | 0.07 |
| **TYRNFT** | 0 | 0.14 | 0.14 | 0 | 0.16 | 0.16 | 0 | 0.19 | 0.19 |
| **TYRTAi** | 0 | 0.18 | 0.18 | 0 | 0.21 | 0.21 | 0 | 0.25 | 0.25 |
| **TYRTAim** | 0 | 0.18 | 0.18 | 0 | 0.21 | 0.21 | 0 | 0.25 | 0.25 |
| **TYRt2m** | -0.18 | 0 | 0.18 | -0.21 | 0 | 0.21 | -0.25 | 0 | 0.25 |
| **TYRt2r** | -0.17 | 0 | 0.17 | -0.19 | 0 | 0.19 | -0.24 | 0 | 0.24 |
| **TYRt6** | 0 | 0.2 | 0.2 | 0 | 0.23 | 0.23 | 0 | 0.29 | 0.29 |
| **TYRt7** | 0 | 0.2 | 0.2 | 0 | 0.23 | 0.23 | 0 | 0.29 | 0.29 |
| **UMPK** | 0.01 | 1.24 | 1.23 | 0.01 | 1.36 | 1.35 | 0.01 | 1.73 | 1.72 |
| **UNK3** | 0 | 0.12 | 0.12 | 0 | 0.13 | 0.13 | 0 | 0.17 | 0.17 |
| **UPPRT** | 0 | 0.61 | 0.61 | 0 | 0.68 | 0.68 | 0 | 0.86 | 0.86 |
| **UREA2t2** | -0.2 | 0 | 0.2 | -0.23 | 0 | 0.23 | -0.29 | 0 | 0.29 |
| **UREASE** | 0 | 0.18 | 0.18 | 0 | 0.2 | 0.2 | 0 | 0.25 | 0.25 |
| **URIDK2r** | -500 | 0.61 | 500.61 | -500 | 0.68 | 500.68 | -500 | 0.86 | 500.86 |
| **URIK1** | 0 | 1.23 | 1.23 | 0 | 1.35 | 1.35 | 0 | 1.73 | 1.73 |
| **URIK2** | 0 | 1.23 | 1.23 | 0 | 1.35 | 1.35 | 0 | 1.73 | 1.73 |
| **UTPH1** | 0 | 0.61 | 0.61 | 0 | 0.68 | 0.68 | 0 | 0.86 | 0.86 |
| **VALTA** | -3.13 | -0.03 | 3.1 | -3.24 | -0.04 | 3.2 | -3.57 | -0.05 | 3.52 |
| **VALTAim** | 0 | 3.1 | 3.1 | 0 | 3.2 | 3.2 | 0 | 3.53 | 3.53 |
| **VALt2m** | 0 | 3.1 | 3.1 | 0 | 3.2 | 3.2 | 0 | 3.53 | 3.53 |
| **VALt2r** | -0.61 | 0 | 0.61 | -0.71 | 0 | 0.71 | -0.86 | 0 | 0.86 |
| **XANt** | -0.08 | 0 | 0.08 | -0.1 | 0 | 0.1 | -0.12 | 0 | 0.12 |
| **XPPT** | 0 | 0.61 | 0.61 | 0 | 0.68 | 0.68 | 0 | 0.86 | 0.86 |
| **EX_ac(e)** | 0 | 1.04 | 1.04 | 0 | 1.76 | 1.76 | 0 | 1.43 | 1.43 |
| **EX_arab-L(e)** | 0 | 0 | 0 | -1.2 | 0 | 1.2 | -1.2 | -0.16 | 1.04 |
| **ACONT** | -499.88 | 500 | 999.88 | -499.86 | 500 | 999.86 | -499.83 | 500 | 999.83 |
| **ACONTm** | -499.88 | 500 | 999.88 | -499.86 | 500 | 999.86 | -499.83 | 500 | 999.83 |
| **ADK3** | -500 | 499.89 | 999.89 | -500 | 499.87 | 999.87 | -500 | 499.84 | 999.84 |
| **ALDD2y** | 0 | 1.15 | 1.15 | 0 | 1.99 | 1.99 | 0 | 1.6 | 1.6 |
| **ARAB-Lt** | 0 | 0 | 0 | 0 | 1.2 | 1.2 | 0.16 | 1.2 | 1.04 |
| **ARABR** | 0 | 0 | 0 | 0 | 1.2 | 1.2 | 0.16 | 1.2 | 1.04 |
| **ASPt2n** | -500 | 499.79 | 999.79 | -500 | 499.91 | 999.91 | -500 | 499.68 | 999.68 |
| **ASPt5n** | -500 | 499.79 | 999.79 | -500 | 499.91 | 999.91 | -500 | 499.68 | 999.68 |
| **CITtcm** | -499.95 | 500 | 999.95 | -499.94 | 500 | 999.94 | -499.93 | 500 | 999.93 |
| **CO2tn** | -500 | 499.79 | 999.79 | -500 | 499.91 | 999.91 | -500 | 499.68 | 999.68 |
| **G6PI3** | -495.96 | 500 | 995.96 | -496.99 | 500 | 996.99 | -496.72 | 500 | 996.72 |
| **GALT** | -499.79 | 500 | 999.79 | -499.77 | 500 | 999.77 | -499.71 | 500 | 999.71 |
| **GALU** | -499.79 | 500 | 999.79 | -499.77 | 500 | 999.77 | -499.71 | 500 | 999.71 |
| **H2Otn** | -500 | 499.79 | 999.79 | -500 | 499.91 | 999.91 | -500 | 499.68 | 999.68 |
| **HCO3E** | -499.79 | 500 | 999.79 | -499.91 | 500 | 999.91 | -499.68 | 500 | 999.68 |
| **HCO3En** | -499.79 | 500 | 999.79 | -499.91 | 500 | 999.91 | -499.68 | 500 | 999.68 |
| **HCO3tn** | -500 | 499.79 | 999.79 | -500 | 499.91 | 999.91 | -500 | 499.68 | 999.68 |
| **MDH** | -500 | 1.11 | 501.11 | -500 | 1.62 | 501.62 | -500 | 1.48 | 501.48 |
| **NDPK1** | -499.89 | 500 | 999.89 | -499.87 | 500 | 999.87 | -499.84 | 500 | 999.84 |
| **NH4t** | 0.68 | 500 | 499.32 | 0.76 | 500 | 499.24 | 0.96 | 500 | 499.04 |
| **NH4ti** | 0 | 499.32 | 499.32 | 0 | 499.24 | 499.24 | 0 | 499.04 | 499.04 |
| **PGI** | -495.96 | 500 | 995.96 | -496.99 | 500 | 996.99 | -496.72 | 500 | 996.72 |
| **UGLT** | -500 | 499.79 | 999.79 | -500 | 499.77 | 999.77 | -500 | 499.71 | 999.71 |

**Table S1c.** The minimum and maximum allowable flux values for reaction whose difference of flux span between engineered cofactor imbalanced and engineered cofactor balanced models is given by, 0.0 <Span_ECI_ -Span_ECB_ < 0.01.

| **Reaction** | **Wild type**  **model** | | | **Engineered cofactor**  **imbalanced model** | | | **Engineered cofactor**  **balanced model** | | |
| --- | --- | --- | --- | --- | --- | --- | --- | --- | --- |
|  | **Min**  **Flux** | **Max**  **Flux** | **Span** | **Min**  **Flux** | **Max**  **Flux** | **Span** | **Min**  **Flux** | **Max**  **Flux** | **Min**  **Flux** |
| **biomass_SC5_notrace** | 0.12 | 0.14 | 0.02 | 0.14 | 0.15 | 0.01 | 0.17 | 0.19 | 0.02 |
| **EX_acald(e)** | 0 | 1.26 | 1.26 | 0 | 1.74 | 1.74 | 0 | 1.73 | 1.73 |
| **EX_akg(e)** | 0 | 0.3 | 0.3 | 0 | 0.4 | 0.4 | 0 | 0.41 | 0.41 |
| **EX_btd-RR(e)** | 0 | 1.26 | 1.26 | 0 | 1.74 | 1.74 | 0 | 1.73 | 1.73 |
| **EX_epist(e)** | 0 | 0.01 | 0.01 | 0 | 0.01 | 0.01 | 0 | 0.02 | 0.02 |
| **EX_ergst(e)** | 0 | 0.01 | 0.01 | 0 | 0.01 | 0.01 | 0 | 0.02 | 0.02 |
| **EX_fecost(e)** | 0 | 0.01 | 0.01 | 0 | 0.01 | 0.01 | 0 | 0.02 | 0.02 |
| **EX_gcald(e)** | 0 | 0 | 0 | 0 | 0 | 0 | 0 | 0 | 0 |
| **EX_glx(e)** | 0 | 0.3 | 0.3 | 0 | 0.4 | 0.4 | 0 | 0.41 | 0.41 |
| **EX_lanost(e)** | 0 | 0.02 | 0.02 | 0 | 0.03 | 0.03 | 0 | 0.03 | 0.03 |
| **EX_nadp(e)** | 0 | 0.04 | 0.04 | 0 | 0.04 | 0.04 | 0 | 0.05 | 0.05 |
| **EX_o2(e)** | -0.3 | -0.02 | 0.28 | -0.3 | -0.01 | 0.29 | -0.3 | -0.01 | 0.29 |
| **EX_pyr(e)** | 0 | 1.26 | 1.26 | 0 | 1.74 | 1.74 | 0 | 1.73 | 1.73 |
| **EX_zymst(e)** | 0 | 0.01 | 0.01 | 0 | 0.02 | 0.02 | 0 | 0.02 | 0.02 |
| **2HMHMBQMTm** | 0 | 0 | 0 | 0 | 0 | 0 | 0 | 0 | 0 |
| **2HP6MPMOm** | 0 | 0 | 0 | 0 | 0 | 0 | 0 | 0 | 0 |
| **2HPMBQMTm** | 0 | 0 | 0 | 0 | 0 | 0 | 0 | 0 | 0 |
| **2HPMMBQMOm** | 0 | 0 | 0 | 0 | 0 | 0 | 0 | 0 | 0 |
| **34HPLFM** | 0 | 0 | 0 | 0 | 0 | 0 | 0 | 0 | 0 |
| **3DH5HPBMTm** | 0 | 0 | 0 | 0 | 0 | 0 | 0 | 0 | 0 |
| **3DH5HPBtm** | 0 | 0 | 0 | 0 | 0 | 0 | 0 | 0 | 0 |
| **3HAO** | 0 | 0.02 | 0.02 | 0 | 0.03 | 0.03 | 0 | 0.03 | 0.03 |
| **3HPH5MBDCm** | 0 | 0 | 0 | 0 | 0 | 0 | 0 | 0 | 0 |
| **3OPHB5Hm** | 0 | 0 | 0 | 0 | 0 | 0 | 0 | 0 | 0 |
| **3OPHB_5tm** | 0 | 0 | 0 | 0 | 0 | 0 | 0 | 0 | 0 |
| **4HBZCOAFm** | 0 | 0 | 0 | 0 | 0 | 0 | 0 | 0 | 0 |
| **4HBZFm** | 0 | 0 | 0 | 0 | 0 | 0 | 0 | 0 | 0 |
| **4HBZtm** | 0 | 0 | 0 | 0 | 0 | 0 | 0 | 0 | 0 |
| **6PGLter** | -0.02 | 0 | 0.02 | -0.03 | 0 | 0.03 | -0.03 | 0 | 0.03 |
| **ABTA** | 0 | 0.3 | 0.3 | 0 | 0.41 | 0.41 | 0 | 0.41 | 0.41 |
| **ACACT4p** | 0 | 0 | 0 | 0 | 0 | 0 | 0 | 0 | 0 |
| **ACACT5p** | 0 | 0 | 0 | 0 | 0 | 0 | 0 | 0 | 0 |
| **ACACT6p** | 0 | 0 | 0 | 0 | 0 | 0 | 0 | 0 | 0 |
| **ACACT7p** | 0 | 0 | 0 | 0 | 0 | 0 | 0 | 0 | 0 |
| **ACACT8p** | 0 | 0 | 0 | 0 | 0 | 0 | 0 | 0 | 0 |
| **ACACT9p** | 0 | 0 | 0 | 0 | 0 | 0 | 0 | 0 | 0 |
| **ACALDCD** | 0 | 1.26 | 1.26 | 0 | 1.74 | 1.74 | 0 | 1.73 | 1.73 |
| **ACALDt** | -1.26 | 0 | 1.26 | -1.74 | 0 | 1.74 | -1.73 | 0 | 1.73 |
| **ACCOACrm** | 0 | 0 | 0 | 0 | 0 | 0 | 0 | 0 | 0 |
| **ACOAO4p** | 0 | 0 | 0 | 0 | 0 | 0 | 0 | 0 | 0 |
| **ACOAO5p** | 0 | 0 | 0 | 0 | 0 | 0 | 0 | 0 | 0 |
| **ACOAO6p** | 0 | 0 | 0 | 0 | 0 | 0 | 0 | 0 | 0 |
| **ACOAO7p** | 0 | 0 | 0 | 0 | 0 | 0 | 0 | 0 | 0 |
| **ACOAO8p** | 0 | 0 | 0 | 0 | 0 | 0 | 0 | 0 | 0 |
| **ACOAO9p** | 0 | 0 | 0 | 0 | 0 | 0 | 0 | 0 | 0 |
| **ACRNtp** | 0 | 0 | 0 | 0 | 0 | 0 | 0 | 0 | 0 |
| **ACSp** | 0 | 0 | 0 | 0 | 0 | 0 | 0 | 0 | 0 |
| **ACtp** | 0 | 0 | 0 | 0 | 0 | 0 | 0 | 0 | 0 |
| **AHCYStm** | 0 | 0 | 0 | 0 | 0 | 0 | 0 | 0 | 0 |
| **AHMMPS** | 0 | 0 | 0 | 0 | 0 | 0 | 0 | 0 | 0 |
| **AKGDam** | 0 | 0.3 | 0.3 | 0 | 0.41 | 0.41 | 0 | 0.41 | 0.41 |
| **AKGDbm** | 0 | 0.3 | 0.3 | 0 | 0.41 | 0.41 | 0 | 0.41 | 0.41 |
| **AKGtp** | 0 | 0 | 0 | 0 | 0 | 0 | 0 | 0 | 0 |
| **AKP1** | 0 | 0 | 0 | 0 | 0 | 0 | 0 | 0 | 0 |
| **ALCD19y** | 0 | 0 | 0 | 0 | 0 | 0 | 0 | 0 | 0 |
| **AMETtm** | 0 | 0 | 0 | 0 | 0 | 0 | 0 | 0 | 0 |
| **ASPGLUtp** | 0 | 0 | 0 | 0 | 0 | 0 | 0 | 0 | 0 |
| **ASPOcm** | 0 | 0.04 | 0.04 | 0 | 0.04 | 0.04 | 0 | 0.05 | 0.05 |
| **ASPTAp** | 0 | 0 | 0 | 0 | 0 | 0 | 0 | 0 | 0 |
| **ATPtp-H** | 0 | 0 | 0 | 0 | 0 | 0 | 0 | 0 | 0 |
| **BTDD-RR** | -1.26 | 0 | 1.26 | -1.74 | 0 | 1.74 | -1.73 | 0 | 1.73 |
| **BTDt-RR** | 0 | 1.26 | 1.26 | 0 | 1.74 | 1.74 | 0 | 1.73 | 1.73 |
| **C14STR** | 0 | 0.01 | 0.01 | 0 | 0.02 | 0.02 | 0 | 0.02 | 0.02 |
| **C22STDS** | 0 | 0.01 | 0.01 | 0 | 0.01 | 0.01 | 0 | 0.02 | 0.02 |
| **C22STDSx** | 0 | 0.01 | 0.01 | 0 | 0.01 | 0.01 | 0 | 0.02 | 0.02 |
| **C24STRer** | 0 | 0.01 | 0.01 | 0 | 0.01 | 0.01 | 0 | 0.02 | 0.02 |
| **C3STDH1** | 0 | 0.01 | 0.01 | 0 | 0.02 | 0.02 | 0 | 0.02 | 0.02 |
| **C3STDH2** | 0 | 0.01 | 0.01 | 0 | 0.02 | 0.02 | 0 | 0.02 | 0.02 |
| **C3STKR1** | 0 | 0.01 | 0.01 | 0 | 0.02 | 0.02 | 0 | 0.02 | 0.02 |
| **C3STKR2** | 0 | 0.01 | 0.01 | 0 | 0.02 | 0.02 | 0 | 0.02 | 0.02 |
| **C4STMO1** | 0 | 0.01 | 0.01 | 0 | 0.02 | 0.02 | 0 | 0.02 | 0.02 |
| **C4STMO2** | 0 | 0.01 | 0.01 | 0 | 0.02 | 0.02 | 0 | 0.02 | 0.02 |
| **C5STDS** | 0 | 0.01 | 0.01 | 0 | 0.01 | 0.01 | 0 | 0.02 | 0.02 |
| **C8STI** | 0 | 0.01 | 0.01 | 0 | 0.01 | 0.01 | 0 | 0.02 | 0.02 |
| **CATp** | 0 | 0 | 0 | 0 | 0 | 0 | 0 | 0 | 0 |
| **CER1_24ter** | 0 | 0 | 0 | 0 | 0 | 0 | 0 | 0 | 0 |
| **CER1_26ter** | 0 | 0 | 0 | 0 | 0 | 0 | 0 | 0 | 0 |
| **CER2_24ter** | 0 | 0 | 0 | 0 | 0 | 0 | 0 | 0 | 0 |
| **CER2_26ter** | 0 | 0 | 0 | 0 | 0 | 0 | 0 | 0 | 0 |
| **CERH126** | 0 | 0 | 0 | 0 | 0 | 0 | 0 | 0 | 0 |
| **CERS124** | 0 | 0 | 0 | 0 | 0 | 0 | 0 | 0 | 0 |
| **CERS126** | 0 | 0 | 0 | 0 | 0 | 0 | 0 | 0 | 0 |
| **CERS226** | 0 | 0 | 0 | 0 | 0 | 0 | 0 | 0 | 0 |
| **CERS324** | 0 | 0 | 0 | 0 | 0 | 0 | 0 | 0 | 0 |
| **CERS326** | 0 | 0 | 0 | 0 | 0 | 0 | 0 | 0 | 0 |
| **CHLSTI** | 0 | 0.01 | 0.01 | 0 | 0.01 | 0.01 | 0 | 0.02 | 0.02 |
| **CHRPL** | 0 | 0 | 0 | 0 | 0 | 0 | 0 | 0 | 0 |
| **CITtcp** | 0 | 0 | 0 | 0 | 0 | 0 | 0 | 0 | 0 |
| **CO2tp** | 0 | 0 | 0 | 0 | 0 | 0 | 0 | 0 | 0 |
| **COUCOAFm** | 0 | 0 | 0 | 0 | 0 | 0 | 0 | 0 | 0 |
| **CRNtp** | 0 | 0 | 0 | 0 | 0 | 0 | 0 | 0 | 0 |
| **CSNATp** | 0 | 0 | 0 | 0 | 0 | 0 | 0 | 0 | 0 |
| **CSm** | 0.12 | 0.46 | 0.34 | 0.14 | 0.59 | 0.45 | 0.17 | 0.63 | 0.46 |
| **CYOOm** | 0.01 | 0.29 | 0.28 | 0 | 0.29 | 0.29 | 0 | 0.29 | 0.29 |
| **CYOR_u6m** | 0 | 0.59 | 0.59 | 0 | 0.58 | 0.58 | 0 | 0.58 | 0.58 |
| **CYSTLp** | 0 | 0 | 0 | 0 | 0 | 0 | 0 | 0 | 0 |
| **CYSTtp** | 0 | 0 | 0 | 0 | 0 | 0 | 0 | 0 | 0 |
| **DB4PS** | 0 | 0 | 0 | 0 | 0 | 0 | 0 | 0 | 0 |
| **DESAT14** | 0 | 0 | 0 | 0 | 0 | 0 | 0 | 0 | 0 |
| **DESAT16** | 0 | 0 | 0 | 0 | 0 | 0 | 0 | 0 | 0 |
| **DESAT18** | 0 | 0 | 0 | 0 | 0 | 0 | 0 | 0 | 0 |
| **DESAT18_2** | 0 | 0 | 0 | 0 | 0 | 0 | 0 | 0 | 0 |
| **DHPSm** | 0 | 0 | 0 | 0 | 0 | 0 | 0 | 0 | 0 |
| **DIAT** | 0 | 0.03 | 0.03 | 0 | 0.04 | 0.04 | 0 | 0.05 | 0.05 |
| **DMATT** | 0 | 0.04 | 0.04 | 0 | 0.05 | 0.05 | 0 | 0.06 | 0.06 |
| **DNMPPA** | 0 | 0 | 0 | 0 | 0 | 0 | 0 | 0 | 0 |
| **DNTPPA** | 0 | 0 | 0 | 0 | 0 | 0 | 0 | 0 | 0 |
| **DOLPMMer** | 0.1 | 0.11 | 0.01 | 0.11 | 0.12 | 0.01 | 0.14 | 0.15 | 0.01 |
| **DOLPMTcer** | 0.1 | 0.11 | 0.01 | 0.11 | 0.12 | 0.01 | 0.14 | 0.15 | 0.01 |
| **DOLPt2er** | -0.11 | -0.1 | 0.01 | -0.12 | -0.11 | 0.01 | -0.15 | -0.14 | 0.01 |
| **DPCOAKm** | 0 | 0 | 0 | 0 | 0 | 0 | 0 | 0 | 0 |
| **DROPPRy** | 0 | 0 | 0 | 0 | 0 | 0 | 0 | 0 | 0 |
| **DRTPPD** | 0 | 0 | 0 | 0 | 0 | 0 | 0 | 0 | 0 |
| **ECOAH11p** | 0 | 0 | 0 | 0 | 0 | 0 | 0 | 0 | 0 |
| **ECOAH4p** | 0 | 0 | 0 | 0 | 0 | 0 | 0 | 0 | 0 |
| **ECOAH5p** | 0 | 0 | 0 | 0 | 0 | 0 | 0 | 0 | 0 |
| **ECOAH6p** | 0 | 0 | 0 | 0 | 0 | 0 | 0 | 0 | 0 |
| **ECOAH7p** | 0 | 0 | 0 | 0 | 0 | 0 | 0 | 0 | 0 |
| **ECOAH8p** | 0 | 0 | 0 | 0 | 0 | 0 | 0 | 0 | 0 |
| **EPISTAT_SC** | 0 | 0 | 0 | 0 | 0 | 0 | 0 | 0 | 0 |
| **EPISTESTH_SC** | 0 | 0 | 0 | 0 | 0 | 0 | 0 | 0 | 0 |
| **EPISTt** | -0.01 | 0 | 0.01 | -0.01 | 0 | 0.01 | -0.02 | 0 | 0.02 |
| **ERGSTAT_SC** | 0 | 0 | 0 | 0 | 0 | 0 | 0 | 0 | 0 |
| **ERGSTESTH_SC** | 0 | 0 | 0 | 0 | 0 | 0 | 0 | 0 | 0 |
| **ERGSTt** | -0.01 | 0 | 0.01 | -0.01 | 0 | 0.01 | -0.02 | 0 | 0.02 |
| **ERGSTter** | 0 | 0.01 | 0.01 | 0 | 0.01 | 0.01 | 0 | 0.02 | 0.02 |
| **ERGTETROLter** | 0 | 0.01 | 0.01 | 0 | 0.01 | 0.01 | 0 | 0.02 | 0.02 |
| **FA100tp** | 0 | 0 | 0 | 0 | 0 | 0 | 0 | 0 | 0 |
| **FA120ACPH** | 0 | 0 | 0 | 0 | 0 | 0 | 0 | 0 | 0 |
| **FA120ACPtm** | 0 | 0 | 0 | 0 | 0 | 0 | 0 | 0 | 0 |
| **FA120tp** | 0 | 0 | 0 | 0 | 0 | 0 | 0 | 0 | 0 |
| **FA140ACPH** | 0 | 0 | 0 | 0 | 0 | 0 | 0 | 0 | 0 |
| **FA140ACPtm** | 0 | 0 | 0 | 0 | 0 | 0 | 0 | 0 | 0 |
| **FA140COAabcp** | 0 | 0 | 0 | 0 | 0 | 0 | 0 | 0 | 0 |
| **FA140tp** | 0 | 0 | 0 | 0 | 0 | 0 | 0 | 0 | 0 |
| **FA141ACPH** | 0 | 0 | 0 | 0 | 0 | 0 | 0 | 0 | 0 |
| **FA141ACPtm** | 0 | 0 | 0 | 0 | 0 | 0 | 0 | 0 | 0 |
| **FA141COAabcp** | 0 | 0 | 0 | 0 | 0 | 0 | 0 | 0 | 0 |
| **FA141tp** | 0 | 0 | 0 | 0 | 0 | 0 | 0 | 0 | 0 |
| **FA160ACPH** | 0 | 0 | 0 | 0 | 0 | 0 | 0 | 0 | 0 |
| **FA160ACPtm** | 0 | 0 | 0 | 0 | 0 | 0 | 0 | 0 | 0 |
| **FA160COAabcp** | 0 | 0 | 0 | 0 | 0 | 0 | 0 | 0 | 0 |
| **FA160tp** | 0 | 0 | 0 | 0 | 0 | 0 | 0 | 0 | 0 |
| **FA161ACPH** | 0 | 0 | 0 | 0 | 0 | 0 | 0 | 0 | 0 |
| **FA161ACPtm** | 0 | 0 | 0 | 0 | 0 | 0 | 0 | 0 | 0 |
| **FA161COAabcp** | 0 | 0 | 0 | 0 | 0 | 0 | 0 | 0 | 0 |
| **FA161tp** | 0 | 0 | 0 | 0 | 0 | 0 | 0 | 0 | 0 |
| **FA180ACPH** | 0 | 0 | 0 | 0 | 0 | 0 | 0 | 0 | 0 |
| **FA180ACPtm** | 0 | 0 | 0 | 0 | 0 | 0 | 0 | 0 | 0 |
| **FA180COAabcp** | 0 | 0 | 0 | 0 | 0 | 0 | 0 | 0 | 0 |
| **FA181ACPH** | 0 | 0 | 0 | 0 | 0 | 0 | 0 | 0 | 0 |
| **FA181ACPtm** | 0 | 0 | 0 | 0 | 0 | 0 | 0 | 0 | 0 |
| **FA181COAabcp** | 0 | 0 | 0 | 0 | 0 | 0 | 0 | 0 | 0 |
| **FA182ACPH** | 0 | 0 | 0 | 0 | 0 | 0 | 0 | 0 | 0 |
| **FA182ACPtm** | 0 | 0 | 0 | 0 | 0 | 0 | 0 | 0 | 0 |
| **FA182COAabcp** | 0 | 0 | 0 | 0 | 0 | 0 | 0 | 0 | 0 |
| **FA240tp** | 0 | 0 | 0 | 0 | 0 | 0 | 0 | 0 | 0 |
| **FA80tp** | 0 | 0 | 0 | 0 | 0 | 0 | 0 | 0 | 0 |
| **FACOAL100p** | 0 | 0 | 0 | 0 | 0 | 0 | 0 | 0 | 0 |
| **FACOAL120p** | 0 | 0 | 0 | 0 | 0 | 0 | 0 | 0 | 0 |
| **FACOAL140p** | 0 | 0 | 0 | 0 | 0 | 0 | 0 | 0 | 0 |
| **FACOAL141** | 0 | 0 | 0 | 0 | 0 | 0 | 0 | 0 | 0 |
| **FACOAL141p** | 0 | 0 | 0 | 0 | 0 | 0 | 0 | 0 | 0 |
| **FACOAL160p** | 0 | 0 | 0 | 0 | 0 | 0 | 0 | 0 | 0 |
| **FACOAL161p** | 0 | 0 | 0 | 0 | 0 | 0 | 0 | 0 | 0 |
| **FACOAL180** | 0 | 0.02 | 0.02 | 0 | 0.02 | 0.02 | 0 | 0.03 | 0.03 |
| **FACOAL182** | 0 | 0.03 | 0.03 | 0 | 0.03 | 0.03 | 0 | 0.04 | 0.04 |
| **FACOAL240p** | 0 | 0 | 0 | 0 | 0 | 0 | 0 | 0 | 0 |
| **FACOAL260p** | 0 | 0 | 0 | 0 | 0 | 0 | 0 | 0 | 0 |
| **FACOAL80p** | 0 | 0 | 0 | 0 | 0 | 0 | 0 | 0 | 0 |
| **FADFMNtm** | 0 | 0 | 0 | 0 | 0 | 0 | 0 | 0 | 0 |
| **FAO141p_even** | 0 | 0 | 0 | 0 | 0 | 0 | 0 | 0 | 0 |
| **FAO141p_odd** | 0 | 0 | 0 | 0 | 0 | 0 | 0 | 0 | 0 |
| **FAO161p_even** | 0 | 0 | 0 | 0 | 0 | 0 | 0 | 0 | 0 |
| **FAO161p_odd** | 0 | 0 | 0 | 0 | 0 | 0 | 0 | 0 | 0 |
| **FAO181p_even** | 0 | 0 | 0 | 0 | 0 | 0 | 0 | 0 | 0 |
| **FAO181p_odd** | 0 | 0 | 0 | 0 | 0 | 0 | 0 | 0 | 0 |
| **FAO182p_even/even** | 0 | 0 | 0 | 0 | 0 | 0 | 0 | 0 | 0 |
| **FAO182p_even/odd** | 0 | 0 | 0 | 0 | 0 | 0 | 0 | 0 | 0 |
| **FAO182p_odd/odd** | 0 | 0 | 0 | 0 | 0 | 0 | 0 | 0 | 0 |
| **FAO240p** | 0 | 0 | 0 | 0 | 0 | 0 | 0 | 0 | 0 |
| **FAO80p** | 0 | 0 | 0 | 0 | 0 | 0 | 0 | 0 | 0 |
| **FAS140ACPm** | 0 | 0 | 0 | 0 | 0 | 0 | 0 | 0 | 0 |
| **FAS141** | 0 | 0 | 0 | 0 | 0 | 0 | 0 | 0 | 0 |
| **FAS141ACPm** | 0 | 0 | 0 | 0 | 0 | 0 | 0 | 0 | 0 |
| **FAS160** | 0 | 0.01 | 0.01 | 0 | 0.01 | 0.01 | 0 | 0.01 | 0.01 |
| **FAS160ACPm** | 0 | 0 | 0 | 0 | 0 | 0 | 0 | 0 | 0 |
| **FAS160COA** | 0 | 0.01 | 0.01 | 0 | 0.01 | 0.01 | 0 | 0.01 | 0.01 |
| **FAS161** | 0 | 0 | 0 | 0 | 0 | 0 | 0 | 0 | 0 |
| **FAS161ACPm** | 0 | 0 | 0 | 0 | 0 | 0 | 0 | 0 | 0 |
| **FAS180** | 0 | 0 | 0 | 0 | 0 | 0 | 0 | 0 | 0 |
| **FAS180ACPm** | 0 | 0 | 0 | 0 | 0 | 0 | 0 | 0 | 0 |
| **FAS180COA** | 0 | 0 | 0 | 0 | 0 | 0 | 0 | 0 | 0 |
| **FAS181** | 0 | 0 | 0 | 0 | 0 | 0 | 0 | 0 | 0 |
| **FAS181ACPm** | 0 | 0 | 0 | 0 | 0 | 0 | 0 | 0 | 0 |
| **FAS182ACPm** | 0 | 0 | 0 | 0 | 0 | 0 | 0 | 0 | 0 |
| **FAS240_L** | 0 | 0 | 0 | 0 | 0 | 0 | 0 | 0 | 0 |
| **FAS260** | 0 | 0 | 0 | 0 | 0 | 0 | 0 | 0 | 0 |
| **FBA2** | 0 | 0 | 0 | 0 | 0 | 0 | 0 | 0 | 0 |
| **FECOSTAT_SC** | 0 | 0 | 0 | 0 | 0 | 0 | 0 | 0 | 0 |
| **FECOSTESTH_SC** | 0 | 0 | 0 | 0 | 0 | 0 | 0 | 0 | 0 |
| **FECOSTt** | -0.01 | 0 | 0.01 | -0.01 | 0 | 0.01 | -0.02 | 0 | 0.02 |
| **FMNAT** | 0 | 0 | 0 | 0 | 0 | 0 | 0 | 0 | 0 |
| **FMNATm** | 0 | 0 | 0 | 0 | 0 | 0 | 0 | 0 | 0 |
| **FOLD3m** | 0 | 0 | 0 | 0 | 0 | 0 | 0 | 0 | 0 |
| **FRTT** | 0 | 0 | 0 | 0 | 0 | 0 | 0 | 0 | 0 |
| **FRUK** | 0 | 0 | 0 | 0 | 0 | 0 | 0 | 0 | 0 |
| **G6PDH2er** | 0 | 0.02 | 0.02 | 0 | 0.03 | 0.03 | 0 | 0.03 | 0.03 |
| **G6Pter** | 0 | 0.02 | 0.02 | 0 | 0.03 | 0.03 | 0 | 0.03 | 0.03 |
| **GCALDt** | 0 | 0 | 0 | 0 | 0 | 0 | 0 | 0 | 0 |
| **GGTT** | 0 | 0 | 0 | 0 | 0 | 0 | 0 | 0 | 0 |
| **GK1** | -500 | 500 | 1000 | -500 | 500 | 1000 | -500 | 500 | 1000 |
| **GK2** | -500 | 500 | 1000 | -500 | 500 | 1000 | -500 | 500 | 1000 |
| **GLUDC** | 0 | 0.3 | 0.3 | 0 | 0.41 | 0.41 | 0 | 0.41 | 0.41 |
| **GLXt** | 0 | 0.3 | 0.3 | 0 | 0.4 | 0.4 | 0 | 0.41 | 0.41 |
| **GLXtp** | 0 | 0 | 0 | 0 | 0 | 0 | 0 | 0 | 0 |
| **GRTT** | 0 | 0.04 | 0.04 | 0 | 0.05 | 0.05 | 0 | 0.06 | 0.06 |
| **GTPCII** | 0 | 0 | 0 | 0 | 0 | 0 | 0 | 0 | 0 |
| **H2Oter** | -0.02 | 0 | 0.02 | -0.03 | 0 | 0.03 | -0.03 | 0 | 0.03 |
| **H2Otp** | 0 | 0 | 0 | 0 | 0 | 0 | 0 | 0 | 0 |
| **HACD10p** | 0 | 0 | 0 | 0 | 0 | 0 | 0 | 0 | 0 |
| **HACD4p** | 0 | 0 | 0 | 0 | 0 | 0 | 0 | 0 | 0 |
| **HACD5p** | 0 | 0 | 0 | 0 | 0 | 0 | 0 | 0 | 0 |
| **HACD6p** | 0 | 0 | 0 | 0 | 0 | 0 | 0 | 0 | 0 |
| **HACD7p** | 0 | 0 | 0 | 0 | 0 | 0 | 0 | 0 | 0 |
| **HACD8p** | 0 | 0 | 0 | 0 | 0 | 0 | 0 | 0 | 0 |
| **HBZOPT6m** | 0 | 0 | 0 | 0 | 0 | 0 | 0 | 0 | 0 |
| **HCO3Em** | 0 | 0 | 0 | 0 | 0 | 0 | 0 | 0 | 0 |
| **HCYSt2p** | 0 | 0 | 0 | 0 | 0 | 0 | 0 | 0 | 0 |
| **HEXCCOAtx** | 0 | 0 | 0 | 0 | 0 | 0 | 0 | 0 | 0 |
| **HKYNH** | 0 | 0.02 | 0.02 | 0 | 0.03 | 0.03 | 0 | 0.03 | 0.03 |
| **HPPKm** | 0 | 0 | 0 | 0 | 0 | 0 | 0 | 0 | 0 |
| **ICDHyp** | 0 | 0 | 0 | 0 | 0 | 0 | 0 | 0 | 0 |
| **IPDDI** | 0 | 0.04 | 0.04 | 0 | 0.05 | 0.05 | 0 | 0.06 | 0.06 |
| **IPDPtm** | 0 | 0 | 0 | 0 | 0 | 0 | 0 | 0 | 0 |
| **KYN3OX** | 0 | 0.02 | 0.02 | 0 | 0.03 | 0.03 | 0 | 0.03 | 0.03 |
| **LANOSTAT_SC** | 0 | 0 | 0 | 0 | 0 | 0 | 0 | 0 | 0 |
| **LANOSTESTH_SC** | 0 | 0 | 0 | 0 | 0 | 0 | 0 | 0 | 0 |
| **LANOSTt** | -0.02 | 0 | 0.02 | -0.03 | 0 | 0.03 | -0.03 | 0 | 0.03 |
| **LNS14DM** | 0 | 0.01 | 0.01 | 0 | 0.02 | 0.02 | 0 | 0.02 | 0.02 |
| **LNS14DMx** | 0 | 0.01 | 0.01 | 0 | 0.02 | 0.02 | 0 | 0.02 | 0.02 |
| **LNSTLS** | 0 | 0.02 | 0.02 | 0 | 0.03 | 0.03 | 0 | 0.03 | 0.03 |
| **MALOAAtp** | 0 | 0 | 0 | 0 | 0 | 0 | 0 | 0 | 0 |
| **MALS** | 0 | 0.2 | 0.2 | 0 | 0.28 | 0.28 | 0 | 0.28 | 0.28 |
| **MALSp** | 0 | 0 | 0 | 0 | 0 | 0 | 0 | 0 | 0 |
| **MAN1PT** | 0.1 | 0.11 | 0.01 | 0.11 | 0.12 | 0.01 | 0.14 | 0.15 | 0.01 |
| **MAN6PI** | -0.11 | -0.1 | 0.01 | -0.12 | -0.11 | 0.01 | -0.15 | -0.14 | 0.01 |
| **MANNANter** | -0.11 | -0.1 | 0.01 | -0.12 | -0.11 | 0.01 | -0.15 | -0.14 | 0.01 |
| **MCOATAm** | 0 | 0 | 0 | 0 | 0 | 0 | 0 | 0 | 0 |
| **MDHp** | 0 | 0 | 0 | 0 | 0 | 0 | 0 | 0 | 0 |
| **MI1PS** | 0 | 0 | 0 | 0 | 0 | 0 | 0 | 0 | 0 |
| **NADPt** | 0 | 0.04 | 0.04 | 0 | 0.04 | 0.04 | 0 | 0.05 | 0.05 |
| **NADS1** | 0 | 0.04 | 0.04 | 0 | 0.04 | 0.04 | 0 | 0.05 | 0.05 |
| **NH4tp** | 0 | 0 | 0 | 0 | 0 | 0 | 0 | 0 | 0 |
| **NMNATm** | 0 | 0 | 0 | 0 | 0 | 0 | 0 | 0 | 0 |
| **NMNt2m** | 0 | 0 | 0 | 0 | 0 | 0 | 0 | 0 | 0 |
| **NNAT** | 0 | 0.04 | 0.04 | 0 | 0.04 | 0.04 | 0 | 0.05 | 0.05 |
| **NNDPR** | 0 | 0.04 | 0.04 | 0 | 0.04 | 0.04 | 0 | 0.05 | 0.05 |
| **O2t** | 0.02 | 0.3 | 0.28 | 0.01 | 0.3 | 0.29 | 0.01 | 0.3 | 0.29 |
| **O2ter** | 0 | 0.02 | 0.02 | 0 | 0.03 | 0.03 | 0 | 0.03 | 0.03 |
| **O2tm** | 0.01 | 0.29 | 0.28 | 0 | 0.29 | 0.29 | 0 | 0.29 | 0.29 |
| **PAtm_SC** | 0 | 0.01 | 0.01 | 0 | 0.01 | 0.01 | 0 | 0.01 | 0.01 |
| **PCDAGAT** | 0 | 0 | 0 | 0 | 0 | 0 | 0 | 0 | 0 |
| **PENDPtm** | 0 | 0 | 0 | 0 | 0 | 0 | 0 | 0 | 0 |
| **PEtm_SC** | 0 | 0 | 0 | 0 | 0 | 0 | 0 | 0 | 0 |
| **PEtv_SC** | 0 | 0 | 0 | 0 | 0 | 0 | 0 | 0 | 0 |
| **PIt2p** | 0 | 0 | 0 | 0 | 0 | 0 | 0 | 0 | 0 |
| **PMANM** | -0.11 | -0.1 | 0.01 | -0.12 | -0.11 | 0.01 | -0.15 | -0.14 | 0.01 |
| **PMDPHT** | 0 | 0 | 0 | 0 | 0 | 0 | 0 | 0 | 0 |
| **PNTK** | 0 | 0 | 0 | 0 | 0 | 0 | 0 | 0 | 0 |
| **POLYAO2** | 0 | 0.03 | 0.03 | 0 | 0.04 | 0.04 | 0 | 0.05 | 0.05 |
| **PPCDC** | 0 | 0 | 0 | 0 | 0 | 0 | 0 | 0 | 0 |
| **PPNCL2** | 0 | 0 | 0 | 0 | 0 | 0 | 0 | 0 | 0 |
| **PPTTm** | 0 | 0 | 0 | 0 | 0 | 0 | 0 | 0 | 0 |
| **PSPHS** | 0 | 0 | 0 | 0 | 0 | 0 | 0 | 0 | 0 |
| **PStm_SC** | -0.01 | 0 | 0.01 | -0.01 | 0 | 0.01 | -0.01 | 0 | 0.01 |
| **PStv_SC** | 0 | 0 | 0 | 0 | 0 | 0 | 0 | 0 | 0 |
| **PTE10x** | 0 | 0 | 0 | 0 | 0 | 0 | 0 | 0 | 0 |
| **PTE11x** | 0 | 0 | 0 | 0 | 0 | 0 | 0 | 0 | 0 |
| **PTE12x** | 0 | 0 | 0 | 0 | 0 | 0 | 0 | 0 | 0 |
| **PTE2x** | 0 | 0 | 0 | 0 | 0 | 0 | 0 | 0 | 0 |
| **PTE7x** | 0 | 0 | 0 | 0 | 0 | 0 | 0 | 0 | 0 |
| **PTE9x** | 0 | 0 | 0 | 0 | 0 | 0 | 0 | 0 | 0 |
| **PTPATim** | 0 | 0 | 0 | 0 | 0 | 0 | 0 | 0 | 0 |
| **PYRDC2** | 0 | 1.26 | 1.26 | 0 | 1.74 | 1.74 | 0 | 1.73 | 1.73 |
| **PYRt2p** | 0 | 0 | 0 | 0 | 0 | 0 | 0 | 0 | 0 |
| **QUILSYN** | 0 | 0.02 | 0.02 | 0 | 0.03 | 0.03 | 0 | 0.03 | 0.03 |
| **QULNS** | 0 | 0.04 | 0.04 | 0 | 0.04 | 0.04 | 0 | 0.05 | 0.05 |
| **RBFSa** | 0 | 0 | 0 | 0 | 0 | 0 | 0 | 0 | 0 |
| **RBFSb** | 0 | 0 | 0 | 0 | 0 | 0 | 0 | 0 | 0 |
| **SAM24MT** | 0 | 0.01 | 0.01 | 0 | 0.01 | 0.01 | 0 | 0.02 | 0.02 |
| **SQ23EPXter** | 0 | 0.02 | 0.02 | 0 | 0.03 | 0.03 | 0 | 0.03 | 0.03 |
| **SQLEr** | 0 | 0.02 | 0.02 | 0 | 0.03 | 0.03 | 0 | 0.03 | 0.03 |
| **SQLS** | 0 | 0.02 | 0.02 | 0 | 0.03 | 0.03 | 0 | 0.03 | 0.03 |
| **SQLter** | 0 | 0.02 | 0.02 | 0 | 0.03 | 0.03 | 0 | 0.03 | 0.03 |
| **SSALy** | 0 | 0.3 | 0.3 | 0 | 0.41 | 0.41 | 0 | 0.41 | 0.41 |
| **SUCOASm** | -0.3 | 0 | 0.3 | -0.41 | 0 | 0.41 | -0.41 | 0 | 0.41 |
| **T4HCINNMFM** | 0 | 0 | 0 | 0 | 0 | 0 | 0 | 0 | 0 |
| **THZPSN1_SC** | 0 | 0 | 0 | 0 | 0 | 0 | 0 | 0 | 0 |
| **THZPSN2_SC** | 0 | 0 | 0 | 0 | 0 | 0 | 0 | 0 | 0 |
| **TTCCOAtx** | 0 | 0 | 0 | 0 | 0 | 0 | 0 | 0 | 0 |
| **TYRt2p** | 0 | 0 | 0 | 0 | 0 | 0 | 0 | 0 | 0 |
| **ZYMSTAT_SC** | 0 | 0 | 0 | 0 | 0 | 0 | 0 | 0 | 0 |
| **ZYMSTESTH_SC** | 0 | 0 | 0 | 0 | 0 | 0 | 0 | 0 | 0 |
| **ZYMSTt** | -0.01 | 0 | 0.01 | -0.02 | 0 | 0.02 | -0.02 | 0 | 0.02 |

* mmol/gDCW/h
