# Supplementary material for: Genome-Scale Consequences of Cofactor Balancing in Engineered Pentose Utilization Pathways in Saccharomyces cerevisiae
Source: PLoS One. 2011 Nov 4;6(11):e27316. doi: 10.1371/journal.pone.0027316 (PMC3208632; doi:10.1371/journal.pone.0027316)
Supplement: Table S2 — Based on the flux ratio limits Rmax (max v1/v2) and Rmin (min v1/v2) various types of coupling were identified for wild type, engineered cofactor imbalanced (ECI) and engineered cofactor balanced (ECB) models. (DOCX) [file pone.0027316.s002.docx]

**Table S2**. Based on the flux ratio limits R_max_ (max v_1_/ v_2_) and R_min_ (min v_1_/ v_2_) various types of coupling were identified for wild type, engineered cofactor imbalanced (ECI) and engineered cofactor balanced (ECB) models.

| **Reaction**  **(ν_1_)** | **Reaction**  **(ν_2_)** | **Wild type model** | | | **Engineered cofactor**  **imbalanced model** | | | **Engineered cofactor**  **balanced model** | | |
| --- | --- | --- | --- | --- | --- | --- | --- | --- | --- | --- |
|  |  | **Max**  **(ν_1_/ν_2_)** | **Min**  **(ν_1_/ν_2_)** | **Coupled**  **Rxns** | **Max**  **(ν_1_/ν_2_)** | **Min**  **(ν_1_/ν_2_)** | **Coupled**  **Rxns** | **Max**  **(ν_1_/ν_2_)** | **Min**  **(ν_1_/ν_2_)** | **Coupled**  **Rxns** |
| DHAK | ENO | 12.12 | 1.09 | Partially | 17.00 | 0.88 | Partially | 17.00 | 1.09 | Partially |
| DHAK | G3PD1ir | 9.72 | 0.00 | Directionally | 13.17 | 0.00 | Directionally | 13.41 | 0.00 | Directionally |
| DHAK | G3PT | 11.87 | 1.00 | Partially | 14.46 | 1.00 | Partially | 16.45 | 1.00 | Partially |
| DHAK | GAPD | 12.12 | 1.09 | Partially | 17.00 | 0.88 | Partially | 17.00 | 1.09 | Partially |
| DHAK | GLYCDy | 1.00 | 1.00 | Fully | 1.00 | 1.00 | Fully | 1.00 | 1.00 | Fully |
| DHAK | PGK | 12.12 | 1.09 | Partially | 17.00 | 0.88 | Partially | 17.00 | 1.09 | Partially |
| DHAK | PGM | 12.12 | 1.09 | Partially | 17.00 | 0.88 | Partially | 17.00 | 1.09 | Partially |
| DHAK | PYK | 22.99 | 1.09 | Partially | 29.11 | 0.88 | Partially | 32.17 | 1.09 | Partially |
| DHAK | PYRDC | 12.52 | 0.00 | Directionally | 15.64 | 0.00 | Directionally | 17.02 | 0.00 | Directionally |
| ENO | DHAK | 0.90 | 0.00 | Directionally | 1.13 | 0.00 | Directionally | 0.90 | 0.00 | Directionally |
| ENO | G3PD1ir | 1.28 | 0.00 | Directionally | 1.28 | 0.00 | Directionally | 1.28 | 0.00 | Directionally |
| ENO | G3PT | 0.90 | 0.00 | Directionally | 1.13 | 0.00 | Directionally | 0.90 | 0.00 | Directionally |
| ENO | GAPD | 2.28 | 1.00 | Partially | 4.01 | 1.00 | Partially | 2.28 | 1.00 | Partially |
| ENO | GLYCDy | 0.90 | 0.00 | Directionally | 1.13 | 0.00 | Directionally | 0.90 | 0.00 | Directionally |
| ENO | PGK | 2.28 | 1.00 | Partially | 4.01 | 1.00 | Partially | 2.28 | 1.00 | Partially |
| ENO | PGM | -1.00 | -1.00 | Fully | -1.00 | -1.00 | Fully | -1.00 | -1.00 | Fully |
| ENO | PYK | 1.77 | 0.63 | Partially | 2.02 | 0.51 | Partially | 1.77 | 0.63 | Partially |
| ENO | PYRDC | 2.28 | 0.00 | Directionally | 4.01 | 0.00 | Directionally | 2.28 | 0.00 | Directionally |
| G3PD1ir | DHAK | 2.00 | 0.00 | Directionally | 2.14 | 0.00 | Directionally | 2.38 | 0.00 | Directionally |
| G3PD1ir | ENO | 12.12 | 0.73 | Partially | 17.00 | 0.73 | Partially | 17.00 | 0.73 | Partially |
| G3PD1ir | G3PT | 11.87 | 0.00 | Directionally | 14.46 | 0.00 | Directionally | 16.45 | 0.00 | Directionally |
| G3PD1ir | GAPD | 12.12 | 0.73 | Partially | 17.00 | 0.73 | Partially | 17.00 | 0.73 | Partially |
| G3PD1ir | GLYCDy | 2.00 | 0.00 | Directionally | 2.14 | 0.00 | Directionally | 2.38 | 0.00 | Directionally |
| G3PD1ir | PGK | 12.12 | 0.73 | Partially | 17.00 | 0.73 | Partially | 17.00 | 0.73 | Partially |
| G3PD1ir | PGM | 12.12 | 0.73 | Partially | 17.00 | 0.73 | Partially | 17.00 | 0.73 | Partially |
| G3PD1ir | PYK | 23.52 | 0.73 | Partially | 29.11 | 0.73 | Partially | 32.37 | 0.73 | Partially |
| G3PD1ir | PYRDC | 12.52 | 0.00 | Directionally | 15.64 | 0.00 | Directionally | 17.02 | 0.00 | Directionally |
| G3PT | DHAK | 1.00 | 0.00 | Directionally | 1.00 | 0.00 | Directionally | 1.00 | 0.00 | Directionally |
| G3PT | ENO | 12.12 | 1.09 | Partially | 17.00 | 0.88 | Partially | 17.00 | 1.09 | Partially |
| G3PT | G3PD1ir | 9.16 | 0.00 | Directionally | 12.22 | 0.00 | Directionally | 12.91 | 0.00 | Directionally |
| G3PT | GAPD | 12.12 | 1.09 | Partially | 17.00 | 0.88 | Partially | 17.00 | 1.09 | Partially |
| G3PT | GLYCDy | 1.00 | 0.00 | Directionally | 1.00 | 0.00 | Directionally | 1.00 | 0.00 | Directionally |
| G3PT | PGK | 12.12 | 1.09 | Partially | 17.00 | 0.88 | Partially | 17.00 | 1.09 | Partially |
| G3PT | PGM | 12.12 | 1.09 | Partially | 17.00 | 0.88 | Partially | 17.00 | 1.09 | Partially |
| G3PT | PYK | 22.99 | 1.09 | Partially | 29.11 | 0.88 | Partially | 32.17 | 1.09 | Partially |
| G3PT | PYRDC | 12.52 | 0.00 | Directionally | 15.64 | 0.00 | Directionally | 17.02 | 0.00 | Directionally |
| GAPD | DHAK | 0.90 | 0.00 | Directionally | 1.13 | 0.00 | Directionally | 0.90 | 0.00 | Directionally |
| GAPD | ENO | 1.00 | 0.36 | Partially | 1.00 | 0.11 | Partially | 1.00 | 0.36 | Partially |
| GAPD | G3PD1ir | 1.28 | 0.00 | Directionally | 1.28 | 0.00 | Directionally | 1.28 | 0.00 | Directionally |
| GAPD | G3PT | 0.90 | 0.00 | Directionally | 1.13 | 0.00 | Directionally | 0.90 | 0.00 | Directionally |
| GAPD | GLYCDy | 0.90 | 0.00 | Directionally | 1.13 | 0.00 | Directionally | 0.90 | 0.00 | Directionally |
| GAPD | PGK | -1.00 | -1.00 | Fully | -1.00 | -1.00 | Fully | -1.00 | -1.00 | Fully |
| GAPD | PGM | 1.00 | 0.36 | Partially | 1.00 | 0.11 | Partially | 1.00 | 0.36 | Partially |
| GAPD | PYK | 1.77 | 0.36 | Partially | 2.02 | 0.11 | Partially | 1.77 | 0.36 | Partially |
| GAPD | PYRDC | 1.49 | 0.00 | Directionally | 1.49 | 0.00 | Directionally | 1.49 | 0.00 | Directionally |
| GLYCDy | DHAK | 1.00 | 1.00 | Fully | 1.00 | 1.00 | Fully | 1.00 | 1.00 | Fully |
| GLYCDy | ENO | 12.12 | 1.09 | Partially | 17.00 | 0.88 | Partially | 17.00 | 1.09 | Partially |
| GLYCDy | G3PD1ir | 9.72 | 0.00 | Directionally | 13.17 | 0.00 | Directionally | 13.41 | 0.00 | Directionally |
| GLYCDy | G3PT | 11.87 | 1.00 | Partially | 14.46 | 1.00 | Partially | 16.45 | 1.00 | Partially |
| GLYCDy | GAPD | 12.12 | 1.09 | Partially | 17.00 | 0.88 | Partially | 17.00 | 1.09 | Partially |
| GLYCDy | PGK | 12.12 | 1.09 | Partially | 17.00 | 0.88 | Partially | 17.00 | 1.09 | Partially |
| GLYCDy | PGM | 12.12 | 1.09 | Partially | 17.00 | 0.88 | Partially | 17.00 | 1.09 | Partially |
| GLYCDy | PYK | 22.99 | 1.09 | Partially | 29.11 | 0.88 | Partially | 32.17 | 1.09 | Partially |
| GLYCDy | PYRDC | 12.52 | 0.00 | Directionally | 15.64 | 0.00 | Directionally | 17.02 | 0.00 | Directionally |
| PGK | DHAK | 1.76 | 0.43 | Partially | 0.43 | 0.23 | Partially | 0.23 | 1.82 | Partially |
| PGK | ENO | 0.51 | 0.43 | Partially | 0.28 | 0.23 | Partially | 1.56 | 0.23 | Partially |
| PGK | G3PD1ir | 0.23 | 0.23 | Fully | 0.23 | 0.23 | Fully | 1.56 | 0.04 | Partially |
| PGK | G3PT | 1.76 | 0.23 | Partially | 0.23 | 0.43 | Partially | 0.23 | 0.23 | Fully |
| PGK | GAPD | 0.23 | 0.23 | Fully | 0.23 | 0.23 | Fully | 0.43 | 0.43 | Fully |
| PGK | GLYCDy | 1.76 | 0.43 | Partially | 0.43 | 0.23 | Partially | 0.23 | 1.82 | Partially |
| PGK | PGM | 0.43 | 0.51 | Partially | 0.23 | 0.83 | Partially | 0.23 | 1.77 | Partially |
| PGK | PYK | 0.23 | 0.43 | Partially | 0.13 | 0.51 | Partially | 0.43 | 0.13 | Partially |
| PGK | PYRDC | 0.43 | 0.43 | Fully | 0.23 | 0.43 | Partially | 0.23 | 0.43 | Partially |
| PGM | DHAK | 0.02 | 0.02 | Fully | 0.01 | 0.01 | Fully | 0.02 | 0.02 | Fully |
| PGM | ENO | 0.02 | 0.02 | Fully | 1.35 | 1.28 | Partially | 0.02 | 0.02 | Fully |
| PGM | G3PD1ir | 0.02 | 0.02 | Fully | 0.01 | 0.01 | Fully | 0.03 | 0.02 | Partially |
| PGM | G3PT | 0.02 | 0.02 | Fully | 0.01 | 0.01 | Fully | 0.02 | 0.02 | Fully |
| PGM | GAPD | 0.02 | 0.02 | Fully | 0.01 | 0.01 | Fully | 0.02 | 0.02 | Fully |
| PGM | GLYCDy | 0.02 | 0.02 | Fully | 0.01 | 0.01 | Fully | 0.02 | 0.02 | Fully |
| PGM | PGK | 0.02 | 0.02 | Fully | 0.01 | 0.01 | Fully | 0.02 | 0.02 | Fully |
| PGM | PYK | 0.02 | 0.02 | Fully | 0.01 | 0.01 | Fully | 0.02 | 0.02 | Fully |
| PGM | PYRDC | 0.02 | 0.02 | Fully | 0.02 | 0.01 | Partially | 0.02 | 0.03 | Partially |
| PYK | DHAK | 0.90 | 0.00 | Directionally | 1.13 | 0.00 | Directionally | 0.90 | 0.00 | Directionally |
| PYK | ENO | 1.71 | 0.62 | Partially | 1.95 | 0.49 | Partially | 1.71 | 0.62 | Partially |
| PYK | G3PD1ir | 1.28 | 0.00 | Directionally | 1.28 | 0.00 | Directionally | 1.28 | 0.00 | Directionally |
| PYK | G3PT | 0.90 | 0.00 | Directionally | 1.13 | 0.00 | Directionally | 0.90 | 0.00 | Directionally |
| PYK | GAPD | 2.28 | 0.62 | Partially | 4.01 | 0.49 | Partially | 2.28 | 0.62 | Partially |
| PYK | GLYCDy | 0.90 | 0.00 | Directionally | 1.13 | 0.00 | Directionally | 0.90 | 0.00 | Directionally |
| PYK | PGK | 2.28 | 0.62 | Partially | 4.01 | 0.49 | Partially | 2.28 | 0.62 | Partially |
| PYK | PGM | 1.71 | 0.62 | Partially | 1.95 | 0.49 | Partially | 1.71 | 0.62 | Partially |
| PYK | PYRDC | 2.28 | 0.00 | Directionally | 4.01 | 0.00 | Directionally | 2.28 | 0.00 | Directionally |
| PYRDC | DHAK | 11.87 | 0.00 | Directionally | 14.30 | 0.00 | Directionally | 16.97 | 0.00 | Directionally |
| PYRDC | ENO | 12.12 | 0.36 | Partially | 17.00 | 0.14 | Partially | 17.00 | 0.36 | Partially |
| PYRDC | G3PD1ir | 12.40 | 0.00 | Directionally | 17.11 | 0.00 | Directionally | 17.28 | 0.00 | Directionally |
| PYRDC | G3PT | 11.87 | 0.00 | Directionally | 14.30 | 0.00 | Directionally | 16.97 | 0.00 | Directionally |
| PYRDC | GAPD | 12.12 | 0.72 | Partially | 17.00 | 0.72 | Partially | 17.00 | 0.72 | Partially |
| PYRDC | GLYCDy | 11.87 | 0.00 | Directionally | 14.30 | 0.00 | Directionally | 16.97 | 0.00 | Directionally |
| PYRDC | PGK | 12.12 | 0.72 | Partially | 17.00 | 0.72 | Partially | 17.00 | 0.72 | Partially |
| PYRDC | PGM | 12.12 | 0.36 | Partially | 17.00 | 0.14 | Partially | 17.00 | 0.36 | Partially |
| PYRDC | PYK | 18.66 | 0.36 | Partially | 22.88 | 0.14 | Partially | 24.95 | 0.36 | Partially |
